# Supplementary material for: ABO: A 3D stroma-supported culture platform enabling full human B-lymphopoiesis for disease modeling and gene therapy development
Source: Cell Rep Med. 2026 Jun 18;7(7):102879. doi: 10.1016/j.xcrm.2026.102879 (PMC13400188; doi:10.1016/j.xcrm.2026.102879)
Supplement: Document S1. Figures S1–S7 and Table S1 [file mmc1.pdf]

Cell Reports Medicine, Volume *7*

Supplemental information

ABO: A 3D stroma-supported culture platform enabling full human B-lymphopoiesis for disease modeling and gene therapy development

Merijn Braams, Martijn Cordes, Sandra A. Vloemans, Bas de Mooij, Sandra de Bruin-Versteeg, Ashley Wachtmeester, Anton W. Langerak, Karin Pike-Overzet, Frank J.T. Staal, Kirsten Canté-Barrett, and Sander de Kivit

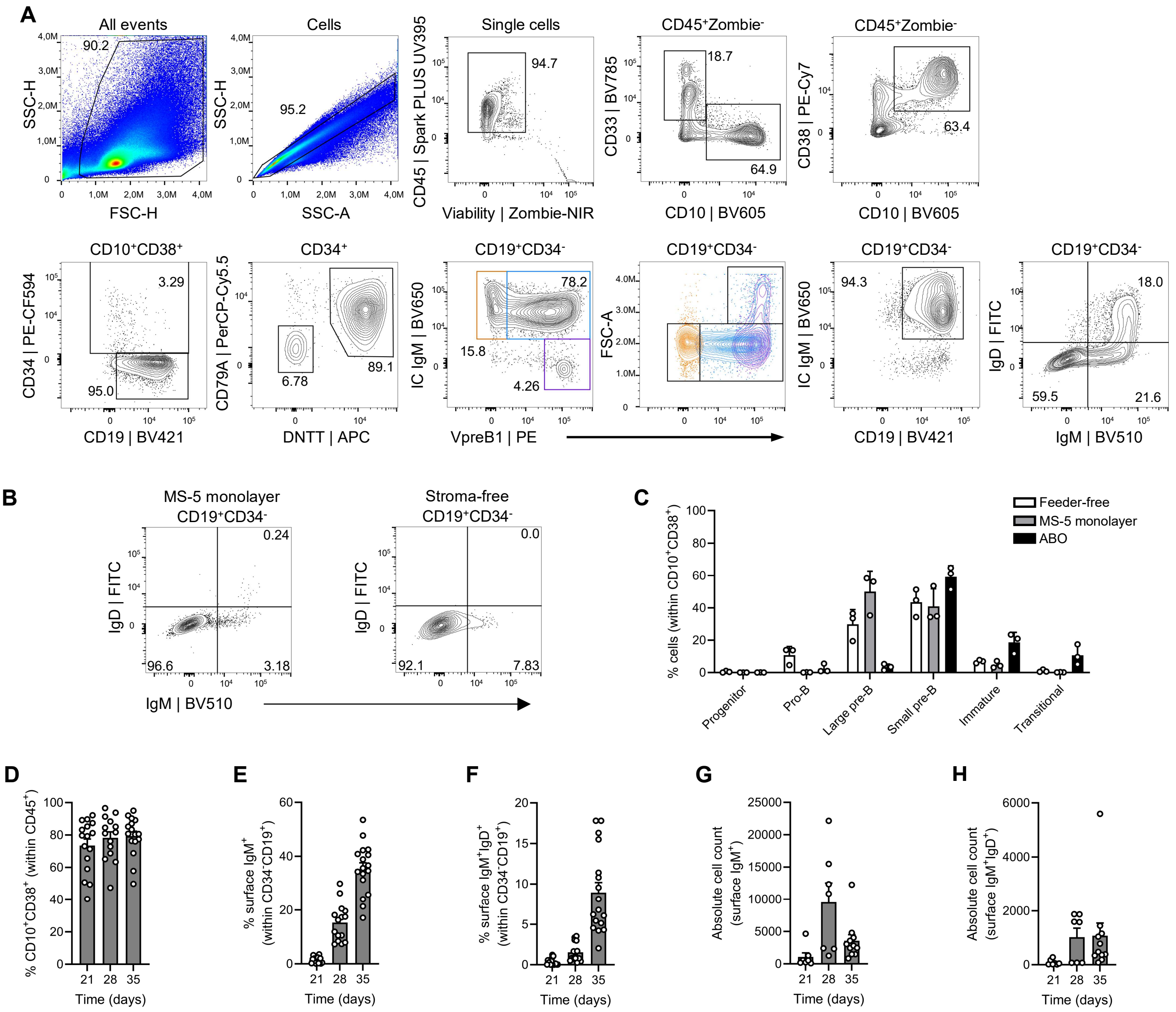

Figure S1. Characterization and reproducibility of B-cell development in ABOs. Related to Figure 1.
(**A**) Gating strategy used to identify the B-cell developmental stages.
(**B**) Representative flow cytometric plots showing the frequencies of IgM$^+$IgD$^-$ immature and IgM$^+$IgD$^+$ transitional B cells in day 35 cultures.
(**C**) Distribution of HSPCs developing along the B-cell developmental trajectory at day 35 of culture.
(**B, C**) Data represent n=3 individual donors in independent cultures.
(**D-F**) Quantification of the frequencies of lymphoid committed CD10$^+$CD38$^+$ (**D**), IgM$^+$ (**E**) and IgM$^+$IgD$^+$ cells in ABOs at indicated time points (**F**). Data represent n=17 individual donors in independent cultures.
(**G, H**) Quantification of absolute counts of IgM$^+$ (**G**) and IgM$^+$IgD$^+$ (**H**) cells harvested from 2 ABOs at indicated time points. Data represent n=11 individual donors in independent cultures.

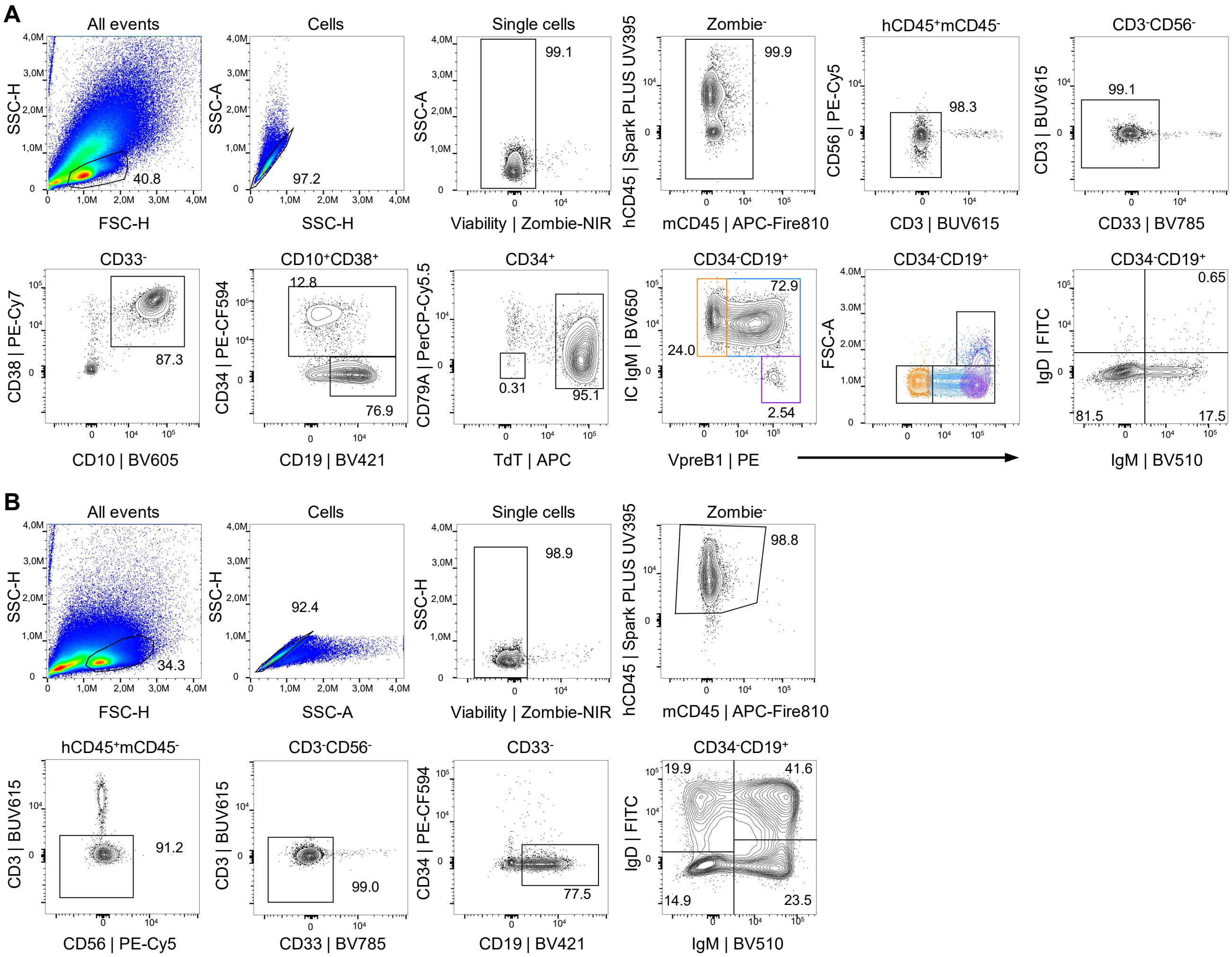

Figure S2. Flow cytometric analysis of the B-cell developmental stages in humanized NSG mice material. Related to Figure 2.

(**A, B**) Gating strategy used for the analysis of BM (**A**) and spleen (**B**). (mCD45, murine CD45; hCD45, human CD45)

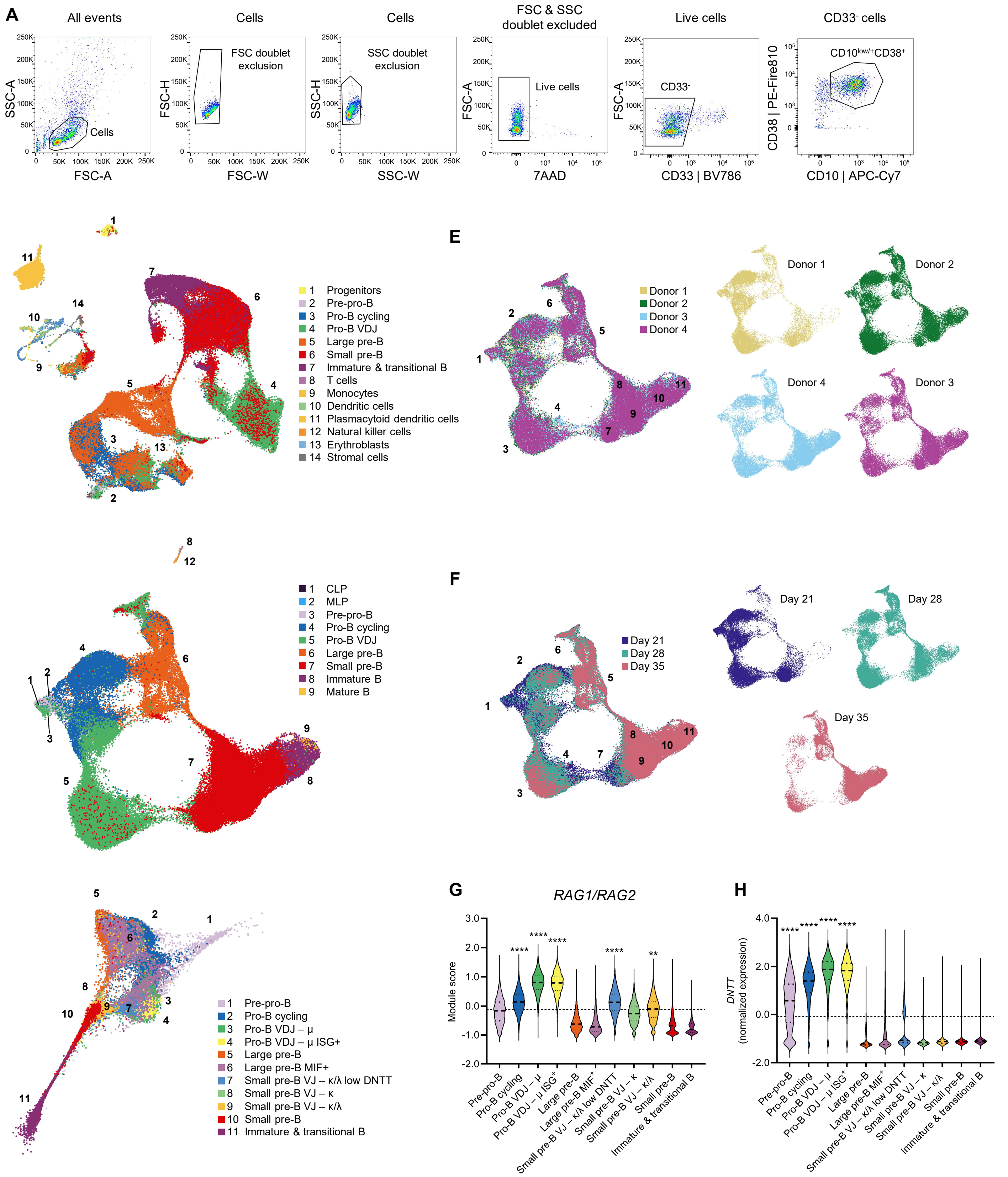

Figure S3. ScRNA-seq analysis of B-cell development in ABOs. Related to Figure 3.
(**A**) Gating strategy used for sorting of CD10$^+$CD38$^+$ cells from day 21-35 ABOs.
(**B, C**) Projection of clusters annotated as described by Zeng *et al*. [S1] onto the UMAP derived from scRNA-seq data of day 21-35 ABOs before subtracting non-B cells (**B**), and after filtering for B cells (**C**).
(**D**) Projection of scRNA-seq data of day 21-35 ABOs onto the UMAP derived of healthy human BM [S1].
(**E, F**) Projection of clusters annotated based on (**E**) individual donors and (**F**) time of ABO culture onto the UMAP of day 21-35 ABOs. Numbers represent clusters as identified in **Figure 3B**.
(**G, H**) Violin plots illustrating recombination activity (**G**) based on *RAG1/2* expression and relative expression of *DNTT* (**H**) within each cluster annotated. Statistical analysis was performed using a one-sample *t*-test (**** p<0.0001, **p<0.01, dashed line: average module score/expression level of the data set).
(**B-H**) Data represent n=4 individual donors in independent cultures.

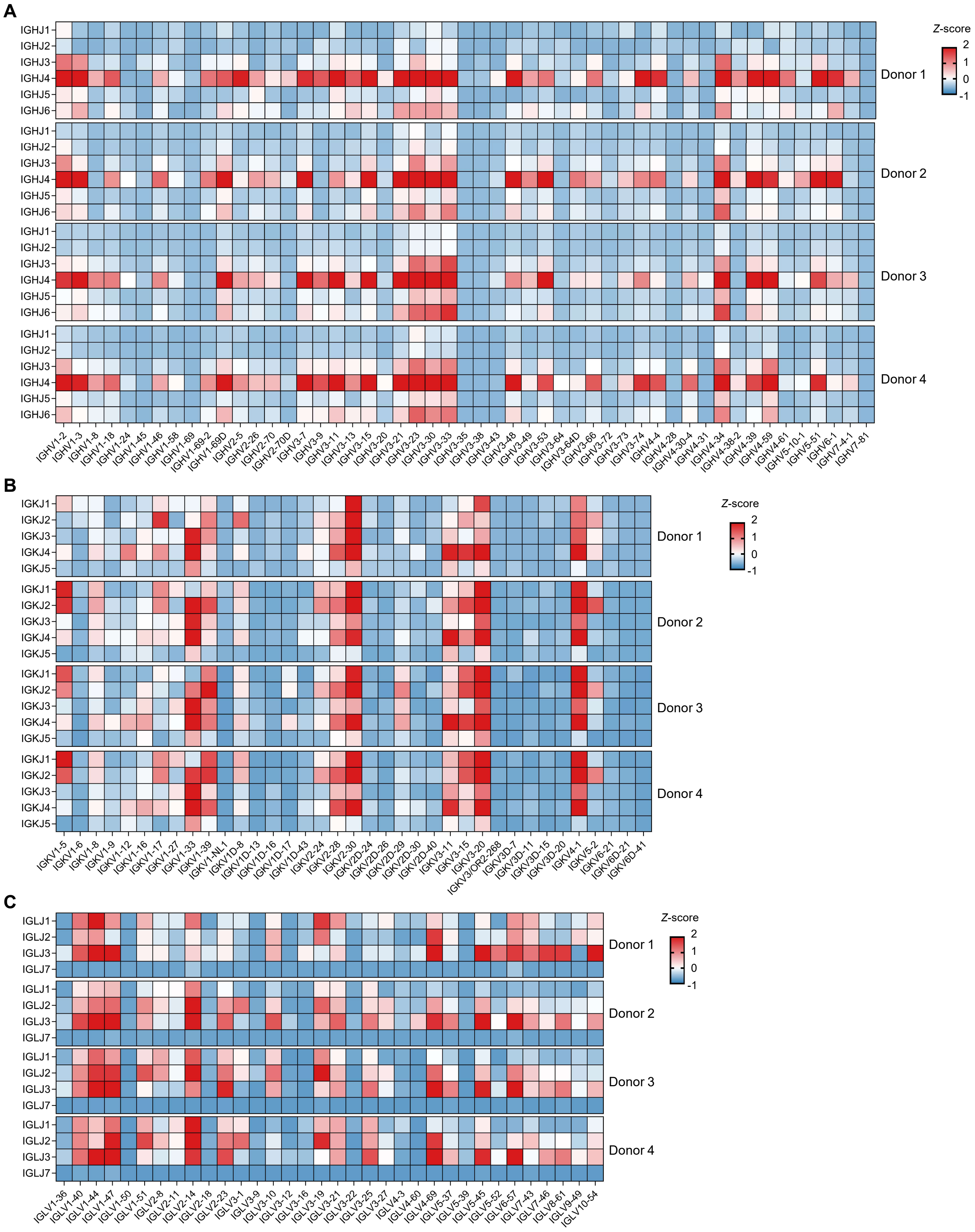

Figure S4. Analysis of V- and J-gene segments usage among individual donors. Related to Figure 4.
(**A-C**) Heatmaps showing V- and J-gene segment usage during Ig heavy *(IGH)* chain (**A**), Igκ (*IGK*) (**B**) and Igλ (*IGL*) light chain rearrangements (**C**), separated by each individual donor within immature and transitional B cells in day 35 ABOs. Z-scores calculated from V-J gene pair frequencies are color coded.

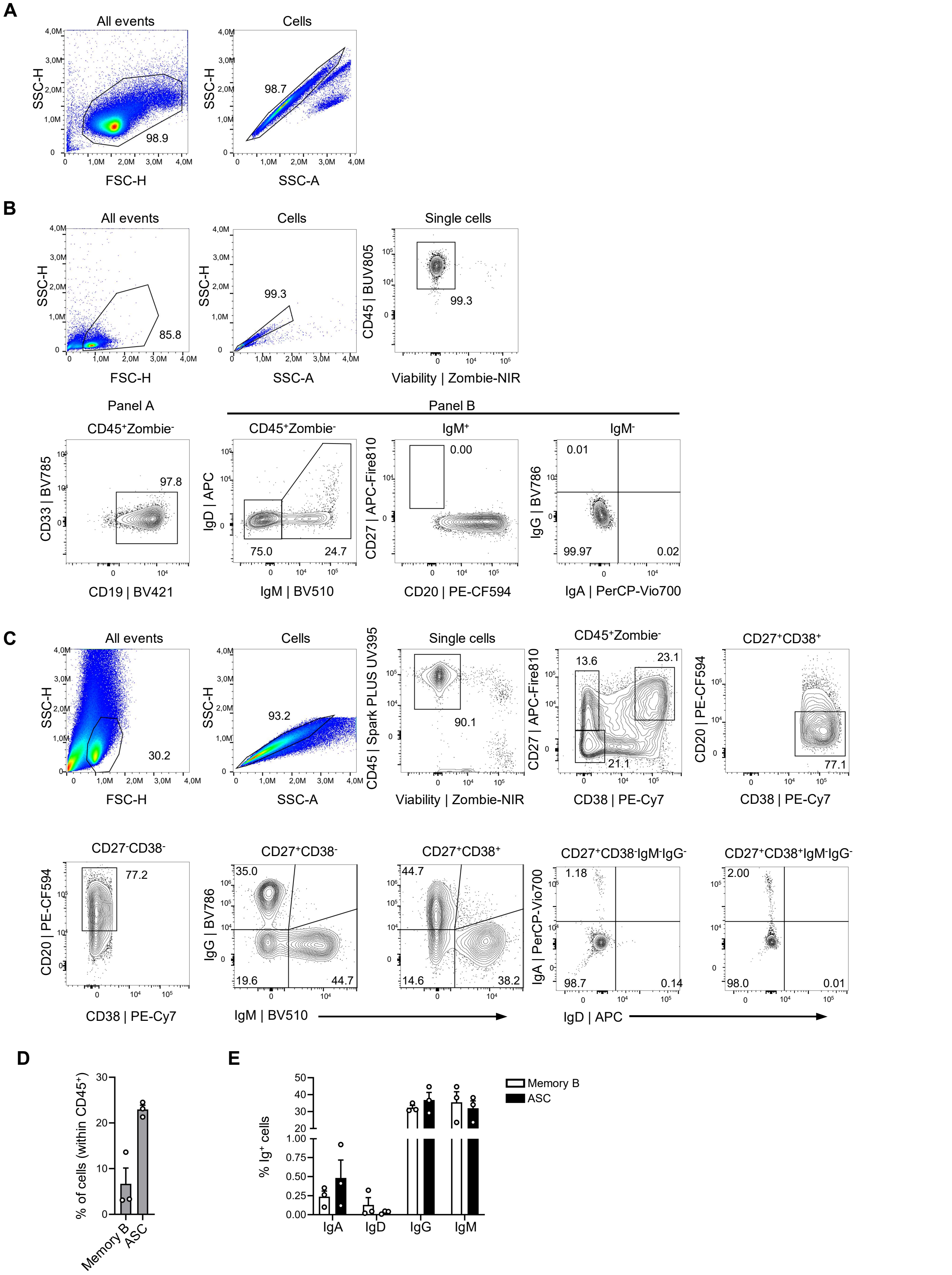

Figure S5. Functional assessment and gating strategies of activated ABO-CD19$^+$ B cells. Related to Figure 5.
(**A, B**) Gating strategies prior to determining Ca^{2+} flux (**A**) in day 35 ABO-CD19$^+$ cells. (**B**) Gating strategy used for assessing purity (panel A) and maturation state (panel B) of CD19$^+$ cells isolated from day 35 ABOs.
(**C**) Gating strategy used for the flow cytometric analysis of antibody-secreting cells (ASCs) at day 11 post-stimulation of ABO-CD19$^+$ cells using UCB-derived HSPCs.
(**D**) Quantification of the frequencies of CD27$^+$CD38$^-$ memory B cells and CD27$^+$CD38^{hi}
(**E**) Distribution of IgM$^+$ and Ig class-switched memory B cells and ASCs at day 11 post-stimulation of ABO-CD19$^+$ cells using UCB-derived HSPCs.
(**D, E**) Data are presented as mean ± SEM and represent n=3 individual donors in independent cultures.

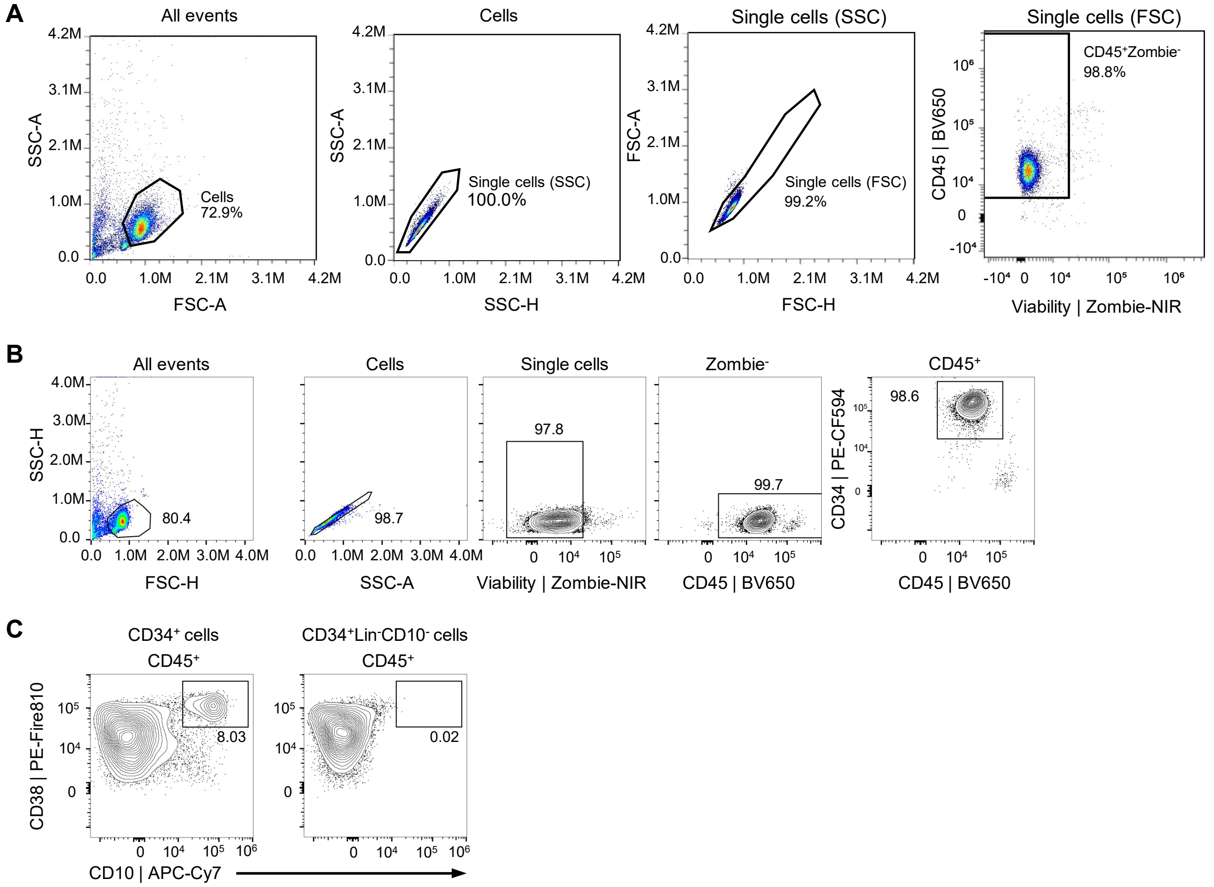

Figure S6. Depletion of B-cell progenitors does not influence B-cell development in ABOs. Related to Figure 6.
(**A**) Gating strategy used prior to opt-SNE analysis.
(**B**) Gating strategy used for quantification of $CD10^+$ cells depletion.
(**C**) Representative flow cytometric analysis of CD10 expression on HSPCs before (left) and after depletion of Lin^+CD10^+ cells (right) by MACS purification. Data represents n=3 individual donors in independent cultures.

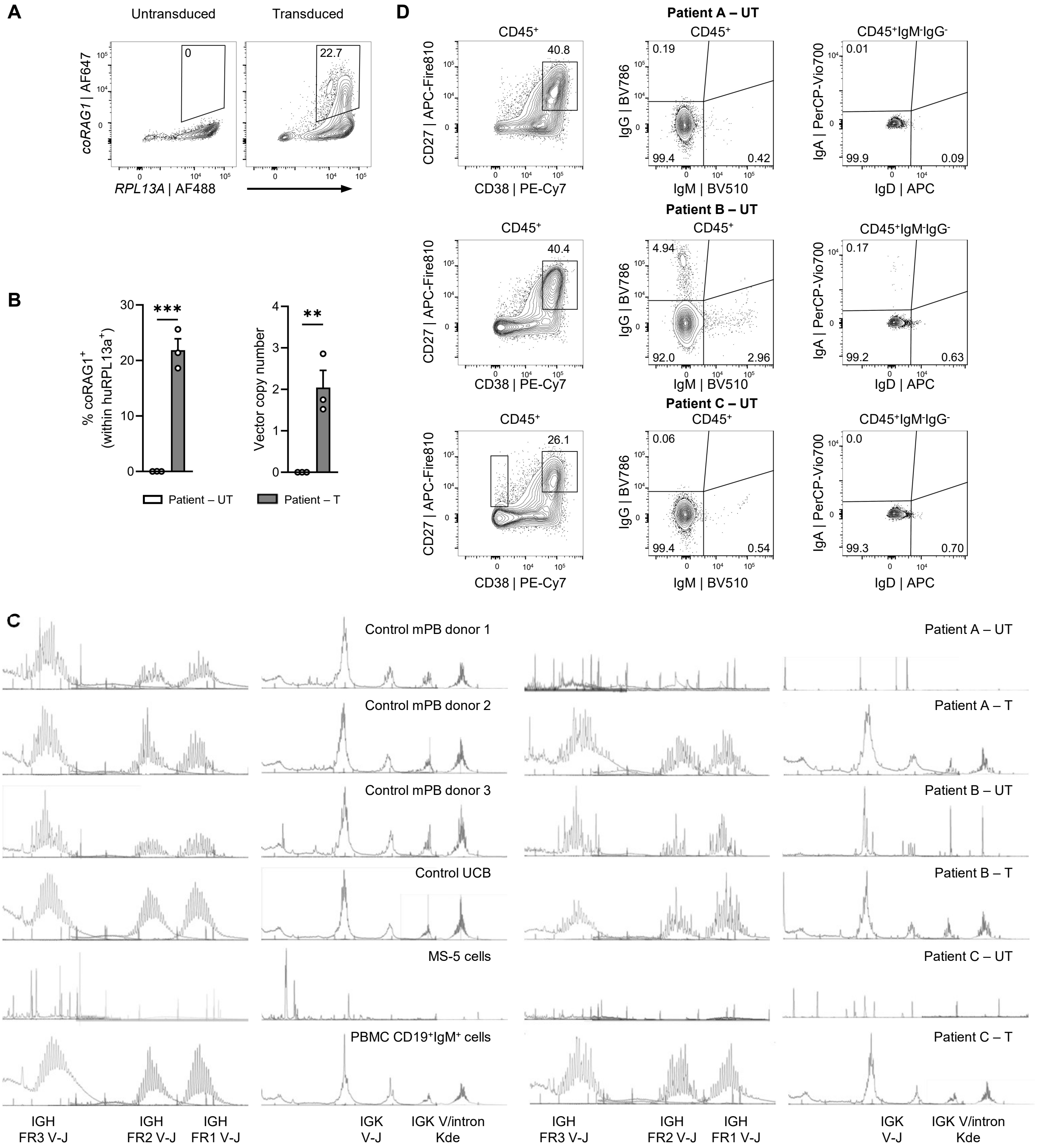

Figure S7. B-cell development from healthy control or RAG1-deficient mPB-derived HSPCs ABOs. Related to Figure 7.
(**A**) Representative flow cytometric analysis showing coRAG1 transduction efficiency (RPL13A; housekeeping gene) assessed by the PrimeFlow assay in RAG1-deficient mPB-derived HSPCs.
(**B**) Quantification of the frequency of coRAG1$^+$ (left) and corresponding vector copy numbers (right) in 9-day cultured RAG1-deficient mPB-derived HSPCs.
(**A**, **B**) Data are presented as mean ± SEM and represent n=3 from individual donors in independent cultures. Statistical analysis was performed using an unpaired Students *t*-test (**p<0.01, ***p<0.001).
(**C**) Visualization of V_H-J_H rearrangements across the conserved framework regions (FR1-3) of the *IGHV* locus and V_K-J_K rearrangements in the *IGKV* locus including V/intron Kappa deletion elements (Kde) by GeneScan analysis from healthy control, untransduced (UT), and coRAG1 transduced (T) RAG1-deficient mPB HSPCs-derived ABOs harvested on day 42, UCB HSPCs-derived ABOs (representative of n=4) harvested on day 35. (Negative control: MS-5 cells; positive control: PBMC-derived CD19$^+$IgM$^+$ cells.
(**D**) Flow cytometric analysis showing the presence CD27$^+$CD38$^+$ ASCs upon CD40 stimulation of ABO-CD19$^+$ cells for 11 days.

Patient with RAG1-SCID	Age	Sex	Mutation coding DNA	Protein sequence change	Zygosity	Clinical phenotype	
						T cells	B cells
Pt. A	4 mo.	M	c.[519del];[519del]	p.[(Glu174fs)]; [(Glu174fs)]	Homozygous	severely decreased	severely decreased
Pt. B	5 mo.	M	c.256_257del	p.(Lys86Valfs*33)	Homozygous	severely decreased	decreased
Pt. C	8 mo.	F	c.2096G>A	p.Arg699Gln	Homozygous	undetectable	severely decreased

Table S1. RAG1 mutations in HSPCs from patients with RAG1-SCID used in the study. Related to Figure 7. Mo., months; M, male; F, female.

Supplemental references

S1. Zeng, A.G.X., Iacobucci, I., Shah, S., Mitchell, A., Wong, G., Bansal, S., Chen, D., Gao, Q., Kim, H., Kennedy, J.A., et al. (2025). Single-cell Transcriptional Atlas of Human Hematopoiesis Reveals Genetic and Hierarchy-Based Determinants of Aberrant AML Differentiation. Blood Cancer Discov 6, 307– 324. 10.1158/2643-3230.BCD-24-0342.
