# Supplementary material for: ABO: A 3D stroma-supported culture platform enabling full human B-lymphopoiesis for disease modeling and gene therapy development
Source: Cell Rep Med. 2026 Jun 18;7(7):102879. doi: 10.1016/j.xcrm.2026.102879 (PMC13400188; doi:10.1016/j.xcrm.2026.102879)
Supplement: Document S2. Article plus supplemental information [file mmc2.pdf]

Cell Reports Medicine

Article

ABO: A 3D stroma-supported culture platform enabling full human B-lymphopoiesis for disease modeling and gene therapy development

Graphical abstract

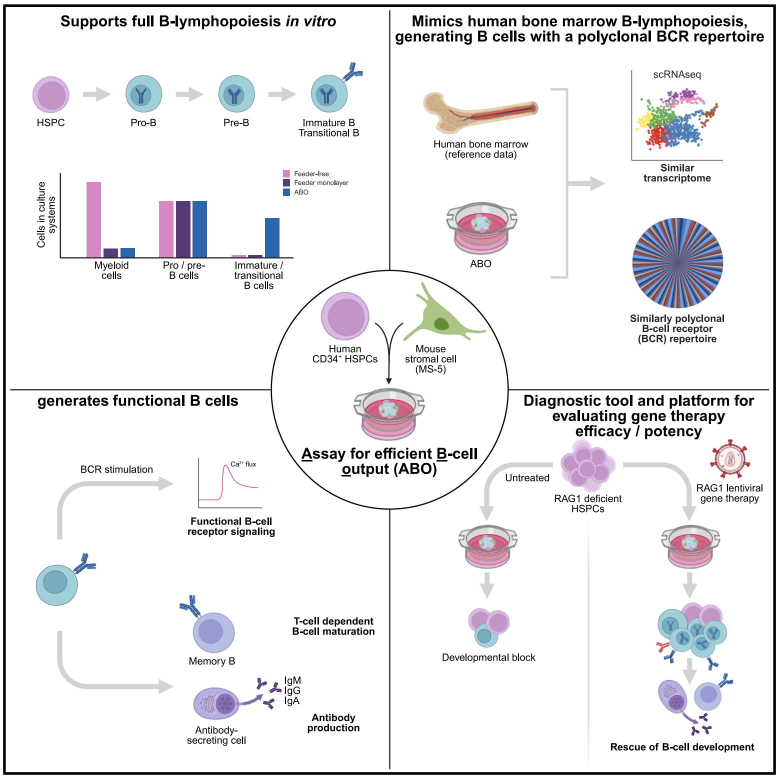

Highlights

- 3D aggregate cocultures (ABOs) enable full human B cell development *in vitro*
- ABOs generate B cells that can mature into class-switched antibody-secreting cells
- ABOs recapitulate the B cell developmental arrest of RAG1-deficient HSPCs
- ABOs display the rescue of B cell development upon lentiviral *RAG1* gene correction

Authors

Merijn Braams, Martijn Cordes, Sandra A. Vloemans, ..., Frank J.T. Staal, Kirsten Canté-Barrett, Sander de Kivit

Correspondence

f.j.t.staal@lumc.nl (F.J.T.S.),
k.cante@lumc.nl (K.C.-B.),
s.de_kivit@lumc.nl (S.d.K.)

In brief

Braams et al. establish 3D aggregate cocultures (ABOs) generating functional B cells from hematopoietic stem and progenitor cells (HSPCs). ABOs recapitulate the B cell developmental arrest of RAG1-deficient HSPCs, which is rescued by *RAG1* gene correction, and thus provide a valuable tool to study B cell immunodeficiencies and test gene therapy strategies.

Braams et al., 2026, Cell Reports Medicine 7, 102879
July 21, 2026 © 2026 The Author(s). Published by Elsevier Inc.
https://doi.org/10.1016/j.xcrm.2026.102879

CellPress

Cell Reports Medicine

CellPress
OPEN ACCESS

Article

ABO: A 3D stroma-supported culture platform enabling full human B-lymphopoiesis for disease modeling and gene therapy development

Merijn Braams,[1,2] Martijn Cordes,[1,3] Sandra A. Vloemans,[1] Bas de Mooij,[1] Sandra de Bruin-Versteeg,[1] Ashley Wachtmeester,[1] Anton W. Langerak,[4] Karin Pike-Overzet,[1] Frank J.T. Staal,[1,2,3,5,*] Kirsten Canté-Barrett,[1,2,5,*] and Sander de Kivit[1,2,5,6,*]

[1]Department of Immunology, Leiden University Medical Center, 2300RC Leiden, the Netherlands
[2]Novo Nordisk Foundation Center for Stem Cell Medicine, reNEW, Leiden University Medical Center, Leiden, the Netherlands
[3]Department of Pediatrics, Leiden University Medical Center, 2300RC Leiden, the Netherlands
[4]Department of Immunology, Laboratory Medical Immunology, Erasmus Medical Center, University Medical Center Rotterdam, 3015GD Rotterdam, the Netherlands
[5]Senior author
[6]Lead contact
*Correspondence: f.j.t.staal@lumc.nl (F.J.T.S.), k.cante@lumc.nl (K.C.-B.), s.de_kivit@lumc.nl (S.d.K.)
https://doi.org/10.1016/j.xcrm.2026.102879

SUMMARY

Defective B cell development underlies a large proportion of inborn errors of immunity. Progress in understanding disease mechanisms and therapy development remains limited because current human *in vitro* models incompletely recapitulate B-lymphopoiesis. We present a three-dimensional aggregate culture platform composed of human hematopoietic stem and progenitor cells (HSPCs) and mouse bone marrow (BM) stromal cells as an assay for efficient B cell output (ABO). ABOs support progression through pro-B, pre-B, immature, and transitional B cell stages, with a transcriptional profile and B cell receptor (BCR) repertoire diversity resembling human B cell ontogeny. ABO-derived B cells exhibit functional BCR signaling and differentiate into class-switched memory B cells and antibody-secreting cells following CD40-mediated stimulation. ABOs reproduce the B cell developmental arrest observed in HSPCs from patients with RAG1-deficient severe combined immunodeficiency (SCID), which is corrected by a RAG1 gene therapy lentiviral vector. Thus, ABOs provide a clinically relevant platform for modeling B cell immunodeficiencies and evaluating therapeutic strategies.

INTRODUCTION

B cells are central to humoral immunity, producing antigen-specific antibodies that neutralize pathogens and maintain immune homeostasis. Hematopoietic stem and progenitor cells (HSPCs) reside in specialized niches in the bone marrow (BM) and receive stromal-cell-derived signals to commit to the B-cell lineage.[1,2] The first developmental stages of B-lymphopoiesis critically depend on interleukin (IL)-7, which promotes the expression of lineage-defining transcription factors such as EBF1 and PAX5. These factors induce RAG1 and RAG2 expression to facilitate V(D)J recombination and (pre-)B cell receptor (BCR) assembly during the pro-B and pre-B cell stages.[3,4] Genetic disruptions in these processes underlie a spectrum of inborn errors of immunity (IEIs), comprising more than 450 rare diseases caused by mutations in genes essential for the development of the immune system.[5] Some IEIs lead to complete absence of functional B cells, as observed in X-linked agammaglobulinemia (XLA)[6,7] or RAG1-deficient severe combined immunodeficiency (SCID).[8] In less severe forms of B cell immunodeficiencies, such as common variable immunodeficiency (CVID), the underlying B cell developmental defects remain poorly understood, largely because available *in vitro* systems do not fully recapitulate B cell development from HSPCs to mature B cells.

For disorders such as XLA, Wiskott-Aldrich syndrome, and SCIDs, transplantation of corrected autologous HSPCs, following *ex vivo* gene therapy using gene addition or precise gene-editing approaches, offers a promising strategy to restore impaired B cell development.[9] Translation of gene therapies from concept to clinic requires robust preclinical validation. For B cell immunodeficiencies, this depends on reliable *in vitro* B-lymphopoiesis systems that enable rapid and reproducible assessment of whether genetically corrected HSPCs develop into functional B cells. Current *in vitro* models promote development of HSPCs into B cells through stimulation with factors such as FMS-like tyrosine kinase 3 ligand (FLT3L), stem cell factor (SCF), interleukin (IL)-6, and IL-7, either in stroma-free conditions or in coculture with murine BM-derived stromal cell lines.[10–13] Although current *in vitro* models can recapitulate early

© 2026 The Author(s). Published by Elsevier Inc.
This is an open access article under the CC BY license (http://creativecommons.org/licenses/by/4.0/).

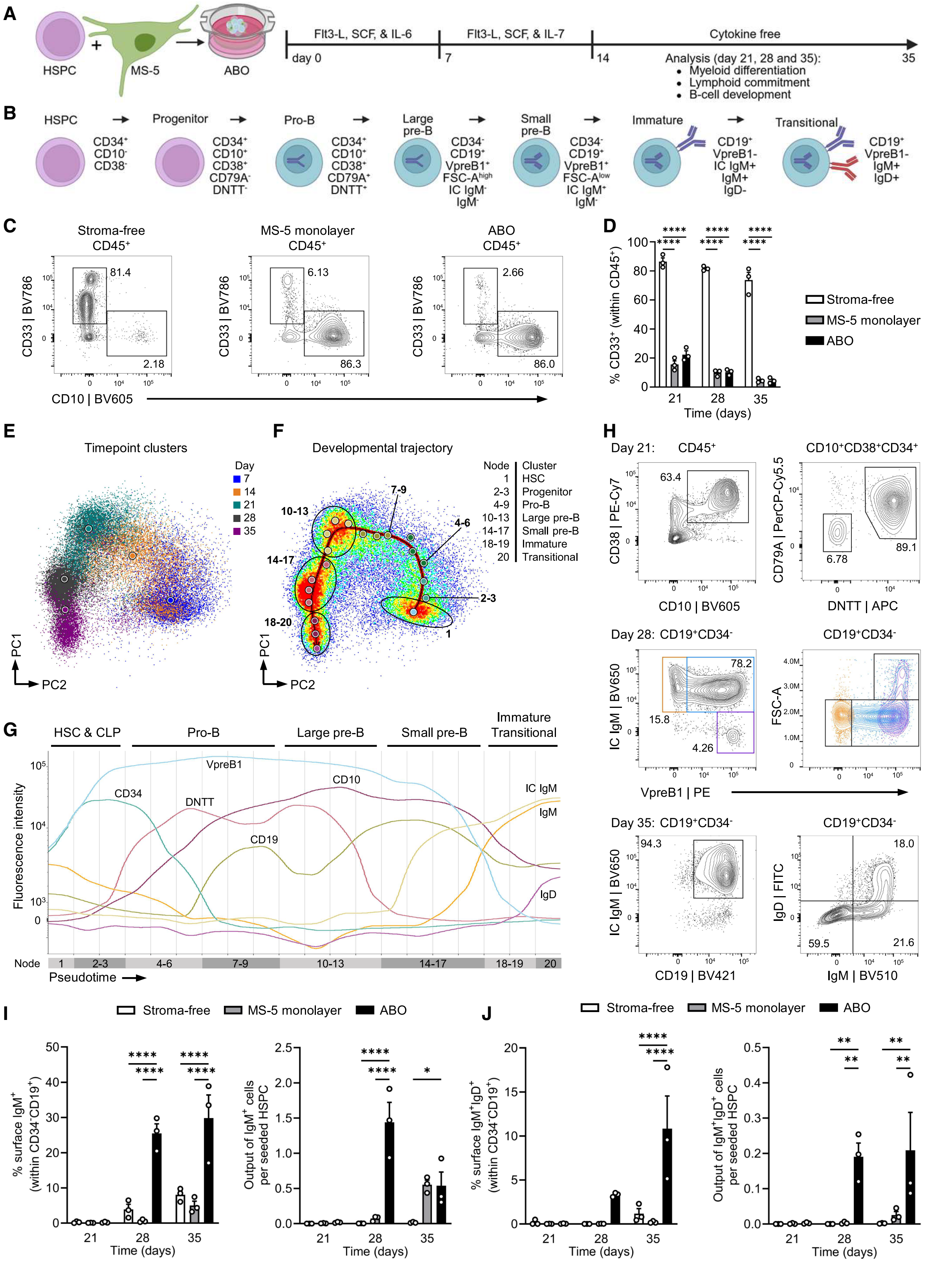

(legend on next page)

developmental blocks in B-lymphopoiesis,[4,14–16] they often skew differentiation toward the myeloid lineage and typically arrest at the pre-B cell stage. This limits B cell output and functional evaluation of B cells derived from such cultures. Hence, there is an urgent need for *in vitro* platforms that more accurately model human B cell development and support both diagnostic assessment and functional testing of gene therapy strategies.

T cell development can be mimicked in artificial thymic organoids (ATOs)—three-dimensional (3D) assemblies of HSPCs with murine BM-derived stromal cells expressing the Notch ligand Delta-like (DLL) 1 or −4.[17] ATOs provide a valuable platform to investigate T cell development in the context of IEIs[18–21] and to functionally characterize yet unknown genetic causes of SCID.[22] Monolayer cocultures using OP9 BM stromal cells expressing DLL1 support T cell development from human CD34$^+$ cells.[23] ATOs, however, employ MS-5 BM stromal cells transduced with human DLL1, which improves the generation of mature and functional CD8$^+$ T cells.[17] Historically, MS-5 cells have been used in monolayer cultures to drive B cell development from HSPCs.[10,24–27] Though this culture method, using different HSPC sources,[26,28] generally yields sufficient numbers of CD19$^+$ cells, the frequencies of fully developed IgM$^+$IgD$^-$ immature and IgM$^+$IgD$^+$ transitional B cells remain low or absent or have not been thoroughly characterized.

We established a 3D ATO-based coculture platform combining HSPCs and BM stromal cells as an assay for efficient B cell output (the ABO platform), which fully recapitulates *de novo* B-lymphopoiesis from human HSPCs. Its qualitative performance mirrors B cell development following xenotransplantation of HSPCs into highly immunodeficient NOD/scid/IL2rγ^{null} (NSG) mice. We used single-cell transcriptomic analyses to confirm that ABO-derived B cells (ABO-B cells) resemble their human BM counterparts both phenotypically and transcriptionally and that they exhibit a polyclonal BCR repertoire. We also show that ABO-B cells respond to BCR stimulation, can further mature into class-switched antibody-secreting cells (ASCs), and that ABOs can serve as a preclinical platform for gene therapy development by demonstrating rescue of B-lymphopoiesis in HSPCs derived from patients with RAG1-SCID using our clinically validated codon-optimized (co)RAG1 lentiviral (LV) vector.

RESULTS

ABOs support efficient B cell development from HSPCs

HSPCs isolated from umbilical cord blood (UCB) were combined with MS-5 BM stromal cells to assemble ABOs. In the first week of culture, ABOs were supplemented with FLT3L and SCF to promote the survival and proliferation of early HSPCs[29] and the expansion of lymphoid-primed progenitors,[30] while IL-6 supports proliferation and maintenance of early hematopoietic progenitors.[31] To reduce myeloid bias, IL-6 was removed after the first week of culture and replaced by IL-7 to further instruct B-lineage commitment.[4,32,33] ABOs were subsequently maintained without cytokines for an additional 21 days (Figure 1A). B cell development (Figure 1B) was evaluated and benchmarked against stroma-free[11–13] and MS-5/HSPC (2D monolayer) coculture systems.[10,24–27]

We first assessed B-lineage commitment of HSPCs in ABOs. While stroma-free cultures using HSPCs seeded on ICAM1-Fc-coated plates[12] showed a pronounced bias toward CD33$^+$ myeloid cell differentiation, coculture of HSPCs with MS-5 cells—in either ABO or monolayer configuration—predominantly generated CD10$^+$ lymphoid-committed cells (Figures 1C and 1D), confirming the requirement of stromal support for B-lineage specification.[14,30]

Next, we applied an unbiased computational approach based on flow cytometric analysis of stage-specific marker expression to characterize B cell development in ABOs. Principal-component analysis (PCA) revealed five distinct clusters corresponding to different developmental stages (Figure 1E). In subsequent trajectory analysis, we defined 20 developmental nodes based on the expression patterns of key lineage markers, enabling the mapping of these nodes to discrete stages of B cell maturation (Figures 1F and 1G). On days 14–21, ABOs contained CD10$^+$CD38$^+$ cells expressing immunoglobulin alpha (Igα) (CD79A) and terminal deoxynucleotidyl transferase (DNTT; also known as TdT), characteristic of pro-B cells. These cells progressed to CD19$^+$ pre-B cells that gradually lost VpreB1 and acquired intracellular IgM. By day 35, only ABO cultures efficiently generated immature B cells expressing surface IgM and transitional B cells coexpressing surface IgM and IgD (Figures 1H, S1A, and S1B). These findings demonstrate that ABOs support human B-lymphopoiesis, generating both IgM$^+$IgD$^-$ immature and IgM$^+$IgD$^+$ transitional B cells.

Finally, we compared the B cell output per HSPC among stroma-free, MS-5 monolayer, and ABO cultures. Stroma-free and MS-5 monolayer systems predominantly stalled at the pre-B cell stage (Figure S1C). While both MS-5-based systems supported early B-lineage commitment (Figure 1C), ABOs consistently yielded a higher proportion of immature and transitional B cells and, concomitantly, an increased output of IgM$^+$IgD$^-$ and IgM$^+$IgD$^+$ B cells per CD34$^+$ HSPC, relative to stroma-free and MS-5 monolayer cultures (Figures 1I, 1J, and S1D–S1H). Collectively, these results demonstrate that the

Figure 1. ABOs support efficient B cell development from HSPCs

(A) Schematic overview of the ABO culture system.

(B) Schematic overview of B cell development from HSPCs. (A and B) Created using BioRender (BioRender.com/9tddlci).

(C) Flow cytometric plots showing CD33$^+$ myeloid and CD10$^+$ lymphoid populations at day 35.

(D) Quantification of CD33$^+$ cell frequencies at days 21–35.

(E and F) PCA illustrating the developmental trajectory of HSPCs in ABOs over time (E) and annotated with corresponding B cell stages (F).

(G) Wanderlust trajectory depicting the dynamic expression of key markers during B cell development. (CLP, common lymphoid progenitors; IC, intracellular).

(H) Two-dimensional flow cytometric plots showing marker expression along the Wanderlust trajectory.

(I and J) Quantification of IgM$^+$ immature (I) and IgM$^+$IgD$^+$ transitional B cells (J), shown as frequencies and output per input HSPC at days 21–35. (D, I, J) Statistical analysis was performed using two-way ANOVA with Tukey's multiple-comparison test (*$p < 0.05$, **$p < 0.01$, ****$p < 0.0001$). Data are presented as mean ± SEM. (C–J) Results represent $n = 3$ individual donors in independent cultures. See also Figure S1.

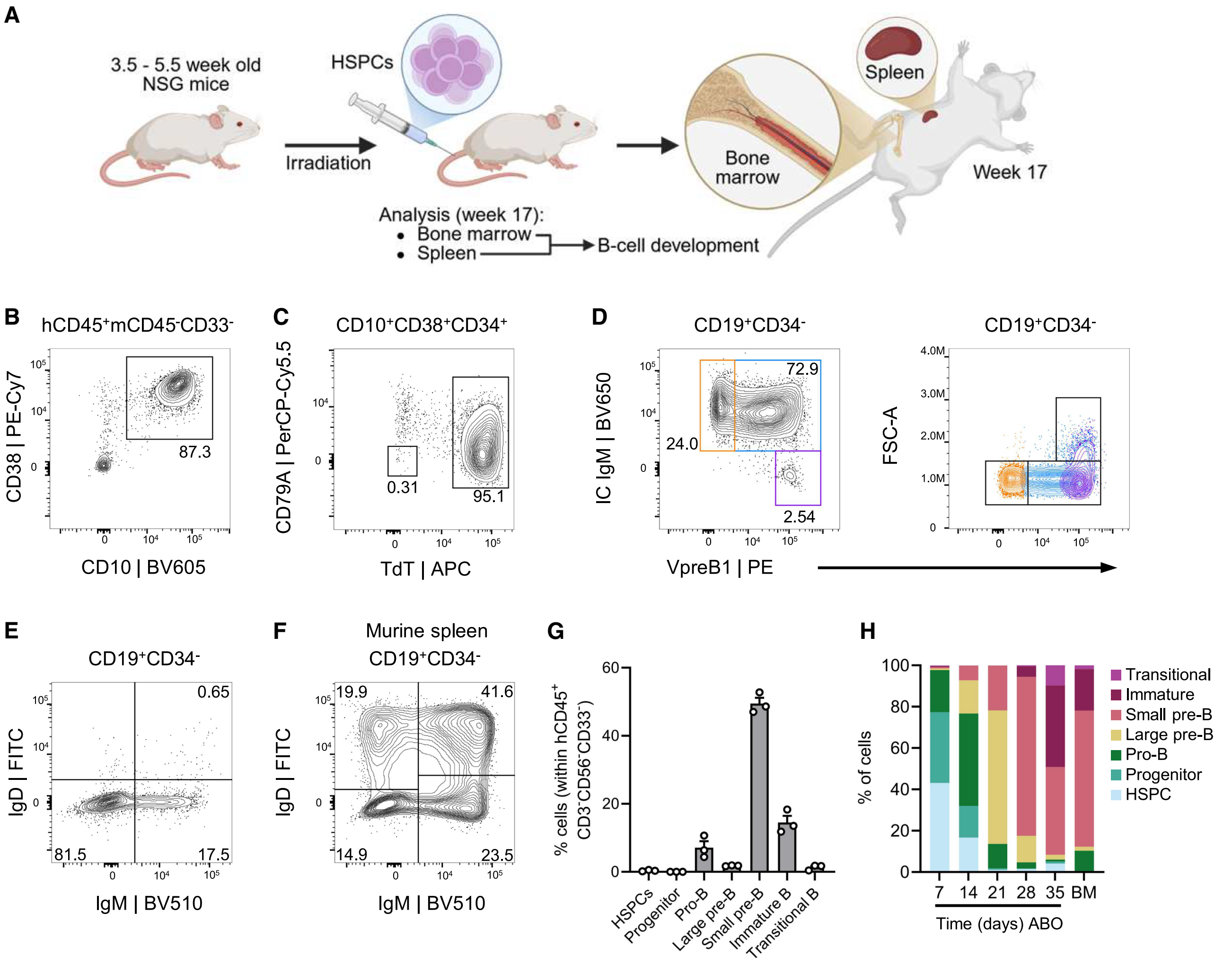

Figure 2. B cell development in ABOs resembles that in xenotransplanted NSG mice

(A) Schematic overview of UCB-derived HSPCs transplantation into irradiated NSG mice. Created using BioRender (BioRender.com/vxdxbc4).

(B–F) Flow cytometric analysis of human CD45$^+$ cells in BM (C–E) and spleen (F). (IC, intracellular).

(G) Proportions of B cell developmental stages in the BM of human HSPCs transplanted in NSG mice. Data are presented as mean ± SEM. (H) Comparison of B cell developmental stages in ABOs over time with week 17 mouse BM. (B–H) Data represent n = 3 independent biological replicates. See also Figure S2.

ABOs robustly support stepwise human B cell development from HSPCs and is superior to existing models in producing more mature B-lymphocyte subsets.

B cell development in ABOs resembles that in xenotransplanted NSG mice

Preclinical immunological assays often warrant confirmation in an *in vivo* system. We therefore employed a humanized xenograft transplantation model using human UCB-derived HSPCs in NSG mice and compared B-lymphopoiesis observed in ABOs with *in vivo* B cell development in the BM of these mice. At 17 weeks post-transplantation, BM and spleens were harvested and analyzed for B cell development (Figures 2A and S2). The distribution of B-cell developmental stages in murine BM resembled that of day 28–35 ABOs (Figures 2B–2H). Transitional B cells detected in day 35 ABOs were absent from the BM (Figure 2E), but present in the spleen (Figure 2F), consistent with their physiological migration to peripheral lymphoid tissues. Together, these findings demonstrate that ABOs mimic human B cell development as observed *in vivo*.

Single-cell transcriptome analysis reveals similar B cell developmental trajectories in ABOs and healthy human BM

To investigate whether B cells developing in ABOs recapitulate the transcriptional programs of human B cell ontogeny, we performed single-cell RNA sequencing (scRNA-seq) on CD10$^+$CD38$^+$ cells (Figure S3A) isolated from day 21, 28, and 35 UCB-derived ABOs and mapped them onto a reference dataset of healthy human BM[34] (Figures S3B–S3D). This confirmed the presence of different developmental B cell subsets in ABOs, representing all stages of B cell developmental present

CellPress
OPEN ACCESS

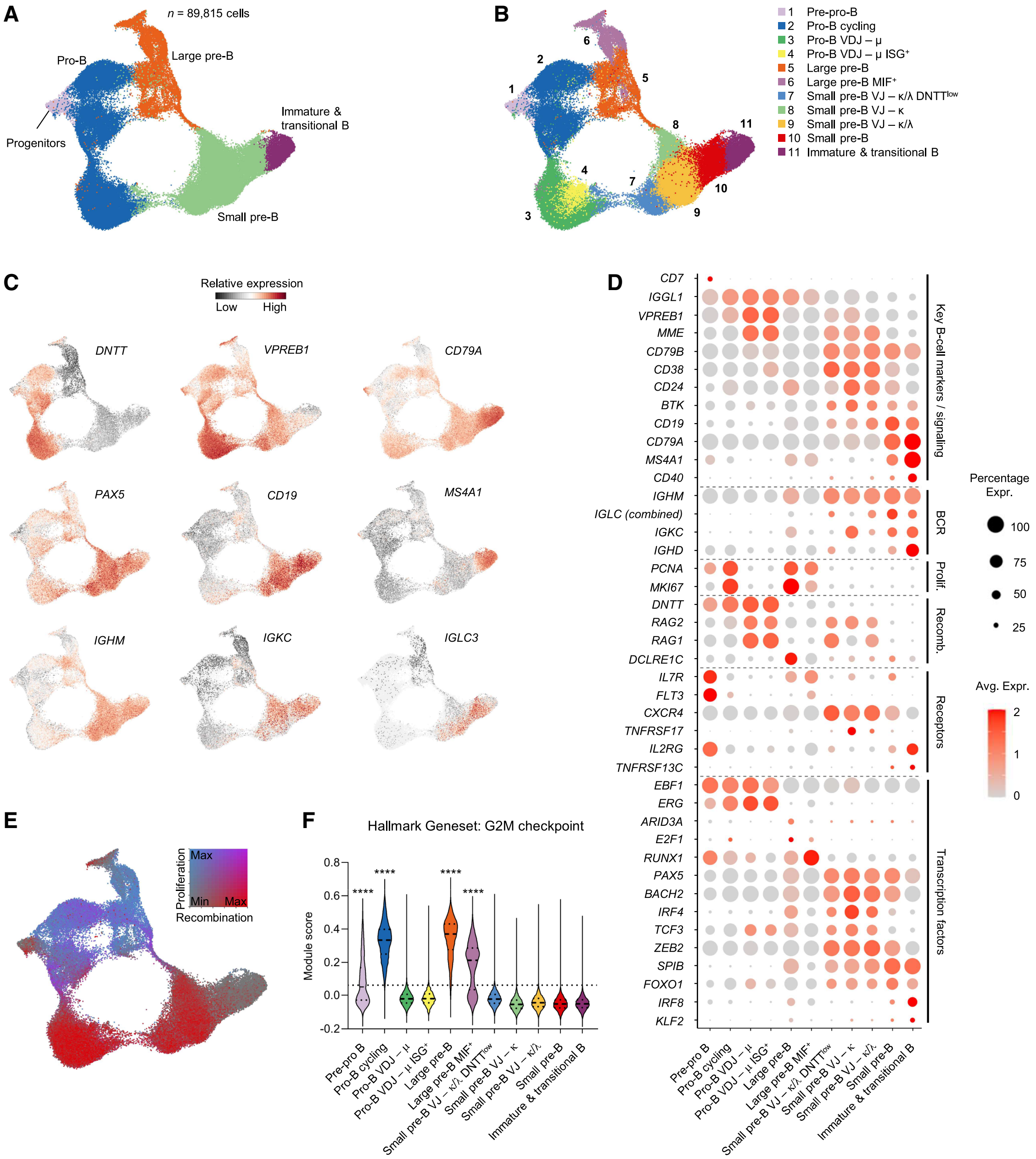

Figure 3. Single-cell transcriptome analysis reveals similar B cell developmental trajectories in ABOs and healthy human BM

(A and B) Projections of clusters annotated using healthy BM datasets[4,34] onto the UMAP derived from scRNA-seq data of day 21–35 ABOs.

(C) Gene expression levels of key transcription factors and B cell markers projected on the UMAP generated from day 21–35 ABOs. Relative expression levels are color coded.

(D) Dot plot depicting stage-specific expression of genes during B cell development. Genes are hierarchically ordered within functional categories. (Prolif., proliferation; Recomb., recombination). (E) Projection of module scores for proliferation and recombination, calculated based on *RAG1*/*RAG2* expression, onto the UMAP derived from day 21–35 ABOs.

(F) Violin plots illustrating proliferation activity within each annotated cluster, as determined by module scoring. Statistical analysis was performed using a one-sample *t* test (****$p < 0.0001$, dashed line: average module score of the dataset). (A–F) Data represent $n = 4$ individual donors in independent cultures. See also Figure S3.

in healthy human BM. UMAP visualization and clustering of the scRNA-seq dataset from ABOs revealed 11 clusters corresponding to distinct stages of B cell development (Figures 3A and 3B), with all donors represented in each cluster (Figure S3E).

Temporal changes in the transcriptional program were observed in ABOs (Figure S3F). Day 21 cultures were enriched for clusters 1–4, corresponding to progenitors (*CD7*, *RUNX1*, *FLT3*, and *IL7R*) and pro-B cells (*EBF1*, *DNTT*, *VPREB1*,

RAG1/2, *DCLRE1C*, and *MME*), reflecting early B-lineage specification and initiation of V(D)J recombination. Clusters 5–10 emerged in day 28 cultures, representing pre-B cells expressing transcription factors that drive B-lineage commitment (*RUNX1*, *BACH2*, *PAX5*, *ZEB2*, and *SPIB*), along with a second wave of recombination (*RAG1/2*), BCR components and markers (*CD19*, *CD24*, *CD79A/B*, *BTK*), and immunoglobulin (Ig) chains (*IGHM*, *IGKC*, and *IGLC*) alongside gradual loss of *VPREB1*, consistent with progression of light chain rearrangement. Specifically, *TNFRSF17* was identified in pre-B cells as well, suggesting a potential role for TNFRSF17 in early B cell development. By day 35, cluster 11 appeared, comprising immature and transitional B cells marked by surface and signaling molecules (*CD40*, *MS4A1*, and *TNFRSF13C*), Igs (*IGHM* and *IGHD*), and transcription factors regulating B cell maturation (*IRF8* and *KLF2*) (Figures 3C and 3D).

To assess functional activity across clusters, we calculated module scores reflecting proliferation and recombination, based on the hallmark G2M checkpoint gene set and *RAG1/2* expression, respectively, and mapped these scores on the UMAP of our scRNA-seq dataset (Figures 3A and 3B). Proliferation and recombination activity were mutually exclusive (Figure 3E), with high recombination activity restricted to clusters 3, 4, 7, and 9, corresponding to pro-B cells and small pre-B cells (Figures S3G and S3H). High proliferative activity was detected in clusters 2, 5, and 6, corresponding to cycling pro-B and large pre-B cells (Figure 3F), which have successfully completed Ig heavy-chain rearrangement, express *IL7R* (Figure 3D), and undergo a pre-BCR-driven proliferative burst. These data show that the ABOs reflect normal stage-specific regulation of cell cycling and V(D)J recombination during human B cell development.

ABO-B cells exhibit a diverse BCR repertoire

Having established that ABOs support complete BM B cell development up to the transitional B cell stage, we next examined the BCR repertoire diversity in these cells. In all donors, immature and transitional B cells (cluster 11) exhibited extensive clonal diversity, as evidenced by a high frequency of unique clones—comparable to healthy BM—and minimal clonal overlap between donors (Figures 4A–4C). Analysis of the CDR3 region, a key determinant of antigen receptor diversity, revealed near-normal, bell-shaped CDR3 length distributions for both Ig heavy and light chains (Figures 4D and 4E). The distributions peaked at 15–16 amino acids for heavy chains and 13 amino acids for light chains, closely mirroring those observed in human *in vivo* naive BCR repertoires.[35–37] Thus, B cell development in ABOs is polyclonal, donor-specific, and driven by stochastic V(D)J recombination, mirroring the natural diversity of the human B cell repertoire.

Next, we analyzed V- and J-gene segment usage during Ig heavy- and light-chain rearrangements. Consistent with normal B cell development, analysis of Ig heavy variable (*IGHV*) gene usage in ABO-derived immature and transitional B cells revealed a broad distribution of V- and J-gene segment usage that closely resembles that of human naive B cells *in vivo*.[37] The most prominent usage involved *IGHV3-23*, *IGHV3-33*, and *IGHV4-34*, each constituting ∼5% usage within the total repertoire, with V gene segments most frequently joined to *IGHJ4* (Figures 4F and S4A). Similarly, Igκ and Igλ (*IGKV* and *IGLV*) variable genes followed reported Gaussian patterns.[38,39] Rearrangements at the Igκ locus predominantly occurred using *IGKV1-33*, *IGKV2-30*, *IGKV3-20*, and *IGKV4-1* joined to *IGKJ2*, *-3*, and *-4*, while Igλ rearrangements were most frequently observed at *IGLV1-44*, *IGLV1-47*, and *IGLV2-14* gene segments, primarily joined to *IGLJ1*, *-2*, and *-3* (Figures 4G, S4B, and S4C). This repertoire profile indicates no apparent skewing in Ig heavy- and light-chain rearrangements, further supporting that ABOs generate a highly diverse and normally distributed BCR repertoire.

ABO-B cells express a functional BCR and can mature into class-switched ASCs

Since ABOs yield increased frequencies of surface IgM^{+} B cells (Figures 1H–1J), we next examined whether these cells express a functional BCR. To this end, CD19^{+} cells were isolated from day 35 UCB-derived ABOs (ABO-CD19^{+} cells) and subjected to Ca^{2+} flux analysis following IgM crosslinking (Figure S5A). BCR stimulation resulted in a reproducible, rapid increase in intracellular Ca^{2+} levels (Figure 5A), indicating that B cells generated in ABOs harbor functional BCR signaling and suggesting that these cells are capable of responding to antigens.

In addition to BCR-mediated antigen recognition, B cells undergo class-switch recombination (CSR) and somatic hypermutation (SHM) during germinal center responses, following interactions with follicular helper T cells that provide CD40 co-stimulation and secrete cytokines such as IL-4 and IL-21.[40] We first assessed the maturation status of day 35 ABO-CD19^{+} cells. Cluster 11 cells (Figure 3B)—annotated as immature and transitional B cells—exhibited high expression of *CD38*, *MS4A1* (encoding CD20), *PAX5*, *IGHM*, and *IGHD* transcripts, while lacking transcripts characteristic of memory B cell and ASCs, including *CD27* and the transcription factor *PRDM1*[40] (encoding BLIMP1, a key transcription factor regulating plasma cell differentiation[41,42]) (Figure 5B). These cells also lacked class-switched Ig transcripts (*IGHG1*, *IGHG3*, *IGHG4*, *IGHA1, IGHA2*, or *IGHE*) (Figure 5B). Flow cytometry showed that day 35 ABO-B cells were CD33^{-}CD19^{+}CD20^{+}CD27^{-} and did not express class-switched Ig isotypes at the protein level (Figures 5C and S5B), confirming an immature B cell phenotype.

To determine whether ABO-CD19^{+} cells can undergo CSR and SHM and mature into plasma cells in response to CD40 co-stimulation, we reaggregated these cells in new ABOs with MS-5 stromal cells expressing human CD40L for 11 days (Figure 5D). CD27^{+}CD38^{-} memory B cells and CD27^{hi}CD38^{+}CD20^{-} ASCs were detected on day 11 post-stimulation (Figures 5E and S5C–S5E). Plasmablast-to-plasma cell differentiation was apparent by a marked increase in *PRDM1* expression on day 11 (Figure 5F), which promotes plasma cell identity in part through repression of transcription factors, such as PAX5, that are required for B-cell identity.[42,43] Consistently, PAX5 mRNA (Figure 5G) and protein expression (Figure 5H) progressively decreased following CD40 ligation, indicating that ABO-B cells can mature into antibody-secreting plasma cells.

Both CSR and SHM require activation-induced cytidine deaminase (AID) activity, encoded by *AICDA*, which is induced upon CD40 co-stimulation.[44] While unstimulated ABO-CD19^{+} cells had low *AICDA* expression, this was increased upon CD40

CellPress
OPEN ACCESS

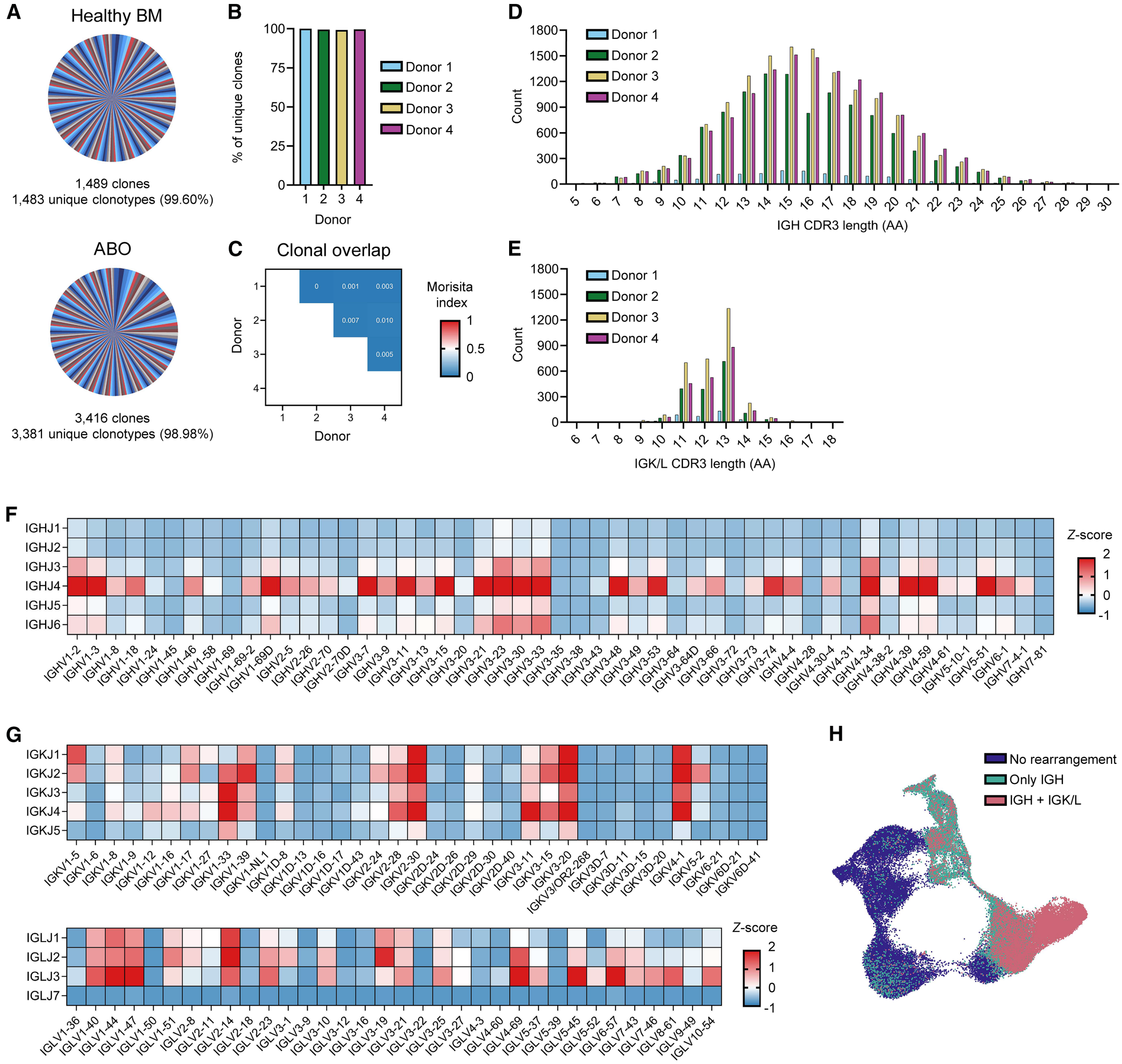

Figure 4. ABO-B cells exhibit a diverse BCR repertoire

(A) Pie charts showing BCR clonotype diversity (top 200 clones shown) of immature and transitional B cells detected in healthy BM[34] and ABOs. Frequencies of unique clonotypes are shown.

(B) Quantification of the percentage of unique BCR clonotypes within immature and transitional B cells in ABOs, separated by individual donors.

(C) Comparison of clonal overlap (Morisita index) between donors of immature and transitional B cells in ABOs.

(D and E) Analysis of the distribution of CDR3 amino acid (AA) length within the Ig heavy (*IGH*) (D) and light (*IGK/L*) chains (E) of immature and transitional B cells in ABOs, separated by the individual donors.

(F and G) Heatmaps showing V- and J-gene segment usage generating the Ig heavy (*IGH*) chain (F) and light (*IGK/L*) chain (G) of immature and transitional B cells in ABOs. *Z* scores calculated from V-J gene pair frequencies are color coded.

(H) Visualization of the rearranged *IGH* and *IGK/L* chains mapped onto the UMAP of the B cell developmental stages as identified in Figure 3. (A–H) Data represent $n = 4$ individual donors in independent cultures. See also Figure S4.

co-stimulation (Figure 5I), indicating induction of a key component required for antibody diversification. Moreover, *de novo* CSR toward IgG and IgA was evident in both memory CD27^{+}CD38^{-} and CD27^{hi}CD38^{+} ACSs (Figure 5J), with no switched B cells detected in day 35 ABO-CD19^{+} cells (Figures 5B and 5C). Correspondingly, culture supernatants contained detectable levels of secreted IgA, IgG, and IgM on day 11 post-stimulation (Figure 5K).

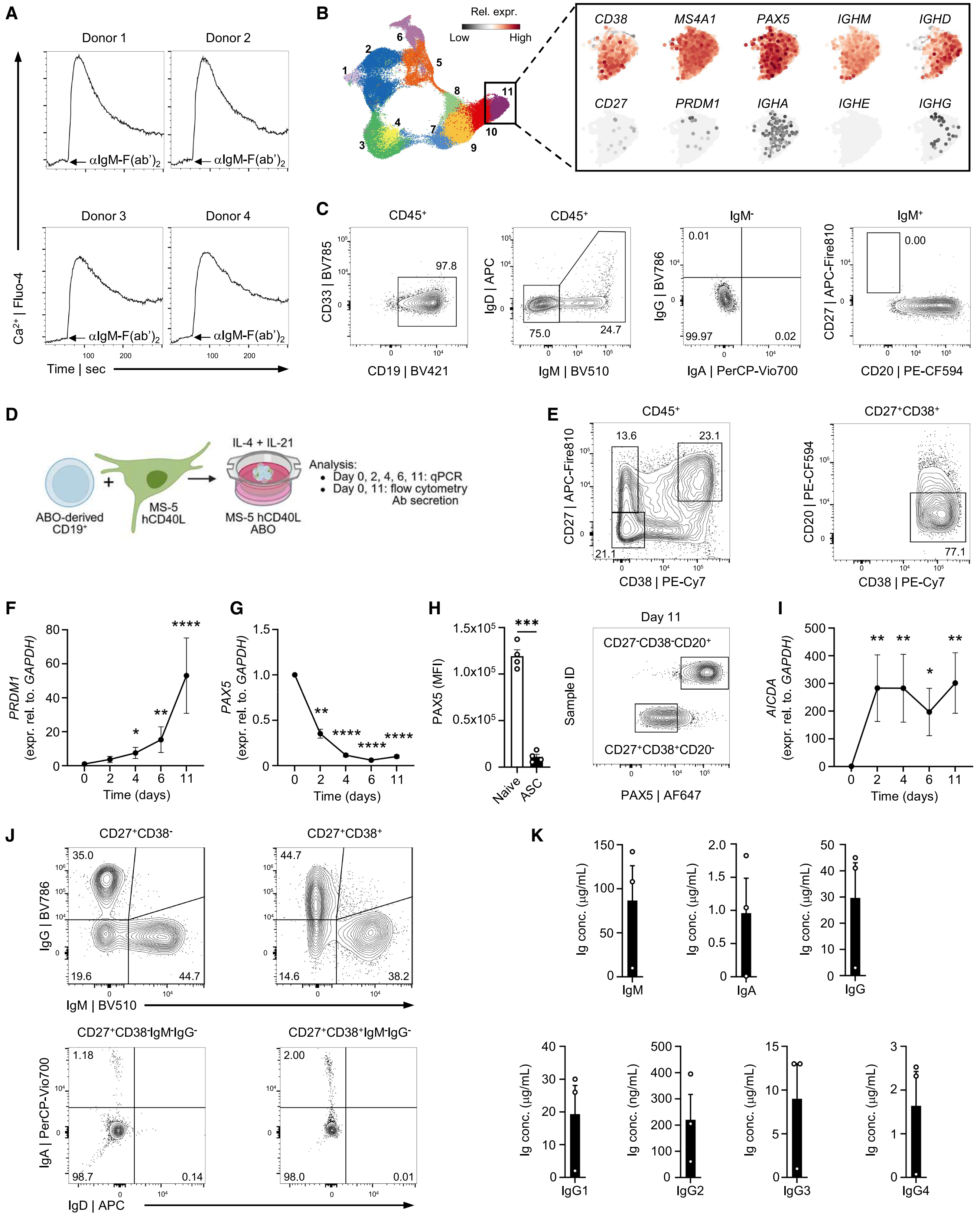

(legend on next page)

CellPress
OPEN ACCESS

These results demonstrate that ABOs generate immature and transitional B cells with functional BCRs that can differentiate into memory B cells and ASCs upon CD40 stimulation. Furthermore, these cells undergo *de novo* CSR and are likely to exhibit the capacity of SHM.

B cell development in ABOs is not driven by expansion of pre-existing B-lineage-committed progenitor cells

Since HSPCs contained CD10$^+$ B cell-committed progenitor cells (Figures 6A and S6A) that could accelerate or bias B cell development,[45] we evaluated whether depleting Lin$^+$CD10$^+$ cells from HSPCs (Figures 6A, S6B, and S6C) affected the efficiency of B cell development in ABOs. Depletion of CD10$^+$ progenitor cells had minimal effect on differentiation toward CD33$^+$ myeloid cells (Figures 6B and 6D) and did not alter the kinetics of lymphoid commitment as reflected by a similar appearance of CD10$^+$CD38$^+$ cells (Figures 6C and 6D) in day 21 ABOs like observed when using bulk CD34$^+$ HSPCs. Furthermore, both CD10-depleted and bulk CD34$^+$ HSPCs exhibited comparable capacity to generate IgM$^+$IgD$^-$ immature and IgM$^+$IgD$^+$ transitional B cells in ABOs (Figures 6E and 6F) and progressed through the expected stages of B cell development similarly (Figure 6G). Collectively, these results indicate that ABOs support B-lineage commitment directly from early HSPCs and do not depend on the expansion and development of pre-existing B-cell-committed progenitor cells.

RAG1 gene correction restores functional B-lymphopoiesis in RAG1-deficient mPB-derived HSPCs

To evaluate the utility of ABOs as a preclinical platform for gene therapy, we tested whether LV correction of HSPCs derived from patients with RAG1-SCID isolated from mobilized peripheral blood (mPB) could restore B cell development (Figure 7A). RAG1 is essential for V(D)J recombination,[46] and its deficiency blocks B cell development at the transition from the late pro-B to the pre-B stage (Figure 1B). Building on previous results showing that a coRAG1 LV vector restored T cell development in ATOs,[20] we employed ABOs to determine whether this strategy could similarly rescue B cell development.

We first assessed whether mPB-derived HSPCs were equally capable of developing into B cells compared to UCB-derived HSPCs. B cell commitment of mPB-derived HSPCs was lower than that of UCB-derived HSPCs, as demonstrated by reduced frequencies of CD10$^+$CD38$^+$ cells (Figure 7B). However, UCB- and mPB-derived HSPCs that had committed to the B-lineage equally progress through all B cell developmental stages (Figure 7C), albeit with slower kinetics when mPB-derived cells were used as the source, consistent with previous findings in ATOs.[17,20]

Next, RAG1-deficient mPB HSPCs were transduced with the pCCL.MND.coRAG1 LV vector used in our clinical trial (NCT04797260) and subsequently differentiated into B cells in ABOs. Detection of coRAG1 expression and viral copy number confirmed successful transduction (Figures S7A and S7B). Uncorrected RAG1-deficient mPB HSPCs failed to progress to the pre-B stage, as evidenced by markedly reduced frequencies of CD19$^+$ cells and absence of IgM-expressing cells (Figures 7D and 7E). Thus, ABOs recapitulated the expected developmental block caused by RAG1 deficiency. LV correction rescued B cell development, as demonstrated by the appearance of CD19$^+$ cells that further progressed along the B-cell lineage toward immature B cells, indicated by restored IgM expression, with a subset of cells that also co-expressed IgD (Figures 7D and 7E). In addition, coRAG1 LV transduction of RAG1-deficient mPB HSPCs restored Ig heavy- and light-chain recombination activity and diversity to levels comparable of healthy control mPB HSPCs (Figures 7F and S7C), indicating progression past the pre-BCR checkpoint and Ig rearrangement activity.

Finally, we evaluated whether B cells generated from coRAG1-transduced RAG1-deficient mPB HSPCs could undergo CSR and mature into ASCs. Reaggregation of ABO-B cells generated from corrected HSPCs derived from three patients with RAG1-SCID with MS-5-CD40L cells resulted in their differentiation into IgA− and IgG class-switched CD27$^+$CD38$^+$ cells (Figures 7G and 7H), with consequent Ig production in the culture medium (Figure 7I). The few B cells derived from uncorrected HSPCs of patient B—who also exhibited circulating B cells clinically (Table S1)—also differentiated into CD27$^+$CD38$^+$ cells, with a low frequency of IgM$^+$ and class-switched IgA$^+$ or IgG$^+$ cells in ABOs (Figure S7D). These findings demonstrate that ABOs can provide a suitable platform to evaluate the generation of functional B cells after correction of RAG1 deficiency in HSPCs derived from patients with RAG1-SCID using the coRAG1 LV vector.

Altogether, our findings establish ABOs as a versatile preclinical platform for modeling B-lymphopoiesis, enabling the

Figure 5. ABO-B cells express a functional BCR and can mature into class-switched ASCs

(A) Flow cytometric analysis of intracellular Ca^{2+} levels in day 35 ABO-CD19$^+$ cells following IgM crosslinking using αIgM-F(ab')$_2$.

(B) Gene expression levels of key relevant transcripts within cluster 11 cells (Figure 3).

(C) Flow cytometric analysis of day 35 ABO-CD19$^+$ cells.

(D) Schematic overview of stimulation cultures of day 35 ABO-CD19$^+$ cells. Created using BioRender (BioRender.com/6fbd5au).

(E) Flow cytometric analysis at day 11 post-stimulation of ABO-derived CD19$^+$ cells showing the presence of CD27$^+$CD38$^-$ memory B cells and CD20$^-$CD27$^+$CD38$^+$ ASCs.

(F and G) *PRDM1* (F) and *PAX5* (G) mRNA expression relative to *GAPDH* by qPCR analysis.

(H and J) Quantification (left) and representative flow cytometry plots (right) of PAX5 expression in CD27$^-$CD38$^-$CD20$^+$ naive B cells and CD27$^+$CD38$^+$CD20$^-$ ASCs. (MFI, mean fluorescent intensity) (I) *AICDA* mRNA expression relative to *GAPDH* by qPCR analysis.

(J) Flow cytometric analysis showing CSR in memory B cells and ASCs at day 11 of coculture.

(K) Quantification of antibody secretion (24 h) in culture supernatants collected from day 10–11 cultures.

(F–I) Statistical analysis was performed using paired Student's *t* test (H) or one-way ANOVA with Dunnett's multiple-comparison test (F, G, I) (*$p < 0.05$, **$p < 0.01$, ***$p < 0.001$, ****$p < 0.0001$). (F–I, K) Data are presented as mean ± SEM. Data represent $n = 3$ (C, E, J, K) and $n = 4$ (A, B, F–I) individual donors in independent cultures. See also Figure S5.

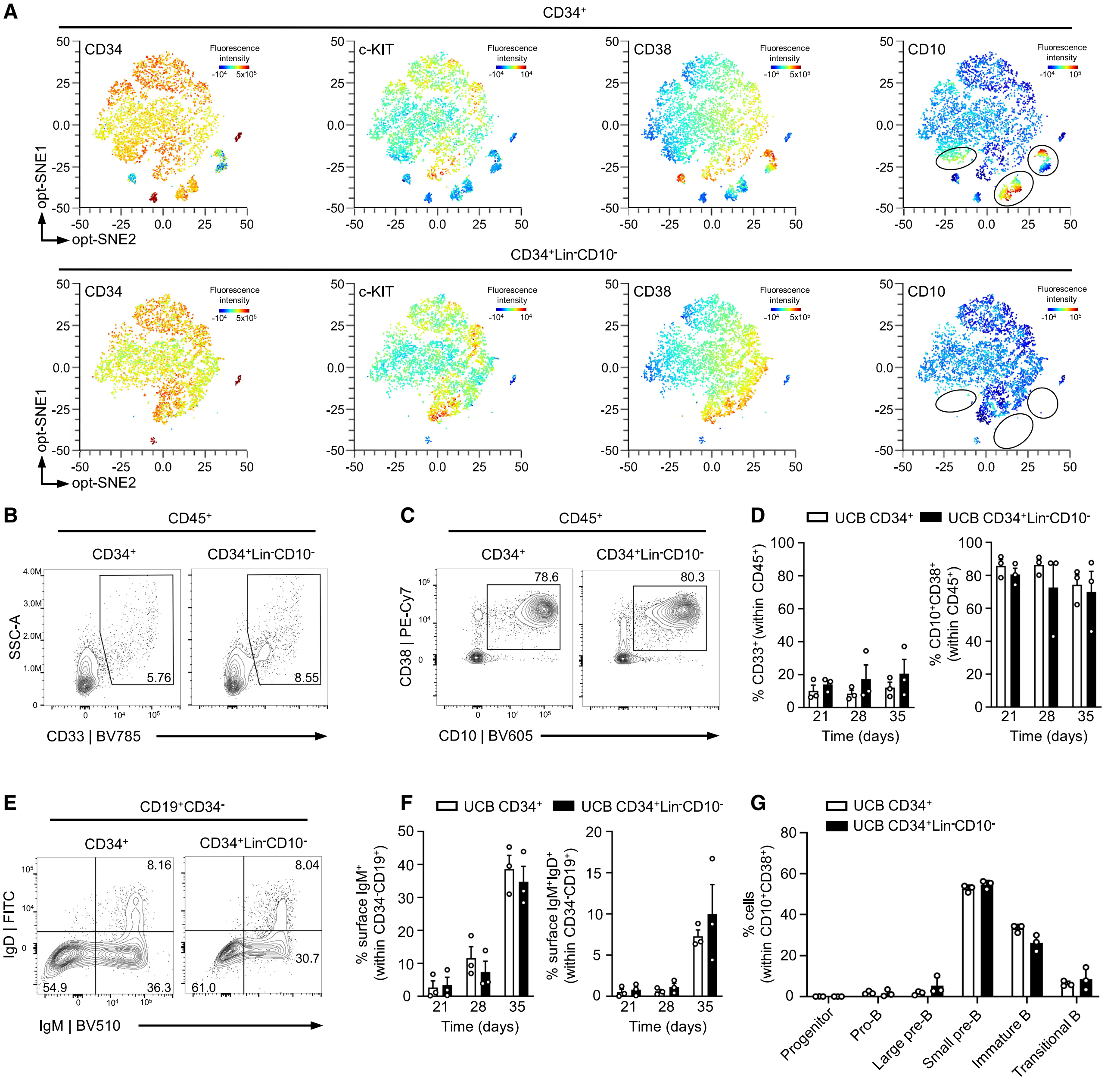

Figure 6. B cell development in ABOs is not driven by expansion of pre-existing B-lineage-committed progenitor cells

(A) Flow cytometric analysis of CD34, c-KIT (CD117), CD38, and CD10 expression on HSPCs before (top) and after depletion of Lin$^+$CD10$^+$ cells (bottom) by MACS, visualized with opt-SNE.

(B and C) Flow cytometric analysis comparing the frequencies of CD33$^+$ myeloid-committed (B) and CD10$^+$CD38$^+$ lymphoid-committed cells (C) in day 35 ABOs using bulk or Lin$^+$CD10$^+$-depleted CD34$^+$ HSPCs.

(D) Quantification of CD33$^+$ and CD10$^+$CD38$^+$ cells in day 35 ABOs.

(E) Flow cytometric analysis comparing IgM$^+$IgD$^-$ (immature) and IgM$^+$IgD$^+$ (transitional) B cell frequencies in day 35 ABOs using bulk or Lin$^+$CD10$^+$-depleted CD34$^+$ HSPCs.

(F) Quantification of IgM$^+$ and IgM$^+$IgD$^+$ B cell frequencies in day 35 ABOs.

(G) Distribution of HSPCs along the B cell developmental trajectory in day 35 ABOs. (D, F, G) Data are presented as mean ± SEM. Statistical analysis was performed using two-way ANOVA followed by Bonferroni's multiple comparisons test (ns; non-significant). (A–G) Data represent $n = 3$ individual donors in independent cultures. See also Figure S6.

CellPress
OPEN ACCESS

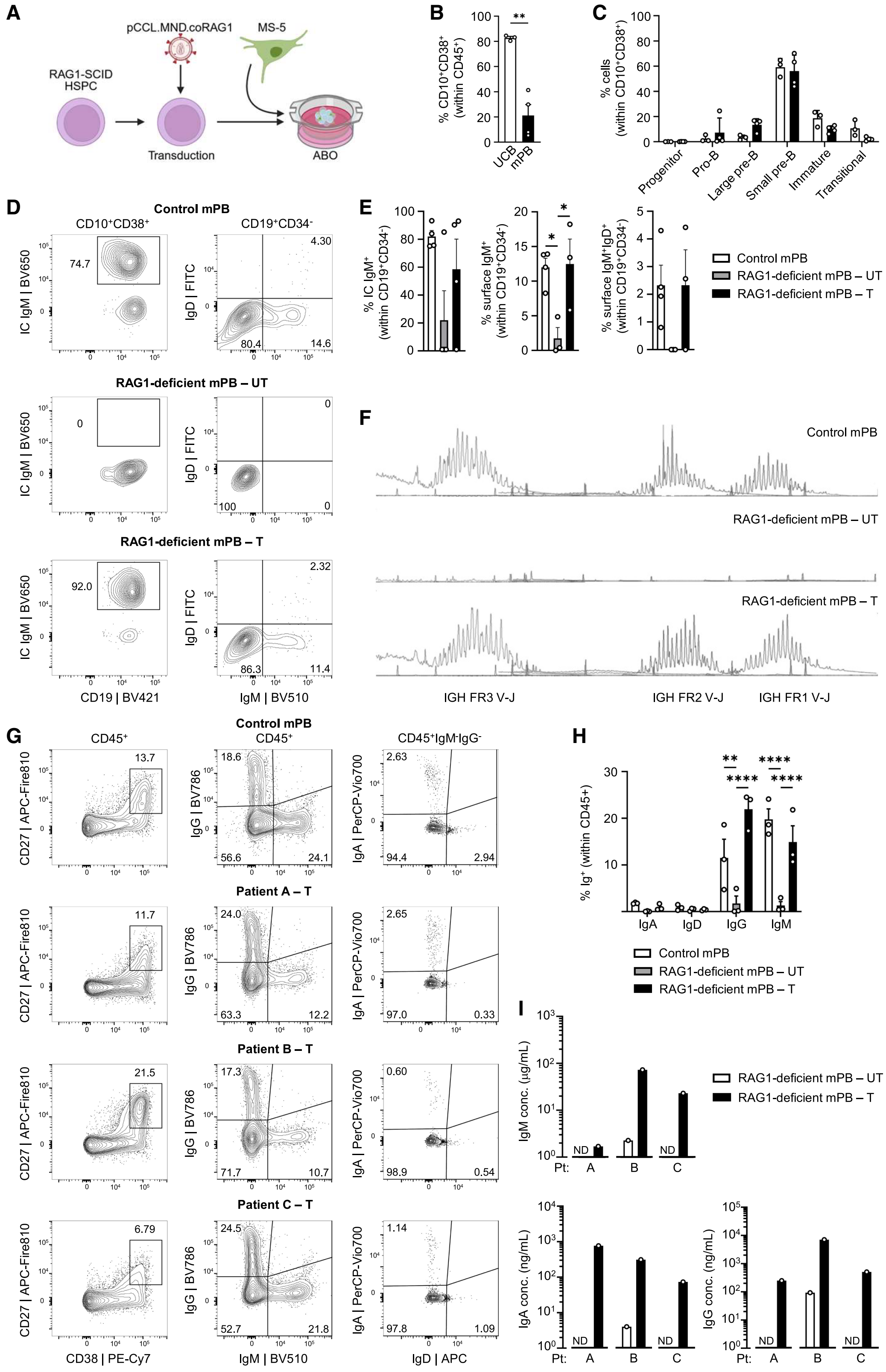

(legend on next page)

investigation of IEI-associated genetic mutations affecting B cell development—for instance for diagnostic purposes—and developing and testing of novel gene therapy strategies for B cell immunodeficiencies.

DISCUSSION

We present an *in vitro* spheroid-like culture system that supports complete B-lymphopoiesis from human UCB- and mPB-derived HSPCs. B cell development from UCB-derived HSPCs was more efficient than in established 2D monolayer or stromal free culture systems. Combined with the lower $CD34^+$ cell input required per ABO and shorter culture time, this system provides a valuable tool for studying B cell development, e.g., through genetic interference or drug screening approaches. Various culture models using UCB-, BM-, or iPSC-derived $CD34^+$ cells as HSPC source have been established. No studies, however, have investigated the use of mPB-derived HSPCs in *in vitro* B-lymphopoiesis cultures.[47] The ability to use adult-derived HSPCs makes this platform particularly attractive for testing preclinical gene therapy applications, since mPB-derived HSPCs are the clinically relevant stem cell source. The reduced output observed with mPB-derived cells is consistent with the lower proliferative and lymphoid differentiation capacity of adult- versus UCB-derived HSPCs as also reported in other *in vitro* culture systems such as ATOs[17,20] and *in vivo* xenograft transplantation models.[48] Hence, further optimization of culture conditions for adult-derived HSPCs may improve B cell output from ABOs and broaden platform applicability.

Three-dimensional spheroid-like culture systems that recapitulate T cell,[17] myeloid cell, and natural killer[49] cell development have emerged as valuable models for studying human hematopoiesis. Despite this progress, the reliable generation of functional B cells in such systems has not yet been achieved. Consistent with published studies,[12] our data indicate that current stroma-free cultures for B cell development are biased toward myeloid differentiation and generate few immature B cells, restricting their utility for applications that require robust B cell readouts. The inclusion of BM stromal cells promoted early lymphoid commitment, yet only ABOs supported the emergence of IgD^+ and/or IgM^+ B cells. These findings suggest that a 3D microenvironment maintained at an air-liquid interface more faithfully reproduces the spatial organization and cellular interactions that underpin B-lymphopoiesis, paralleling observations for T cell development in ATOs.[17] Moreover, culturing iPSCs in a 3D hydrogel-based system enhances their differentiation efficiency into HSPCs with better engraftment capacity and development into lymphoid and myeloid cells,[50–52] underscoring the importance of mimicking the 3D BM niche structure for modeling hematopoiesis *in vitro*.

The ABO system described here, however, represents a reductionist model for B cell development composed solely of a murine BM stromal cell line and HSPCs. The native BM niche integrates multiple mesenchymal and vascular components that secrete cytokines, chemokines, and extracellular matrix factors critical for lineage specification and maturation.[53,54] BM organoids[55–60] and BM-on-a-chip[61] models generated from induced pluripotent stem cells (iPSCs) provide important advances in recapitulating aspects of the human BM niche. Nevertheless, current BM organoid systems predominantly favor myeloid and erythroid differentiation over lymphoid output. Specifically, these iPSC-derived BM organoid/organ-on-a-chip models generate very few lymphoid cells, including $CD19^+$ B cells. In addition, B cells generated in these models are generally not further characterized for surface IgM/IgD expression, as presented in our study. A likely explanation is that the cytokine formulations used in these models are optimized to induce a vascular endothelial network (via vascular endothelial growth factors) and primarily support erythroid and myeloid differentiation of emerging $CD34^+$ cells, partly due to exogenous IL-3,[62,63] as well as absence of cytokines that support lymphoid cell development. This bias mirrors physiological BM hematopoiesis, where erythroid and myeloid progenitors quantitatively predominate over lymphoid progenitors.[64] $CD34^+$ cells emerging from these models, however, exhibit lymphoid potential, as demonstrated by their ability to generate B and T cells after *in vivo* engraftment[58] or their commitment to T cell development upon aggregation in ATOs.[58,60] We propose that ABOs, complementary to ATOs, provide a valuable tool to test B-lineage commitment potential of $CD34^+$ cells emerging from these organoid models. Moreover, UCB-derived HSPCs seeded in BM organoids differentiate into $CD19^+$ cells upon subcutaneous transplantation of whole BM organoids in athymic *Foxn1*$^{nu/nu}$ mice,[59] further suggesting that systemic factors required for efficient

Figure 7. RAG1 gene correction restores functional B-lymphopoiesis in RAG1-deficient mPB-derived HSPCs
(A) Schematic overview of coRAG1 LV correction and subsequent ABO culture of HSPCs derived from patients with RAG1-SCID. Created using BioRender (BioRender.com/qo00iis).
(B and C) Flow cytometric analysis of B cell commitment as assessed by CD10 and CD38 expression (B) and B-lineage progression (C) of healthy control UCB- and mPB-derived HSPCs in day 35 (UCB) and day 42 (mPB) ABOs, respectively.
(D) Flow cytometric analysis of day 42 ABOs using healthy control and RAG1-deficient mPB HSPCs (UT, untransduced; T, transduced).
(E) Quantification of the frequencies of intracellular (IC) IgM^+, IgM^+, and IgM^+IgD^+ cells in day 42 ABOs (UT, untransduced; T, transduced).
(F) Visualization of V_H-J_H rearrangements across conserved framework regions (FR1-3) of the *IGHV* locus by GeneScan analysis from healthy control (top), untransduced (UT; middle), and coRAG1-transduced (T; bottom) RAG1-deficient mPB HSPCs differentiated toward B cells in day 42 ABOs.
(G) Flow cytometric analysis of CD40-stimulated ABO-$CD19^+$ cells from control and transduced (T) RAG1-deficient mPB HSPCs on day 11.
(H) Distribution of IgM^+ and Ig class-switched cells at day 11 post-stimulation of ABO-$CD19^+$ cells using RAG1-deficient mPB HSPCs.
(I) Quantification of antibody secretion over 24 h in culture supernatants collected from day 10–11 cultures (ND, not detected). (B, C, E, H) Statistical analysis was performed using unpaired Student's *t* test (B), one-way ANOVA with Dunnett's multiple-comparison test (E), or two-way ANOVA with Bonferroni's multiple comparison test (C, H) (*$p < 0.05$, **$p < 0.01$, ****$p < 0.0001$). Data are presented as mean ± SEM. (B, C) Data represent $n = 3$ (UCB) and $n = 4$ (mPB) HSPC-derived ABOs. (D–I) Data represent $n = 4$ healthy control mPB and $n = 3$ RAG1-SCID-patient-derived mPB samples in independent cultures. See also Figure S7 and Table S1.

B-lymphopoiesis are absent in these organoid cultures. The ABO was specifically optimized to support B lineage development and maturation and therefore provides an attractive tool to either study B-lymphopoiesis, model B cell immunodeficiencies, and evaluate therapeutic strategies. Future studies in iPSC-derived BM organoids—containing a self-organized vascular network[58]—may integrate ABO-defined culture parameters to enable deeper understanding of human B cell development in health and disease and HSPC biology within BM niches.

Single-cell transcriptomic profiling confirmed that ABOs mimic human B-lymphopoiesis, with canonical marker gene expression patterns matching the developmental stages in healthy human BM. These include lineage-defining transcription factors and reciprocal *RAG1*/*RAG2* expression driving BCR rearrangements and genes associated with proliferation. Like human BM,[34,65] B cells developing in ABOs also showed higher RAG expression during Ig heavy-chain rearrangements versus light-chain rearrangements. Stage-specific cytokine and chemokine receptor expression as identified in ABO-B cells further underscores the physiological fidelity of the system. B cells generated in ABOs exhibited a highly diverse, polyclonal BCR repertoire with minimal clonal overlap between donors, reflecting physiological V(D)J recombination. Because ABOs mirror human B cell development at the transcriptional level, our scRNA-seq dataset may provide a resource to uncover potential novel regulators of B cell development from HSPCs, including growth factor receptors, transcriptional programs, or metabolic pathways. These can be interrogated directly in ABOs, for instance by assessing the impact of targeted growth factor supplementation on B cell development. Importantly, B cell development remained equally effective after depleting Lin^+ and $CD10^+$ cells from HSPCs. Including earlier ABO culture time points for transcriptome analysis could therefore illuminate the gene expression dynamics and regulatory mechanisms underlying commitment to lymphoid lineages and human B-lineage specification. Together with established ATOs,[17] the ABO platform provides a complementary and reproducible system for studying human lymphopoiesis *in vitro*, enabling mechanistic studies of T and B cell development.

To date, generating mature, functional B cells *in vitro* remains a major challenge, with B cell function typically assessed through xenograft transplantation of HSPCs into NSG mice.[21,66,67] We demonstrated that ABOs efficiently generate immature B cells that can undergo activation via BCR crosslinking or CD40 costimulation, exhibit *de novo* CSR, and have the capacity to mature into ASCs, including plasma cells. These results highlight the potential of ABOs as a versatile platform for engineering HSPCs for antibody production, enabling both *in vitro* antibody generation and transplantation of engineered HSPCs for long-term *in vivo* production. While HSPC programming for B cell-based vaccines is feasible,[10] scaling the ABO platform may pose technical challenges. Conversely, autologous transplantation of engineered HSPCs represents a realistic therapeutic avenue, supported by recent gene therapy applications.[68] Supporting translational relevance, we demonstrate in ABOs that HSPCs derived from patients with RAG1-SCID progressed through normal B cell development following LV RAG1 gene transfer, generating functional B cells capable of antibody production. For B cell-related IEIs, LV[69,70] or gene-editing-mediated restoration of BTK expression in HSPCs[16,71] rescues the developmental block in XLA, reconstituting B cell and antibody production in xenograft models. While engraftment potential cannot be assessed in our model, we propose that ABOs represent a promising preclinical platform for studying B cell development that may replace certain *in vivo* xenotransplantation models required for developing gene therapies for B cell-related IEIs[72] or precede them as an efficient screening tool.

ABOs can also serve as a valuable diagnostic and investigative tool for primary immune deficiencies, including CVIDs and SCIDs. For this purpose, we have used ATOs to accurately mimic the T cell arrest in cells from patients with RAG1-SCID included in our clinical trial (NCT04797260) and demonstrated that T cell development can be rescued after these patients' HSPCs were transduced with a self-inactivating LV vector encoding a coRAG1 transgene.[20,73] Similarly, gene editing in HSPCs to correct RAG1,[74] RAG2,[75] or CD3δ[76] deficiency generated HSPCs equally capable of differentiating into mature, functionally competent T cells, exhibiting a diverse T cell receptor (TCR) repertoire and robust responses to TCR stimulation compared to healthy-donor-derived HSPCs. We used ABOs to reproduce the block in B cell development observed in HSPCs from three patients with RAG1-SCID who had no naive T cells and reduced or no B cells. Two patients without B cells clinically also showed no B cell differentiation in ABOs. A third patient with RAG1-SCID, who presented clinically with markedly reduced B cell numbers, also led to the development of some IgM^+ and class-switched B cells, yet similar frequencies of intracellular IgM^+ cells comparable to those of coRAG1-corrected or healthy mPB HSPCs. This suggests that ABOs recapitulate not only the complete absence of but also incomplete B cell differentiation, mimicking deficiencies with and without residual B cells. Residual RAG activity can lead to successful V(D)J rearrangements of Ig heavy and light chains, but fails to generate a functional BCR, as shown in mice carrying hypomorphic RAG mutations.[77] Although rearranged Ig heavy chains were evident, the residual RAG1 activity was likely insufficient to drive successful Ig light-chain rearrangement, supporting the conclusion that these cells cannot assemble a functional BCR. Moreover, the few immature B cells that do develop from this patient's HSPCs may be enriched for self-reactive BCRs,[21,78] although this has not been confirmed by BCR repertoire analysis. Alternatively, CD40 stimulation of these B cells showed that, despite these cells expressing ASC markers, no surface IgM or IgA/IgG class switching occurred, suggesting that B cells generated from uncorrected HSPC of this patient are either nonfunctional or may have switched to IgE. While the latter is also observed in patients with hypomorphic RAG deficiency causing Omenn syndrome,[79] further studies using the ABO platform are warranted to better understand the B cell defect in such patients.

In summary, our findings establish ABOs as a reproducible and versatile platform for modeling human B cell development and dissecting disease-associated defects in B cell development and function. Complementing the ATO model, we propose that ABOs also provide a translational framework for advancing gene- and cell-based therapeutic strategies for IEIs affecting B cell development and/or function.

Limitations of the study

The stromal support in our system is provided by murine MS-5 cells, which were originally selected for their capacity to support B-lymphopoiesis.[24,26] Compared with OP9 stromal cells, MS-5 cells facilitate stronger HSPC adhesion[80] and appear better suited for 3D aggregate cocultures, consistent with previous observations for T cell development in ATOs.[17] However, ABOs rely on murine stromal support, which may influence the developmental trajectory and functional maturation of human HSPCs during B cell development. Incorporating human stromal support could improve the physiological relevance of the ABO platform and reduce cross-species interactions that may affect B cell development. However, human BM stromal cell lines may also adversely impact B-lymphopoiesis, as observed for T cell development in ATOs,[81] and should therefore be carefully evaluated. Another source of variability is fetal calf serum, which may contribute to batch-to-batch differences in B cell output. Future studies focused on using human stromal support and serum-free culture conditions or developing fully humanized systems such as iPSC-derived BM organoids may improve standardization of the ABO platform to help better define the molecular cues governing human B cell development using *in vitro* culture assays.

Negative selection of autoreactive B cells and B cell tolerance induction are critical for preventing autoreactivity. Autoreactive B cells that strongly recognize self-antigens are typically eliminated in the BM if receptor editing fails.[82] High-affinity clones are further selected during germinal center responses by SHM and affinity maturation.[83] While ABOs efficiently generate human immature and transitional B cells, these cells are likely not subjected to selection or tolerance checkpoints. Although CD40 stimulation induced *AICDA* expression in ABO-B cells, it also remains to be confirmed whether these cells undergo proper selection, SHM, and affinity maturation *in vivo*. However, absence of selection mechanisms in ABOs may also permit the generation of antibodies to self-antigens, including intracellular targets such as RAG proteins for which no antibodies are currently available.

RESOURCE AVAILABILITY

Lead contact

Requests for further information and resources should be directed to and will be fulfilled by the lead contact, Sander de Kivit (s.de_kivit@lumc.nl).

Materials availability

All unique/stable reagents generated in this study are available from the lead contact or Frank Staal (f.j.t.staal@lumc.nl), with a completed materials transfer agreement for academic institutions. For commercial purposes, please contact LUMC's Technology Transfer Office, Luris.

Data and code availability

- Single-cell RNA sequencing data have been deposited at the European Genome-phenome Archive as EGA: EGAD50000002433 and are publicly available as of the date of publication. This paper analyzes existing, publicly available data, accessible at GEO: GSE214693 and GEO: GSE289435.
- This paper does not report original code.
- Any additional information required to reanalyze the data reported in this paper is available from the lead contact upon request.

ACKNOWLEDGMENTS

We thank Dr. Yanling Xiao, Dr. Fiamma Salerno, and Dr. Erik van den Akker for insightful discussions, Dr. Szymon Kielbasa for assistance in scRNA-seq data analysis, Edwin Quinten, BSc, for providing reagents and technical assistance in Ca^{2+}-flux measurements, and the flow cytometry core facilities of the LUMC and the Leiden Genome Technology Center for technical assistance. We also thank the MID laboratory technicians of the Department of Immunology at the Erasmus MC in Rotterdam for performing the GeneScan analysis. This work was financially supported by a Proof of Concept grant awarded to F.J.T.S., K.C.-B., and S.d.K. from the reNEW consortium, the Novo Nordisk Foundation for Stem Cell Research (NNF21CC0073729) and an NWA-ORC grant from NWO (NWA.1389.20.049) awarded to F.J.T.S. The development of the clinical grade RAG1 lentiviral vector was supported by EU H2020 grant RECOMB (755170-2) awarded to F.J.T.S., as funding from the European Union Horizon 2020 research and innovation program.

AUTHOR CONTRIBUTIONS

Conceptualization, M.B., K.P.-O., F.J.T.S., K.C.-B., and S.d.K.; methodology, M.B., K.P.-O., F.J.T.S., K.C.-B., and S.d.K.; investigation, M.B., M.C., B.d.M., S.A.V., S.d.B.-V., A.W., A.W.L., K.C.-B., and S.d.K.; formal analysis, M.B., M.C., B.d.M., S.A.V., S.d.B.-V., A.W.L., K.C.-B., and S.d.K.; writing – original draft, M.B., M.C., K.C.-B., and S.d.K.; writing – review and editing, M.B., K.P.-O., F.J.T.S., K.C.-B., and S.d.K.; visualization, M.B., M.C., and S.d.K.; supervision, K.P.-O., F.J.T.S., K.C.-B., and S.d.K.; funding acquisition, F.J.T.S., K.C.-B., and S.d.K.

DECLARATION OF INTERESTS

The LUMC has filed for a patent protecting the ABO technology for commercial purposes. Companies are advised to contact the LUMC's Technology Transfer Office, Luris, in accordance with LUMC regulations. None of the authors have a commercial interest in the described technology.

STAR★METHODS

Detailed methods are provided in the online version of this paper and include the following:

- KEY RESOURCES TABLE
- EXPERIMENTAL MODEL AND STUDY PARTICIPANTS DETAILS
  - Human study and ethics statement
  - Animal studies and ethics
  - Cell lines
- METHOD DETAILS
  - Hematopoietic stem and progenitor cell isolation
  - B cell development cultures
  - B-cell activation cultures
  - Flow cytometry
  - Ca^{2+} flux measurements
  - Antibody production measurements
  - Sample and library preparation for scRNA-seq and scBCR-seq
  - Processing of scRNA-seq data
  - scRNA-seq data analysis
  - scBCR repertoire analysis
  - LV transduction of HSPCs derived from patients with RAG1-SCID
  - Quantitative PCR
  - PrimeFlow analysis
  - GeneScan analysis
- QUANTIFICATION AND STATISTICAL ANALYSIS

SUPPLEMENTAL INFORMATION

Supplemental information can be found online at https://doi.org/10.1016/j.xcrm.2026.102879.

Received: November 3, 2025
Revised: April 2, 2026
Accepted: May 26, 2026
Published: June 18, 2026

REFERENCES

1. Morrison, S.J., and Scadden, D.T. (2014). The bone marrow niche for haematopoietic stem cells. Nature *505*, 327–334. https://doi.org/10.1038/nature12984.
2. Nandiwada, S.L. (2023). Overview of human B-cell development and antibody deficiencies. J. Immunol. Methods *519*, 113485. https://doi.org/10.1016/j.jim.2023.113485.
3. Lin, Y.C., Jhunjhunwala, S., Benner, C., Heinz, S., Welinder, E., Mansson, R., Sigvardsson, M., Hagman, J., Espinoza, C.A., Dutkowski, J., et al. (2010). A global network of transcription factors, involving E2A, EBF1 and Foxo1, that orchestrates B cell fate. Nat. Immunol. *11*, 635–643. https://doi.org/10.1038/ni.1891.
4. Kaiser, F.M.P., Janowska, I., Menafra, R., de Gier, M., Korzhenevich, J., Pico-Knijnenburg, I., Khatri, I., Schulz, A., Kuijpers, T.W., Lankester, A.C., et al. (2023). IL-7 receptor signaling drives human B-cell progenitor differentiation and expansion. Blood *142*, 1113–1130. https://doi.org/10.1182/blood.2023019721.
5. Tangye, S.G., Nguyen, T., Deenick, E.K., Bryant, V.L., and Ma, C.S. (2023). Inborn errors of human B cell development, differentiation, and function. J. Exp. Med. *220*, e20221105. https://doi.org/10.1084/jem.20221105.
6. Vetrie, D., Vořechovský, I., Sideras, P., Holland, J., Davies, A., Flinter, F., Hammarström, L., Kinnon, C., Levinsky, R., Bobrow, M., et al. (1993). The gene involved in X-linked agammaglobulinaemia is a member of the src family of protein-tyrosine kinases. Nature *361*, 226–233. https://doi.org/10.1038/361226a0.
7. Hendriks, R.W., Bredius, R.G., Pike-Overzet, K., and Staal, F.J. (2011). Biology and novel treatment options for XLA, the most common monogenetic immunodeficiency in man. Expert Opin. Ther. Targets *15*, 1003–1021. https://doi.org/10.1517/14728222.2011.585971.
8. Noordzij, J.G., de Bruin-Versteeg, S., Verkaik, N.S., Vossen, J.M., de Groot, R., Bernatowska, E., Langerak, A.W., van Gent, D.C., and van Dongen, J.J. (2002). The immunophenotypic and immunogenotypic B-cell differentiation arrest in bone marrow of RAG-deficient SCID patients corresponds to residual recombination activities of mutated RAG proteins. Blood *100*, 2145–2152.
9. Kohn, L.A., and Kohn, D.B. (2021). Gene Therapies for Primary Immune Deficiencies. Front. Immunol. *12*, 648951. https://doi.org/10.3389/fimmu.2021.648951.
10. Luo, X.M., Maarschalk, E., O'Connell, R.M., Wang, P., Yang, L., and Baltimore, D. (2009). Engineering human hematopoietic stem/progenitor cells to produce a broadly neutralizing anti-HIV antibody after in vitro maturation to human B lymphocytes. Blood *113*, 1422–1431. https://doi.org/10.1182/blood-2008-09-177139.
11. Ichii, M., Oritani, K., Yokota, T., Schultz, D.C., Holter, J.L., Kanakura, Y., and Kincade, P.W. (2010). Stromal cell-free conditions favorable for human B-lymphopoiesis in culture. J. Immunol. Methods *359*, 47–55. https://doi.org/10.1016/j.jim.2010.06.002.
12. Kraus, H., Kaiser, S., Aumann, K., Bönelt, P., Salzer, U., Vestweber, D., Erlacher, M., Kunze, M., Burger, M., Pieper, K., et al. (2014). A feeder-free differentiation system identifies autonomously proliferating B cell precursors in human bone marrow. J. Immunol. *192*, 1044–1054. https://doi.org/10.4049/jimmunol.1301815.
13. Yang, P., Chen, X., Wen, H., Yu, M., Yu, H., Wang, L., Gong, L., and Zhao, L. (2025). In vitro differentiation of common lymphoid progenitor cells into B cell using stromal cell free culture system. BMC Immunol. *26*, 12. https://doi.org/10.1186/s12865-025-00694-8.
14. Troilo, A., Wehr, C., Janowska, I., Venhoff, N., Thiel, J., Rawluk, J., Frede, N., Staniek, J., Lorenzetti, R., Schleyer, M.T., et al. (2020). Nonpermissive bone marrow environment impairs early B-cell development in common variable immunodeficiency. Blood *135*, 1452–1457. https://doi.org/10.1182/blood.2019003855.
15. Thiel, J., Schmidt, F.M., Lorenzetti, R., Troilo, A., Janowska, I., Nießen, L., Pfeiffer, S., Staniek, J., Benassini, B., Bott, M.T., et al. (2024). Defects in B-lymphopoiesis and B-cell maturation underlie prolonged B-cell depletion in ANCA-associated vasculitis. Ann. Rheum. Dis. *83*, 1536–1548. https://doi.org/10.1136/ard-2024-225587.
16. Bahal, S., Zinicola, M., Moula, S.E., Whittaker, T.E., Schejtman, A., Naseem, A., Blanco, E., Vetharoy, W., Hu, Y.T., Rai, R., et al. (2024). Hematopoietic stem cell gene editing rescues B-cell development in X-linked agammaglobulinemia. J. Allergy Clin. Immunol. *154*, 195–208.e8. https://doi.org/10.1016/j.jaci.2024.03.003.
17. Seet, C.S., He, C., Bethune, M.T., Li, S., Chick, B., Gschweng, E.H., Zhu, Y., Kim, K., Kohn, D.B., Baltimore, D., et al. (2017). Generation of mature T cells from human hematopoietic stem and progenitor cells in artificial thymic organoids. Nat. Methods *14*, 521–530. https://doi.org/10.1038/nmeth.4237.
18. Cordes, M., Canté-Barrett, K., van den Akker, E.B., Moretti, F.A., Kiełbasa, S.M., Vloemans, S.A., Garcia-Perez, L., Teodosio, C., van Dongen, J.J.M., Pike-Overzet, K., et al. (2022). Single-cell immune profiling reveals thymus-seeding populations, T cell commitment, and multilineage development in the human thymus. Sci. Immunol. *7*, eade0182. https://doi.org/10.1126/sciimmunol.ade0182.
19. Bosticardo, M., Pala, F., Calzoni, E., Delmonte, O.M., Dobbs, K., Gardner, C.L., Sacchetti, N., Kawai, T., Garabedian, E.K., Draper, D., et al. (2020). Artificial thymic organoids represent a reliable tool to study T-cell differentiation in patients with severe T-cell lymphopenia. Blood Adv. *4*, 2611–2616. https://doi.org/10.1182/bloodadvances.2020001730.
20. Meng, X., Melsen, J.E., van der Holst, R., de Mooij, B., Vloemans, S., van Eggermond, M., Berghuis, D., Lankester, A.C., de Kivit, S., Pike-Overzet, K., et al. (2025). RAG1 lentiviral gene therapy restores T cell development of RAG1-SCID patient cells in artificial thymic organoids. Blood Adv. *9*, 5920–5934. https://doi.org/10.1182/bloodadvances.2025016970.
21. Bosticardo, M., Dobbs, K., Delmonte, O.M., Martins, A.J., Pala, F., Kawai, T., Kenney, H., Magro, G., Rosen, L.B., Yamazaki, Y., et al. (2025). Multiomics dissection of human RAG deficiency reveals distinctive patterns of immune dysregulation but a common inflammatory signature. Sci. Immunol. *10*, eadq1697. https://doi.org/10.1126/sciimmunol.adq1697.
22. Bifsha, P., Leiding, J.W., Pai, S.Y., Colamartino, A.B.L., Hartog, N., Church, J.A., Oshrine, B.R., Puck, J.M., Markert, M.L., and Haddad, E. (2020). Diagnostic assay to assist clinical decisions for unclassified severe combined immune deficiency. Blood Adv. *4*, 2606–2610. https://doi.org/10.1182/bloodadvances.2020001736.
23. Schmitt, T.M., and Zúñiga-Pflücker, J.C. (2002). Induction of T cell development from hematopoietic progenitor cells by delta-like-1 in vitro. Immunity *17*, 749–756. https://doi.org/10.1016/s1074-7613(02)00474-0.
24. Itoh, K., Tezuka, H., Sakoda, H., Konno, M., Nagata, K., Uchiyama, T., Uchino, H., and Mori, K.J. (1989). Reproducible establishment of hemopoietic supportive stromal cell lines from murine bone marrow. Exp. Hematol. *17*, 145–153.
25. Ohkawara, J.I., Ikebuchi, K., Fujihara, M., Sato, N., Hirayama, F., Yamaguchi, M., Mori, K.J., and Sekiguchi, S. (1998). Culture system for extensive production of CD19+IgM+ cells by human cord blood CD34+ progenitors. Leukemia *12*, 764–771. https://doi.org/10.1038/sj.leu.2401004.
26. Nishihara, M., Wada, Y., Ogami, K., Ebihara, Y., Ishii, T., Tsuji, K., Ueno, H., Asano, S., Nakahata, T., and Maekawa, T. (1998). A combination of stem cell factor and granulocyte colony-stimulating factor enhances the growth of human progenitor B cells supported by murine stromal cell line MS-5. Eur. J. Immunol. *28*, 855–864. https://doi.org/10.1002/(SICI)1521-4141(199803)28:03<855::AID-IMMU855>3.0.CO;2-X.
27. Martin, C.H., Woll, P.S., Ni, Z., Zúñiga-Pflücker, J.C., and Kaufman, D.S. (2008). Differences in lymphocyte developmental potential between human embryonic stem cell and umbilical cord blood-derived hematopoietic

progenitor cells. Blood *112*, 2730–2737. https://doi.org/10.1182/blood-2008-01-133801.

28. Richardson, S.E., Ghazanfari, R., Chhetri, J., Enver, T., and Böiers, C. (2021). In vitro differentiation of human pluripotent stem cells into the B lineage using OP9-MS5 co-culture. STAR Protoc. *2*, 100420. https://doi.org/10.1016/j.xpro.2021.100420.
29. Nagasawa, T. (2006). Microenvironmental niches in the bone marrow required for B-cell development. Nat. Rev. Immunol. *6*, 107–116. https://doi.org/10.1038/nri1780.
30. Nakamori, Y., Liu, B., Ohishi, K., Suzuki, K., Ino, K., Matsumoto, T., Masuya, M., Nishikawa, H., Shiku, H., Hamada, H., and Katayama, N. (2012). Human bone marrow stromal cells simultaneously support B and T/NK lineage development from human haematopoietic progenitors: a principal role for flt3 ligand in lymphopoiesis. Br. J. Haematol. *157*, 674–686. https://doi.org/10.1111/j.1365-2141.2012.09109.x.
31. Encabo, A., Mateu, E., Carbonell-Uberos, F., and Minana, M.D. (2003). IL-6 precludes the differentiation induced by IL-3 on expansion of CD34+ cells from cord blood. Haematologica *88*, 388–395.
32. Kikuchi, K., Lai, A.Y., Hsu, C.L., and Kondo, M. (2005). IL-7 receptor signaling is necessary for stage transition in adult B cell development through up-regulation of EBF. J. Exp. Med. *201*, 1197–1203. https://doi.org/10.1084/jem.20050158.
33. Parrish, Y.K., Baez, I., Milford, T.A., Benitez, A., Galloway, N., Rogerio, J.W., Sahakian, E., Kagoda, M., Huang, G., Hao, Q.L., et al. (2009). IL-7 Dependence in human B-lymphopoiesis increases during progression of ontogeny from cord blood to bone marrow. J. Immunol. *182*, 4255–4266. https://doi.org/10.4049/jimmunol.0800489.
34. Zeng, A.G.X., Iacobucci, I., Shah, S., Mitchell, A., Wong, G., Bansal, S., Chen, D., Gao, Q., Kim, H., Kennedy, J.A., et al. (2025). Single-cell Transcriptional Atlas of Human Hematopoiesis Reveals Genetic and Hierarchy-Based Determinants of Aberrant AML Differentiation. Blood Cancer Discov. *6*, 307–324. https://doi.org/10.1158/2643-3230.BCD-24-0342.
35. Boyd, S.D., Gaëta, B.A., Jackson, K.J., Fire, A.Z., Marshall, E.L., Merker, J.D., Maniar, J.M., Zhang, L.N., Sahaf, B., Jones, C.D., et al. (2010). Individual variation in the germline Ig gene repertoire inferred from variable region gene rearrangements. J. Immunol. *184*, 6986–6992. https://doi.org/10.4049/jimmunol.1000445.
36. DeKosky, B.J., Lungu, O.I., Park, D., Johnson, E.L., Charab, W., Chrysostomou, C., Kuroda, D., Ellington, A.D., Ippolito, G.C., Gray, J.J., and Georgiou, G. (2016). Large-scale sequence and structural comparisons of human naive and antigen-experienced antibody repertoires. Proc. Natl. Acad. Sci. USA *113*, E2636–E2645. https://doi.org/10.1073/pnas.1525510113.
37. Briney, B., Inderbitzin, A., Joyce, C., and Burton, D.R. (2019). Commonality despite exceptional diversity in the baseline human antibody repertoire. Nature *566*, 393–397. https://doi.org/10.1038/s41586-019-0879-y.
38. Belessi, C., Stamatopoulos, K., Hadzidimitriou, A., Hatzi, K., Smilevska, T., Stavroyianni, N., Marantidou, F., Paterakis, G., Fassas, A., Anagnostopoulos, A., and Laoutaris, N. (2005). Analysis of expressed and non-expressed IGK locus rearrangements in chronic lymphocytic leukemia. Mol. Med. *11*, 52–58. https://doi.org/10.2119/2005-00044.Belessi.
39. Forconi, F., Sozzi, E., Rossi, D., Sahota, S.S., Amato, T., Raspadori, D., Trentin, L., Leoncini, L., Gaidano, G., and Lauria, F. (2008). Selective influences in the expressed immunoglobulin heavy and light chain gene repertoire in hairy cell leukemia. Haematologica *93*, 697–705. https://doi.org/10.3324/haematol.12282.
40. Young, C., and Brink, R. (2021). The unique biology of germinal center B cells. Immunity *54*, 1652–1664. https://doi.org/10.1016/j.immuni.2021.07.015.
41. Mora-López, F., Pedreño-Horrillo, N., Delgado-Pérez, L., Brieva, J.A., and Campos-Caro, A. (2008). Transcription of PRDM1, the master regulator for plasma cell differentiation, depends on an SP1/SP3/EGR-1 GC-box. Eur. J. Immunol. *38*, 2316–2324. https://doi.org/10.1002/eji.200737861.
42. Lin, K.I., Angelin-Duclos, C., Kuo, T.C., and Calame, K. (2002). Blimp-1-dependent repression of Pax-5 is required for differentiation of B cells to immunoglobulin M-secreting plasma cells. Mol. Cell Biol. *22*, 4771–4780. https://doi.org/10.1128/MCB.22.13.4771-4780.2002.
43. Nera, K.P., Kohonen, P., Narvi, E., Peippo, A., Mustonen, L., Terho, P., Koskela, K., Buerstedde, J.M., and Lassila, O. (2006). Loss of Pax5 promotes plasma cell differentiation. Immunity *24*, 283–293. https://doi.org/10.1016/j.immuni.2006.02.003.
44. Muramatsu, M., Kinoshita, K., Fagarasan, S., Yamada, S., Shinkai, Y., and Honjo, T. (2000). Class switch recombination and hypermutation require activation-induced cytidine deaminase (AID), a potential RNA editing enzyme. Cell *102*, 553–563. https://doi.org/10.1016/s0092-8674(00)00078-7.
45. Xiao, Y., Zijl, S., Wang, L., de Groot, D.C., van Tol, M.J., Lankester, A.C., and Borst, J. (2015). Identification of the Common Origins of Osteoclasts, Macrophages, and Dendritic Cells in Human Hematopoiesis. Stem Cell Rep. *4*, 984–994. https://doi.org/10.1016/j.stemcr.2015.04.012.
46. Braams, M., Pike-Overzet, K., and Staal, F.J.T. (2023). The recombinase activating genes: architects of immune diversity during lymphocyte development. Front. Immunol. *14*, 1210818. https://doi.org/10.3389/fimmu.2023.1210818.
47. Schmidt, F.M., and Rizzi, M. (2026). Human B-lymphopoiesis: Clinical challenges in B cell reconstitution and advances in in vitro modeling. Immunol. Lett. *277*, 107106. https://doi.org/10.1016/j.imlet.2025.107106.
48. Han, A.R., Lee, J.E., Lee, M.J., Ko, S.Y., Shin, H.S., Lee, J.Y., and Lee, D.R. (2021). Distinct Repopulation Activity in Hu-Mice Between CB- and LPB-CD34(+) Cells by Enrichment of Transcription Factors. Int. J. Stem Cells *14*, 203–211. https://doi.org/10.15283/ijsc21015.
49. Schell, B., Zhao, L.P., M'Sibih, I., Kalogeraki, M., Kergaravat, C., Lereclus, E., Fenaux, P., Adès, L., Toubert, A., Espéli, M., et al. (2025). Modeling mesenchymal stromal cell support to hematopoiesis within a novel 3D artificial marrow organoid system. Sci. Rep. *15*, 23603. https://doi.org/10.1038/s41598-025-07717-9.
50. Xu, Y., Shan, W., Li, X., Wang, B., Liu, S., Wang, Y., Long, Y., Tie, R., Wang, L., Cai, S., et al. (2016). A synthetic three-dimensional niche system facilitates generation of functional hematopoietic cells from human-induced pluripotent stem cells. J. Hematol. Oncol. *9*, 102. https://doi.org/10.1186/s13045-016-0326-6.
51. Sidhu, I., Barwe, S.P., Kiick, K.L., Kolb, E.A., and Gopalakrishnapillai, A. (2021). A 3-D hydrogel based system for hematopoietic differentiation and its use in modeling down syndrome associated transient myeloproliferative disorder. Biomater. Sci. *9*, 6266–6281. https://doi.org/10.1039/d1bm00442e.
52. Bruschi, M., Vanzolini, T., Sahu, N., Balduini, A., Magnani, M., and Fraternale, A. (2022). Functionalized 3D scaffolds for engineering the hematopoietic niche. Front. Bioeng. Biotechnol. *10*, 968086. https://doi.org/10.3389/fbioe.2022.968086.
53. Crane, G.M., Jeffery, E., and Morrison, S.J. (2017). Adult haematopoietic stem cell niches. Nat. Rev. Immunol. *17*, 573–590. https://doi.org/10.1038/nri.2017.53.
54. Tikhonova, A.N., Dolgalev, I., Hu, H., Sivaraj, K.K., Hoxha, E., Cuesta-Domínguez, Á., Pinho, S., Akhmetzyanova, I., Gao, J., Witkowski, M., et al. (2019). The bone marrow microenvironment at single-cell resolution. Nature *569*, 222–228. https://doi.org/10.1038/s41586-019-1104-8.
55. Khan, A.O., Rodriguez-Romera, A., Reyat, J.S., Olijnik, A.A., Colombo, M., Wang, G., Wen, W.X., Sousos, N., Murphy, L.C., Grygielska, B., et al. (2023). Human Bone Marrow Organoids for Disease Modeling, Discovery, and Validation of Therapeutic Targets in Hematologic Malignancies. Cancer Discov. *13*, 364–385. https://doi.org/10.1158/2159-8290.CD-22-0199.
56. Olijnik, A.A., Rodriguez-Romera, A., Wong, Z.C., Shen, Y., Reyat, J.S., Jooss, N.J., Rayes, J., Psaila, B., and Khan, A.O. (2024). Generating human bone marrow organoids for disease modeling and drug discovery. Nat. Protoc. *19*, 2117–2146. https://doi.org/10.1038/s41596-024-00971-7.

57. Ren, K., Li, E., Aydemir, I., Liu, Y., Han, X., Bi, H., Wang, P., Tao, K., Ji, A., Chen, Y.H., et al. (2025). Development of iPSC-derived human bone marrow organoid for autonomous hematopoiesis and patient-derived HSPC engraftment. Blood Adv. *9*, 54–65. https://doi.org/10.1182/bloodadvances.2024013361.

58. Frenz-Wiessner, S., Fairley, S.D., Buser, M., Goek, I., Salewskij, K., Jonsson, G., Illig, D., Zu Putlitz, B., Petersheim, D., Li, Y., et al. (2024). Generation of complex bone marrow organoids from human induced pluripotent stem cells. Nat. Methods *21*, 868–881. https://doi.org/10.1038/s41592-024-02172-2.

59. Li, Q., Nikolova, M.T., Zhang, G., Cervenka, I., Valigi, F., Burri, D., Plantier, E., Mazzoleni, A., Lamouline, A., Schwaller, J., et al. (2025). Macro-scale, scaffold-assisted model of the human bone marrow endosteal niche using hiPSC-vascularized osteoblastic organoids. Cell Stem Cell *32*, 1941–1958.e8. https://doi.org/10.1016/j.stem.2025.10.009.

60. Shen, Y., Benlabiod, C., Watson, E., Gurashi, K., Fower, A., Rodriguez-Romera, A., Reyat, J.S., Adnan-Awad, S., Hargreaves, R., Kemble, S., et al. (2026). comBO: A combined human bone and lympho-myeloid bone marrow organoid for preclinical modeling of hematopoietic disorders. Cell Stem Cell *33*, 421–437.e7. https://doi.org/10.1016/j.stem.2026.01.010.

61. Georgescu, A., Oved, J.H., Galarraga, J.H., Cantrell, T., Mehta, S., Dulmovits, B.M., Olson, T.S., Fattahi, P., Wang, A., Candarlioglu, P.L., et al. (2024). Self-organization of the hematopoietic vascular niche and emergent innate immunity on a chip. Cell Stem Cell *31*, 1847–1864.e6. https://doi.org/10.1016/j.stem.2024.11.003.

62. Tajer, P., Canté-Barrett, K., Naber, B.A.E., Vloemans, S.A., van Eggermond, M.C.J.A., van der Hoorn, M.L., Pike-Overzet, K., and Staal, F.J.T. (2022). IL3 Has a Detrimental Effect on Hematopoietic Stem Cell Self-Renewal in Transplantation Settings. Int. J. Mol. Sci. *23*, 12736. https://doi.org/10.3390/ijms232112736.

63. Suda, T., Suda, J., Ogawa, M., and Ihle, J.N. (1985). Permissive role of interleukin 3 (IL-3) in proliferation and differentiation of multipotential hemopoietic progenitors in culture. J. Cell. Physiol. *124*, 182–190. https://doi.org/10.1002/jcp.1041240203.

64. de Sousa, F.A., Correa, R.P., Bento, L.C., Taniguchi, L.F.P., Bacal, N.S., and Marti, L.C. (2025). Immunophenotypic Profile of Normal Hematopoietic Populations in Human Bone Marrow: Influence of Gender and Aging as a Basis for Reference Value Establishment. Cells *14*, 1392. https://doi.org/10.3390/cells14171392.

65. van Zelm, M.C., van der Burg, M., de Ridder, D., Barendregt, B.H., de Haas, E.F.E., Reinders, M.J.T., Lankester, A.C., Révész, T., Staal, F.J.T., and van Dongen, J.J.M. (2005). Ig gene rearrangement steps are initiated in early human precursor B cell subsets and correlate with specific transcription factor expression. J. Immunol. *175*, 5912–5922. https://doi.org/10.4049/jimmunol.175.9.5912.

66. Garcia-Perez, L., van Eggermond, M., van Roon, L., Vloemans, S.A., Cordes, M., Schambach, A., Rothe, M., Berghuis, D., Lagresle-Peyrou, C., Cavazzana, M., et al. (2020). Successful Preclinical Development of Gene Therapy for Recombinase-Activating Gene-1-Deficient SCID. Mol. Ther. Methods Clin. Dev. *17*, 666–682. https://doi.org/10.1016/j.omtm.2020.03.016.

67. Sorel, N., Díaz-Pascual, F., Bessot, B., Sadek, H., Mollet, C., Chouteau, M., Zahn, M., Gil-Farina, I., Tajer, P., van Eggermond, M., et al. (2024). Restoration of T and B Cell Differentiation after RAG1 Gene Transfer in Human RAG1 Defective Hematopoietic Stem Cells. Biomedicines *12*, 1495. https://doi.org/10.3390/biomedicines12071495.

68. Mamede, L., Sabà, R.C., Van Coillie, S., Prévot, J., Sánchez-Ramón, S., Poli, C., Barasa, A., Schuller, B.W., Hendel, A., Garcelon, N., et al. (2025). Navigating disruption in the PID landscape: embracing opportunities and anticipating threats in the next ten years. Front. Immunol. *16*, 1596971. https://doi.org/10.3389/fimmu.2025.1596971.

69. Ng, Y.Y., Baert, M.R.M., Pike-Overzet, K., Rodijk, M., Brugman, M.H., Schambach, A., Baum, C., Hendriks, R.W., van Dongen, J.J.M., and Staal, F.J.T. (2010). Correction of B-cell development in Btk-deficient mice using lentiviral vectors with codon-optimized human BTK. Leukemia *24*, 1617–1630. https://doi.org/10.1038/leu.2010.140.

70. Seymour, B.J., Singh, S., Certo, H.M., Sommer, K., Sather, B.D., Khim, S., Clough, C., Hale, M., Pangallo, J., Ryu, B.Y., et al. (2021). Effective, safe, and sustained correction of murine XLA using a UCOE-BTK promoter-based lentiviral vector. Mol. Ther. Methods Clin. Dev. *20*, 635–651. https://doi.org/10.1016/j.omtm.2021.01.007.

71. Luthers, C.R., Mittelhauser, A., Colamartino, A., Wu, X., Cirigliano, S., Long, J.D., Sanchez, J.M., Romero, Z., and Kohn, D.B. (2025). Hematopoietic stem cell gene therapy for the treatment of X-linked agammaglobulinemia. Mol. Ther. Methods Clin. Dev. *33*, 101555. https://doi.org/10.1016/j.omtm.2025.101555.

72. Kwon, D. (2026). The age of animal experiments is waning. Where will science go next? Nature *650*, 812–814. https://doi.org/10.1038/d41586-026-00563-3.

73. Pike-Overzet, K., Rodijk, M., Ng, Y.Y., Baert, M.R.M., Lagresle-Peyrou, C., Schambach, A., Zhang, F., Hoeben, R.C., Hacein-Bey-Abina, S., Lankester, A.C., et al. (2011). Correction of murine Rag1 deficiency by self-inactivating lentiviral vector-mediated gene transfer. Leukemia *25*, 1471–1483. https://doi.org/10.1038/leu.2011.106.

74. Castiello, M.C., Brandas, C., Ferrari, S., Porcellini, S., Sacchetti, N., Canarutto, D., Draghici, E., Merelli, I., Barcella, M., Pelosi, G., et al. (2024). Exonic knockout and knockin gene editing in hematopoietic stem and progenitor cells rescues RAG1 immunodeficiency. Sci. Transl. Med. *16*, eadh8162. https://doi.org/10.1126/scitranslmed.adh8162.

75. Allen, D., Knop, O., Itkowitz, B., Kalter, N., Rosenberg, M., Iancu, O., Beider, K., Lee, Y.N., Nagler, A., Somech, R., and Hendel, A. (2023). CRISPR-Cas9 engineering of the RAG2 locus via complete coding sequence replacement for therapeutic applications. Nat. Commun. *14*, 6771. https://doi.org/10.1038/s41467-023-42036-5.

76. McAuley, G.E., Yiu, G., Chang, P.C., Newby, G.A., Campo-Fernandez, B., Fitz-Gibbon, S.T., Wu, X., Kang, S.L., Garibay, A., Butler, J., et al. (2023). Human T cell generation is restored in CD3delta severe combined immunodeficiency through adenine base editing. Cell *186*, 1398–1416.e1323. https://doi.org/10.1016/j.cell.2023.02.027.

77. Ott de Bruin, L.M., Bosticardo, M., Barbieri, A., Lin, S.G., Rowe, J.H., Poliani, P.L., Ching, K., Eriksson, D., Landegren, N., Kämpe, O., et al. (2018). Hypomorphic Rag1 mutations alter the preimmune repertoire at early stages of lymphoid development. Blood *132*, 281–292. https://doi.org/10.1182/blood-2017-12-820985.

78. Csomos, K., Ujhazi, B., Blazso, P., Herrera, J.L., Tipton, C.M., Kawai, T., Gordon, S., Ellison, M., Wu, K., Stowell, M., et al. (2022). Partial RAG deficiency in humans induces dysregulated peripheral lymphocyte development and humoral tolerance defect with accumulation of T-bet(+) B cells. Nat. Immunol. *23*, 1256–1272. https://doi.org/10.1038/s41590-022-01271-6.

79. Castiello, M.C., Brandas, C., Capo, V., and Villa, A. (2023). HyperIgE in hypomorphic recombination-activating gene defects. Curr. Opin. Immunol. *80*, 102279. https://doi.org/10.1016/j.coi.2022.102279.

80. Feugier, P., Li, N., Jo, D.Y., Shieh, J.H., MacKenzie, K.L., Lesesve, J.F., Latger-Cannard, V., Bensoussan, D., Crystal, R.G., Rafii, S., et al. (2005). Osteopetrotic mouse stroma with thrombopoietin, c-kit ligand, and flk-2 ligand supports long-term mobilized CD34+ hematopoiesis in vitro. Stem Cells Dev. *14*, 505–516. https://doi.org/10.1089/scd.2005.14.505.

81. Crooks, G.M., Montel-Hagen, A., and Seet, C.S. (2025). Methods or generating T-cells from stem cells and immunotherapeutic methods using the T-cells. https://patents.google.com/patent/WO2017075389A1/en.

82. Nemazee, D. (2017). Mechanisms of central tolerance for B cells. Nat. Rev. Immunol. *17*, 281–294. https://doi.org/10.1038/nri.2017.19.

83. Mesin, L., Ersching, J., and Victora, G.D. (2016). Germinal Center B Cell Dynamics. Immunity *45*, 471–482. https://doi.org/10.1016/j.immuni.2016.09.001.

84. Kinsella, T.M., and Nolan, G.P. (1996). Episomal vectors rapidly and stably produce high-titer recombinant retrovirus. Hum. Gene Ther. *7*, 1405–1413. https://doi.org/10.1089/hum.1996.7.12-1405.

85. Boone, E., Groenen, P.J.T.A., and Langerak, A.W. (2025). PCR GeneScan Analysis of Rearranged Immunoglobulin or T-Cell Receptor Genes for Clonality Diagnostics in Suspect Lymphoproliferations. Methods Mol. Biol. *2865*, 77–102. https://doi.org/10.1007/978-1-0716-4188-0_4.

86. Kitamura, T., Koshino, Y., Shibata, F., Oki, T., Nakajima, H., Nosaka, T., and Kumagai, H. (2003). Retrovirus-mediated gene transfer and expression cloning: powerful tools in functional genomics. Exp. Hematol. *31*, 1007–1014.

87. Wiekmeijer, A.S., Pike-Overzet, K., Brugman, M.H., Salvatori, D.C.F., Egeler, R.M., Bredius, R.G.M., Fibbe, W.E., and Staal, F.J.T. (2014). Sustained Engraftment of Cryopreserved Human Bone Marrow CD34(+) Cells in Young Adult NSG Mice. Biores. Open Access *3*, 110–116. https://doi.org/10.1089/biores.2014.0008.

88. Peperzak, V., Xiao, Y., Veraar, E.A.M., and Borst, J. (2010). CD27 sustains survival of CTLs in virus-infected nonlymphoid tissue in mice by inducing autocrine IL-2 production. J. Clin. Investig. *120*, 168–178. https://doi.org/10.1172/JCI40178.

89. Montel-Hagen, A., Tsai, S., Seet, C.S., and Crooks, G.M. (2022). Generation of Artificial Thymic Organoids from Human and Murine Hematopoietic Stem and Progenitor Cells. Curr. Protoc. *2*, e403. https://doi.org/10.1002/cpz1.403.

90. Unger, P.P.A., Verstegen, N.J.M., Marsman, C., Jorritsma, T., Rispens, T., Ten Brinke, A., and van Ham, S.M. (2021). Minimalistic In Vitro Culture to Drive Human Naive B Cell Differentiation into Antibody-Secreting Cells. Cells *10*, 1183. https://doi.org/10.3390/cells10051183.

91. Zheng, G.X.Y., Terry, J.M., Belgrader, P., Ryvkin, P., Bent, Z.W., Wilson, R., Ziraldo, S.B., Wheeler, T.D., McDermott, G.P., Zhu, J., et al. (2017). Massively parallel digital transcriptional profiling of single cells. Nat. Commun. *8*, 14049. https://doi.org/10.1038/ncomms14049.

92. Hao, Y., Hao, S., Andersen-Nissen, E., Mauck, W.M., 3rd, Zheng, S., Butler, A., Lee, M.J., Wilk, A.J., Darby, C., Zager, M., et al. (2021). Integrated analysis of multimodal single-cell data. Cell *184*, 3573–3587.e29. https://doi.org/10.1016/j.cell.2021.04.048.

93. Germain, P.L., Lun, A., Garcia Meixide, C., Macnair, W., and Robinson, M.D. (2022). Doublet identification in single-cell sequencing data using scDblFinder. F1000Res. *10*, 979. https://doi.org/10.12688/f1000research.73600.2.

94. Korsunsky, I., Millard, N., Fan, J., Slowikowski, K., Zhang, F., Wei, K., Baglaenko, Y., Brenner, M., Loh, P.R., and Raychaudhuri, S. (2019). Fast, sensitive and accurate integration of single-cell data with Harmony. Nat. Methods *16*, 1289–1296. https://doi.org/10.1038/s41592-019-0619-0.

95. Huang, Y., McCarthy, D.J., and Stegle, O. (2019). Vireo: Bayesian demultiplexing of pooled single-cell RNA-seq data without genotype reference. Genome Biol. *20*, 273. https://doi.org/10.1186/s13059-019-1865-2.

96. Borcherding, N., Bormann, N.L., and Kraus, G. (2020). scRepertoire: An R-based toolkit for single-cell immune receptor analysis. F1000Res *9*, 47. https://doi.org/10.12688/f1000research.22139.2.

97. Suo, C., Polanski, K., Dann, E., Lindeboom, R.G.H., Vilarrasa-Blasi, R., Vento-Tormo, R., Haniffa, M., Meyer, K.B., Dratva, L.M., Tuong, Z.K., et al. (2024). Dandelion uses the single-cell adaptive immune receptor repertoire to explore lymphocyte developmental origins. Nat. Biotechnol. *42*, 40–51. https://doi.org/10.1038/s41587-023-01734-7.

98. Garcia-Perez, L., van Eggermond, M.C.J.A., Maietta, E., van der Hoorn, M.L.P., Pike-Overzet, K., and Staal, F.J.T. (2021). A Novel Branched DNA-Based Flowcytometric Method for Single-Cell Characterization of Gene Therapy Products and Expression of Therapeutic Genes. Front. Immunol. *11*, 607991. https://doi.org/10.3389/fimmu.2020.607991.

STAR★METHODS

KEY RESOURCES TABLE

REAGENT or RESOURCE	SOURCE	IDENTIFIER
Antibodies		
CD3 BUV615 (UCHT1)	Waters Biosciences	Cat#612992; RRID:AB_2870263
CD10 BV605 (HI10a)	BioLegend	Cat#312221; RRID:AB_2562156
CD10 APC-Cy7 (HI10a)	BioLegend	Cat#312212; RRID:AB_2146550
CD10 Antibody, anti-human, REAfinity Biotin (REA877)	Miltenyi Biotec	Cat#130-114-500; RRID:AB_2726670
CD19 BV421 (HIB19)	BioLegend	Cat#302234; RRID:AB_11142678
CD19 AF647 (HIB19)	BioLegend	Cat#363040; RRID:AB_2750324
CD19 BUV563 (SJ25C1)	Waters Biosciences	Cat#612917; RRID:AB_2870202
CD19 PE (HIB19)	BioLegend	Cat# 302208; RRID:AB_314238
CD20 PE-CF594 (2H7)	Waters Biosciences	Cat#562295; RRID:AB_11153322
CD27 APC-Fire810 (QA17A18)	BioLegend	Cat#393214; RRID:AB_2860962
CD33 BV785 (WM53)	BioLegend	Cat#303428; RRID:AB_2650888
CD34 PE-CF594 (581)	Waters Biosciences	Cat#562383; RRID:AB_11154586
CD38 PE-Cy7 (HIT2)	BioLegend	Cat#303516; RRID:AB_2072782
CD38 PE-Fire810 (S17015F)	BioLegend	Cat#397225; RRID:AB_2894562
CD45 BV650 (HI30)	Waters Biosciences	Cat#563717; RRID:AB_2738387
CD45 Spark PLUS UV395 (HI30)	BioLegend	Cat#304096; RRID:AB_3097561
CD45 BUV805 (HI30)	Waters Biosciences	Cat#612891; RRID:AB_2870179
CD56 PE-Cy5 (B159)	Waters Biosciences	Cat#555517; RRID:AB_395907
CD79A PerCP-Cy5.5 (HM47)	BioLegend	Cat#333508; RRID:AB_2075752
CD117 PE-Vio770 (REA787)	Miltenyi Biotec	Cat#130-111-594; RRID:AB_2654583
CD154 PE-Cy7 (24-31)	BioLegend	Cat#310831; RRID:AB_2563016
CD179A PE (HSL96)	BioLegend	Cat#347404; RRID:AB_2216935
BLIMP-1 PE (6D3)	Waters Biosciences	Cat#564702; RRID:AB_2738901
DNTT APC (E17-1519)	Waters Biosciences	Cat#332791; RRID:AB_2868638
IgA PerCP-Vio700 (IS11-8E10)	Miltenyi Biotec	Cat#130-113-478; RRID:AB_2733052
IgD APC (IA6-2)	Waters Biosciences	Cat#561303; RRID:AB_10642578
IgD FITC (IA6-2)	BioLegend	Cat#348206; RRID:AB_10612567
IgG BV786 (G18-145)	Waters Biosciences	Cat#564230; RRID:AB_2738684
IgM BV510 (MHM-88)	BioLegend	Cat#314522; RRID:AB_2562916
IgM BV650 (MHM-88)	BioLegend	Cat#314525; RRID:AB_2563382
Pax5 AF647 (1H9)	BioLegend	Cat#649703; RRID:AB_2562424
mouse CD45-APC-Fire810 (30-F11)	BioLegend	Cat#103173; RRID:AB_2860599
Goat F(ab')2 Anti-Human IgM-LE/AF	Sanbio	Cat#2022-14
Recombinant Human ICAM-1-Fc Chimera (carrier-free)	BioLegend	Cat#552906
Bacterial and virus strains		
pCCL.MND.coRAG1 lentivirus	Batavia	Cat#18D008
Biological samples		
Umbilical cord blood	LUMC	N/A
Mobilized peripheral blood	LUMC	N/A
RAG1-SCID patient $CD34^+$ HSPCs	LUMC	N/A
Humanized mouse bone marrow	LUMC	N/A

(*Continued on next page*)

Continued

REAGENT or RESOURCE	SOURCE	IDENTIFIER
Chemicals, peptides, and recombinant proteins		
FMS-like tyrosine kinase 3 ligand, research grade	Miltenyi Biotec	Cat#130-096-474
Stem cell factor, research grade	Miltenyi Biotec	Cat#130-096-692
Interleukin 3, research grade	Miltenyi Biotec	Cat#130-093-909
Interleukin 4, research grade	Miltenyi Biotec	Cat#130-093-917
Interleukin 6, research grade	Miltenyi Biotec	Cat130-093-929
Interleukin 7, research grade	Miltenyi Biotec	Cat#130-095-367
Interleukin 21, research grade	Miltenyi Biotec	Cat#130-095-767
Iscove's Modified Dulbecco's Medium (IMDM) with L Glutamine and HEPES	Gibco	Cat#12-726F
Iscove's Modified Dulbecco's Medium (IMDM), no phenol red	Gibco	Cat#21056023
Dulbecco's Modified Eagle Medium (DMEM), high glucose, pyruvate	Thermo Fisher Scientific	Cat#41966052
Roswell Park Memorial Institute (RPMI) 1640 medium, Dutch Modified	Gibco	Cat#22409-015
Cellgenix SCGM medium w/o phenolred	Sartorius CellGenix GmbH	Cat#20806-0500
Pen/Strep 100x (10,000 U Pen/mL & 10,000 μg Strep/mL)	Corning	Cat#30-002-CI
Heat-inactivated fetal calf serum (FCS-HI)	Bodinco	Cat#BDCSOOFD1
GlutaMax (200 mM)	Gibco	Cat#35050-038
Non-essential amino-acids (200 mM)	Gibco	Cat# 11140-050
Sodium pyruvate (100 mM)	Gibco	Cat# 11360-070
L-ascorbic acid 2-phosphate	Sigma	Cat#A8960-5G
Human insulin solution (10 mg/mL)	Sigma-Aldrich	Cat# 11061-68-0
Human transferrin solution (10 mg/mL)	Lonza	Cat# CC-4205
Dimethyl sulfoxide (DMSO)	VWR international	Cat# 23.500.260
0,05% Trypsin-EDTA (1X)	Gibco	Cat#25300-062
PBS, pH 7.4	Fresenius Kabi	Cat#M090001/02
0.5 M EDTA pH 8.0 UltraPure	Life Tech	Cat#15575-038
Bovine serum albumin (BSA)	Sigma-Aldrich	Cat#A9647-100G
Sodium azide	Pharmacy AZL	Cat# 97936383
Polyethylenimine, Linear, MW 25000, Transfection Grade (PEI 25K)	Polysciences	Cat#23966-100
Collagenase A (2 mg/mL)	Roche	Cat#10103586001
Brilliant stain buffer	Waters Biosciences	Cat#563794
BD Pharmingen™ 7-AAD	Waters Biosciences	Cat#559925
True-Stain Monocyte Blocker™	BioLegend	Cat#426102
Zombie NIR™ Fixable Viability Kit	BioLegend	Cat#423106
Fluo-4, AM, cell permeant	Invitrogen	Cat#F14201
Q5® High-Fidelity DNA Polymerase	New England Biolabs	Cat#M0491S
BamHI-HF	New England Biolabs	Cat#R3136S
NotI-HF	New England Biolabs	Cat#R3189S
Lentiboost -P Pharma grade	Sirion	Cat#SB-A-LF-901-01
AmpliTaq Gold polymerase	Thermo Fisher Scientific	Cat#N8080241
GeneScan™ 500 ROX™ dye Size Standard	Thermo Fisher Scientific	Cat# 401734
Critical commercial assays		
Erythrocyte Sedimentation kit II, human	Miltenyi Biotec	Cat#130-132-321
CD34 Microbead Kit UltraPure, human	Miltenyi Biotec	Cat#130-100-453

(*Continued on next page*)

Continued

REAGENT or RESOURCE	SOURCE	IDENTIFIER
LEGENDplex^{TM} Human Immunoglobulin Isotyping Panel (6-plex) with V-bottom Plate	Biolegend	Cat#740640
PrimeFlow^{TM} RNA Assay Kit	Thermo Fisher Scientific	Cat#88-18005-210
QIAamp DNA Micro Kit (50)	QIAGEN	Cat#56304
RNeasy Micro Kit (50)	QIAGEN	Cat#74004
TaqMan^{TM} Fast Advanced Master Mix for qPCR	Thermo Fisher Scientific	Cat#4444964
Streptavidin MicroBeads	Miltenyi Biotec	Cat#130-048-101
Anti-PE MultiSort Kit	Miltenyi Biotec	Cat#130-090-757
eBioscience^{TM} Foxp3 / Transcription Factor Staining Buffer Set	Thermo Fisher Scientific	Cat#00-5523-00
**Deposited data**		
scRNA-seq analysis of B-cell development in ABOs	This study	EGA: EGAD50000002433
Existing scRNA-seq data sets used for analyses	Kaiser et al.[4] Zeng *et al.*[34]	GEO: GSE214693; GEO: GSE289435
**Experimental models: Cell lines**		
MS-5	DSMZ	Cat#ACC 441; RRID:CVCL_2128
Phoenix-Eco	Kinsella *et al.*[84]	N/A
**Experimental models: Organisms/strains**		
NOD.Cq-Prkdc^{scid}Il2rg^{tm1Wjl}/SzJ (NSG) mice	Charles River Laboratories	RRID:IMSR_JAX:005557
**Oligonucleotides**		
hCD40L (forward) 5’-TAAGCAGGATCCATGAT CGAAACATACAACCAAAC-3’	IDT	N/A
hCD40L (reverse) 5’-TAAGCAGCGGCCGCTCA GAGTTTGAGTAAGCCAAAGG-3’	IDT	N/A
ALB (forward) 5’-GCTGCTATCTCTTGTGGGCTGT-3’	Sigma-Aldrich	N/A
ALB (reverse) 5’-ACTCATGGGAGCTGCTGGTTC-3’	Sigma-Aldrich	N/A
ALB (probe) 5’-VIC-CCTGTCATGCCCACACAAA TCTCTCC-TAMRA-3’	Sigma-Aldrich	N/A
HIV-1 ψ (forward) 5’-CAGGACTCGGCTTGCTGAAG-3’	Sigma-Aldrich	N/A
HIV-1 ψ (reverse) 5’-TCCCCCGCTTAATACTGACG-3’	Sigma-Aldrich	N/A
HIV-1 ψ (probe) 5’-FAM-CGCACGGCAAGAGGCGAGG-TAMRA-3’	Sigma-Aldrich	N/A
Hs99999905_m1 Hu GAPDH (FAM-MGB)	Thermo Fisher Scientific	Cat#4331182
Hs00757808_m1 Hu *AICDA* (FAM-MGB)	Thermo Fisher Scientific	Cat#4331182
Hs00153357_m1 Hu *PRDM1* (FAM-MGB)	Thermo Fisher Scientific	Cat#4331182
Hs00172003_m1 Hu *PAX5* (FAM-MGB)	Thermo Fisher Scientific	Cat#4331182
coRAG1 PrimeFlow probe set	Thermo Fisher Scientific	Custom order
Human RPL13A PrimeFlow probe set	Thermo Fisher Scientific	Cat#VA4-13187
EuroClonality/BIOMED-2 primer mixes	Boone *et al.*[85]	N/A
**Recombinant DNA**		
pMX-IRES-GFP	Kitamura *et al.*[86]	N/A
pCL-Eco	Addgene	Cat#12371; RRID:Addgene_12371
**Software and algorithms**		
FlowJo software (version 10.10.0)	Waters Biosciences	RRID:SCR_008520
OMIQ software	Dotmatics	RRID:SCR_027879
Infinicyt software (version 2.0.6.b.023)	Cytognos	RRID:SCR_026033
LEGENDplex Data Analysis Software Suite	BioLegend	N/A
Quantstudio Design & Analysis software (version 1.5.2)	Applied Biosystems	N/A

(*Continued on next page*)

Continued

REAGENT or RESOURCE	SOURCE	IDENTIFIER
GeneMarker® software (version 3.0.0)	Thermo Fisher Scientific	RRID:SCR_015661
Other		
LD columns	Miltenyi Biotec	Cat#130-042-901
LS columns	Miltenyi Biotec	Cat#130-042-401
Pre-Separation filters (30 μm)	Miltenyi Biotec	Cat#130-041-407
MACS MultiStand	Miltenyi Biotec	Cat#130-042-303
QuadroMACS Separator	Miltenyi Biotec	Cat#130-090-976
Cell Culture Inserts	Millipore	Cat#PICM0RG50
5 mL Polystyrene Round-Bottom Tube with Cell-strainer Cap	Falcon	Cat#352235
Cell Strainer 70 μm Nylon	Falcon	Cat#352350
BD FACSAria III Cell Sorter	Waters Biosciences	RRID:SCR_016695
BD FACSCanto II Flow Cytometry System	Waters Biosciences	RRID:SCR_018056
BD LSRFortessa X-20 Cell Analyzer	Waters Biosciences	RRID:SCR_025285
Cytek Aurora Spectral Analyzer 5 laser	Cytek Biosciences	RRID:SCR_019826
QuantStudio™ 3 Real-Time PCR System, 96-well, 0.2 mL, laptop	Applied Biosystems	Cat#A28567; RRID:SCR_018712

EXPERIMENTAL MODEL AND STUDY PARTICIPANTS DETAILS

Human study and ethics statement

Umbilical cord blood (UCB) samples were collected post-delivery following written informed consent from the donors in accordance with protocols approved by the Institutional Medical Ethics Committee of Leiden University Medical Center (LUMC) (14.078). All samples were obtained from healthy births according to institutional eligibility criteria.

Mobilized peripheral blood (mPB) from healthy controls and patients with RAG1-SCID were mobilized using G-CSF and plerixafor and collected using leukapheresis as per our clinical trial protocol (NCT04797260). $CD34^+$ cells from patients with RAG1-SCID (Table S1) were enriched from mPB using the CliniMACS system (Miltenyi). The patients' parents or legal guardians provided informed consent to use leftover HSPCs for research purposes in accordance with the Declaration of Helsinki and the Leiden University Medical Center Institutional Review Board.

UCB- and healthy donor mPB-derived cells used in this study were obtained from anonymized donors. Information regarding biological sex, gestational age, race, ethnicity, ancestry, and socioeconomic status was not available for the samples analyzed. The absence of sex- and ancestry-related data is a limitation regarding the generalizability of the findings. The number of independent UCB and mPB donors are indicated in the corresponding figure legends.

Animal studies and ethics

NOD.Cq-Prkdc^{scid}Il2rg^{tm1Wjl}/SzJ (NSG) mice (strain 1327; originally obtained from Charles River Laboratories) were bred and maintained under specific pathogen-free (SPF) conditions in the animal facility of the LUMC. All animal procedures were approved by the LUMC Institutional Ethical Committee on Animal Experiments and conducted in accordance with institutional and national regulations governing animal experimentation.

Female mice aged 3.5–5.5 weeks were used for humanization experiments.[87] Mice were sublethally irradiated (1.91 cGy, orthovoltage X-rays) and, within 24 h, transplanted intravenously with $1.5x10^5$ human UCB-derived $CD34^+$ hematopoietic stem and progenitor cells (HSPCs) in 100 μL PBS. Prior to transplantation, $CD34^+$ HSPCs were cultured for 2 days in StemSpan-SFEM (StemCell Technologies) medium supplemented with antibiotics (penicillin 100 IU/mL and streptomycin 100 μg/mL (Gibco)), recombinant human stem cell factor (SCF) (100 ng/mL), FMS-like tyrosine kinase 3 ligand (FLT3L) (50 ng/mL), and thrombopoietin (TPO) (10 ng/mL) (all Miltenyi BioTec).

For the first 4 weeks post-transplantation, mice received drinking water containing polymyxin B (0.07 mg/mL), ciprofloxacin (0.0875 mg/mL), and amphotericin B (0.1 mg/mL), along with *ad libitum* food pellets and DietGel Recovery (Clear H2O). Thereafter, mice were maintained on regular chow and water *ad libitum*.

At 17 weeks, mice were euthanized by CO_2 inhalation, and spleen, femurs, and tibiae were collected. BM was flushed from femurs and tibiae using Iscove's Modified dulbecco's Medium (IMDM HEPES-buffered; Lonza) supplemented with 2.5% heat-inactivated fetal calf serum (FCS-HI; Bodinco) and antibiotics (penicillin 100 IU/mL and streptomycin 100 μg/mL). Spleens were mechanically dissociated through 70 μm nylon strainers (BD Falcon). Harvested cells were cryopreserved in FCS containing 10% DMSO and

stored in liquid nitrogen until use for flow cytometric analysis. Randomization was not applicable to the current study design as BM samples constituted a single experimental group in *ex vivo* analyses.

Cell lines

MS-5 (DSMZ) mouse bone marrow (BM) stromal cells were cultured in IMDM supplemented with 10% FCS-HI and antibiotics (penicillin 100 IU/mL and streptomycin 100 μg/mL). CD40L-expressing MS-5 cells were generated by retroviral transduction. Full-length human CD40L cDNA was PCR-amplified using Q5 HiFi polymerase (New England Biolabs) (primers listed in key resources table) and cloned into the pMX-IRES-GFP vector[86] using BamHI/NotI restriction sites (BamHI-HF and NotI-HF, New England Biolabs). The construct was sequence-verified. Retrovirus was produced by co-transfecting pMX-hCD40L-IRES-GFP and pCL-Eco into Phoenix-Eco packaging cells[84] using polyethylenimine (Polysciences).[88] Phoenix-Eco cells were maintained in Dulbecco's Modified Eagle Medium (DMEM) with 10% FCS-HI and antibiotics (penicillin 100 IU/mL and streptomycin 100 μg/mL). Viral supernatant was collected 48 h post-transfection and applied (1:1) to MS-5 cells ($1.0x10^5$) in 6-well plates. MS5-CD40L cells were sorted on high expression of GFP and expression of CD40L after staining with CD154 PE-Cy7 (clone 24–31, Biolegend) on a CytoFLEX SRT cell sorter (Beckman Coulter). Cell identity was confirmed by morphology during routine culture and flow cytometric analysis for CD40L expression. The cell lines used in this study were tested for mycoplasma contamination by PCR analysis prior to freezing and subsequent use in experiments.

METHOD DETAILS

Hematopoietic stem and progenitor cell isolation

Leukocytes from UCB and mPB were first purified using the Erythrocyte Sedimentation Kit II (Miltenyi), followed by $CD34^+$ cell enrichment with the CD34 MicroBead Kit UltraPure (Miltenyi) per manufacturer's instructions.

For depletion of lymphoid precursor cells, UCB leukocytes were labeled with a cocktail of biotinylated lineage-specific (Lin) antibodies and CD10-biotin antibodies (Miltenyi, clone REA877/97C5, 1 μL/107 cells) for 10 min at 4°C. Cells were washed in PBS containing 0.5% BSA and 2 mM EDTA, labeled with anti-biotin MicroBeads for 15 min at 4°C, and passed through LD columns (Miltenyi) for negative selection of Lin^-CD10^-cells. These cells were subsequently labeled with CD34 MicroBeads for 30 min at 4°C and subjected to positive selection using LS columns (Miltenyi) to obtain $CD34^+Lin^-CD10^-$cells. All HSPCs were cryopreserved in FCS with 10% DMSO and stored in liquid nitrogen until further use.

B cell development cultures

B-cell development cultures were performed under ABO, MS-5 monolayer, or stromal-free conditions. To minimize batch-to-batch variability in stromal support, the same cryopreserved batch of MS-5 cells was used throughout all experiments. MS-5 cells were thawed and passaged at least twice before coculture initiation, and only cells up to passage 10 after thawing were used for HSPC differentiation cultures. For ABO and monolayer cultures, MS-5 cells were harvested with 0.05% Trypsin-EDTA (Gibco) and resuspended in IMDM supplemented with 10% FCS-HI, antibiotics (penicillin 100 IU/mL and streptomycin 100 μg/mL), 2 mM GlutaMAX, 2 mM non-essential amino acids, 1 mM sodium pyruvate (all Gibco), 30 μM L-ascorbic acid 2-phosphate (Sigma-Aldrich), 1 μg/mL insulin (Sigma-Aldrich), and 2.5 μg/mL transferrin (Lonza), referred to as ABO medium.

ABO assembly – Following the ATO protocol,[89] $CD34^+$ cells ($7.5x10^3$) were mixed with MS-5 cells ($1.5x10^5$) per ABO in 1.5 mL Eppendorf tubes. Cells were centrifuged at 300x*g* for 5 min (RT), and resuspended in 5 μL ABO medium per ABO. Droplets (5 μL) were placed on 0.4 μm Millicell culture inserts (EMD Millipore) in 6-well plates. B cell development was induced by culturing ABOs with 1 mL ABO medium with FLT3L and SCF supplemented with IL-6 for 7 days (day 0–7), followed by FLT3L and SCF supplemented with IL-7 (5 ng/mL each) for 7 days (day 7–14). ABOs using mPB-derived $CD34^+$ cells were supplemented with FLT3L, SCF and IL-7 for 14 days. ABOs were subsequently maintained in cytokine-free ABO medium for 21–28 days (Figure 1A). Medium was refreshed every 2–3 days by aspirating the lower compartment and replacing with 800 μL fresh ABO medium.

2D monolayer and stromal-free cultures – $CD34^+$ cells ($1.0x10^5$) were seeded onto either an MS-5 cell monolayer or ICAM-1-Fc-coated wells (5 μg/mL; BioLegend, coated overnight) in 24-well plates. B cell development was induced in ABO medium with FLT3L and SCF supplemented with IL-6 for 7 days, followed by FLT3L and SCF (25 ng/mL each) supplemented with IL-7 (20 ng/mL) for 7 days, and subsequent cytokine-free maintenance for 21 days. Medium was completely refreshed every 2–3 days. In MS-5 monolayer cultures, non-adherent cells (hematopoietic cells) were transferred onto fresh MS-5 layers every 7–10 days.

B-cell activation cultures

On day 35, ABOs were harvested into 200 μL RPMI1640 (Gibco) containing 2 mg/mL collagenase A (Roche) and incubated for 20 min at 37°C with continuous shaking at 600 rpm. Cell aggregates were dissociated by pipetting, and single-cell suspensions were obtained by passing cells through 35 μm strainers (Falcon). ABO-derived B cells were enriched by MACS: cells were stained with CD19-PE antibodies (BioLegend, clone HIB19; 2.5 μL/10^7 cells) in PBS containing 0.5% BSA and 2 mM EDTA for 15 min at 4°C, washed, labeled with anti-PE MultiSort MicroBeads (Miltenyi) for 15 min at 4°C, and positively selected using LS columns. MicroBeads were removed per manufacturer's instructions. About $3.5x10^5$ $CD19^+$ cells can be extracted per ABO. For B-cell activation, ABO-$CD19^+$ cells were mixed with CD40L-expressing MS-5 cells in an ABO configuration as described above and cultured for

11 days. Cultures were maintained in basic ABO medium supplemented with IL-21 (50 ng/mL) and IL-4 (100 ng/mL) (both from Miltenyi) for 11 days[90] as described above. Medium was refreshed every 2–3 days by aspirating the lower compartment and replacing with 800 μL fresh ABO medium supplemented with aforementioned cytokines.

Flow cytometry

Cells were collected in PBS containing 0.2% BSA and 0.1% sodium azide (FACS buffer). Surface staining was performed in FACS buffer supplemented with Brilliant stain buffer (BD Biosciences) and Monocyte blocker (BioLegend) for 30 min at 4°C. The following monoclonal antibodies were used: CD10-BV605, CD10-APC-Cy7 (both HI10a), CD19-BV421 (HIB19), CD19-AF647 (SJ25C1), CD27-APC-Fire810 (QA17A18), CD33-BV785 (WM53), CD38-PE-Cy7 (HIT2), CD38-PE-Fire810 (S17015F), CD45-Spark PLUS UV395 (HI30), mouse CD45-APC-Fire810 (30-F11), IgD-FITC (IA6-2), IgM-BV510 (MHM-88) (all BioLegend), CD3-BUV615 (UCHT1), CD20-PE-CF594 (2H7), CD34-PE-CF594 (581), CD45-BV650 (HI30), CD45-BUV805 (HI30), CD56-PE-Cy5 (B159), IgD-APC (IA6-2), IgG-BV786 (G18-145) (all Waters Biosciences), CD117-PE-Vio770 (REA787), and IgA-PerCP-Vio700 (IS11-8E10) (both Miltenyi Biotec) (see key resources table).

For intracellular staining, cells were fixed and permeabilized for 45 min at RT using the eBioscience FoxP3/Transcription Factor Staining Buffer Set (Thermo Fisher) and subsequently stained using CD79A-PerCP-Cy5.5 (HM47), CD179A-PE (HSL96), IgM-BV650 (MHM-88), PAX5-AF647 (1H9) (all BioLegend) and DNTT-APC (Waters Biosciences, E17-1519) monoclonal antibodies (see key resources table) for 30 min at RT. Zombie-NIR viability dye (Biolegend) was used to exclude dead cells.

Data acquisition was performed on a Cytek Aurora 5L flow cytometer (Cytek Biosciences). Flow cytometric data were analyzed using FlowJo software (version 10.10.0). Optimized t-SNE (opt-SNE) dimensionality reduction was carried out in OMIQ (Dotmatics), and trajectory analysis was performed using Infinicyt software (version 2.0.6.b.023; Cytognos). Complete gating strategies for each experiment are provided in the Supplemental Information (see Document S1).

Ca^{2+} flux measurements

Ca^{2+} flux in ABO-CD19$^+$ cells was measured using the cell-permeant dye Fluo-4 AM (Invitrogen). A total of 5.0x10^5 ABO-CD19$^+$ cells were collected and washed twice in IMDM without phenol red (Gibco) supplemented with 1% FCS-HI. Cells were stained in 400 μL IMDM without phenol red containing 1% FCS-HI and Fluo-4 AM (2 μg/mL) for 60 min at 37°C protected from light. After staining, cells were washed twice with IMDM without phenol red supplemented with 1% FCS-HI and resuspended in 400 μL of the same medium. Ca^{2+} flux was measured on a BD LSRFortessa X-20 Cell Analyzer (Waters Biosciences). Baseline fluorescence was recorded for 1 min at 37°C. Subsequently, 20 μL goat F(ab')$_2$ anti-human IgM-LE/AF antibodies (Sanbio) were added to the cells, vortexed briefly, and fluorescence acquisition was continued for an additional 4 min at 37°C.

Antibody production measurements

Antibody production was quantified using the Human Ig Isotyping Panel LEGENDplex (6-plex; BioLegend) according to the manufacturer's instructions. On day 10 of B-cell activation cultures, the medium was refreshed, and supernatants were collected after 24 h for antibody production measurement. Samples were acquired on a BD FACSCanto II flow cytometer (Waters Biosciences), and data were analyzed using the LEGENDplex Data Analysis Software Suite (BioLegend). Background signals from medium-only controls were subtracted from the sample readouts.

Sample and library preparation for scRNA-seq and scBCR-seq

Day 21, 28, and 35 ABOs using UCB-derived CD34$^+$ HSPCs from 4 donors were used for scRNA-seq analysis. To this end, ABOs were harvested as described above and collected in PBS containing 0.5% BSA and 2 mM EDTA. Cells were stained using CD10-APC-Cy7 (HI10a), CD33-BV785 (WM53) and CD38-PE-Fire810 (S17015F) monoclonal antibodies (all from BioLegend, see key resources table) in PBS containing 0.5% BSA, 2 mM EDTA, Brilliant Stain buffer and Monocyte blocker for 30 min at 4°C. Dead cells were excluded using 7-AAD (Waters Biosciences). Cells were sorted based on a CD10$^+$CD38$^+$CD33$^-$ phenotype using a BD FACSAria III 4L Cell Sorter (Waters Biosciences) (Figure S3A). Cells (>80% viable) from individual donors at days 21, 28 and 35 of cultures were pooled (1:1:1:1) prior to library preparation. Cell suspensions were processed at the Leiden Genome Technology Center (LGTC) and partitioned on the 10x Genomics Chromium platform using GEM-X Single Cell 5′ v3 and GEM-X Single Cell V(D)J v3 for BCR profiling.[91] For each timepoint, paired libraries were constructed: a 5′ GEX library and a V(D)J-B library. Per reaction 3.5–4x10^5 cells were loaded, aiming for 6x10^4 cells per reaction. Pooled libraries were sequenced on an Illumina NovaSeq X sequencer. We targeted 50,000 read pairs per cell for GEX and 5,000 read pairs per cell for V(D)J, resulting in three datasets. GEX data were processed with Cell Ranger 9.0.1 multi and mapped to GRCh38-2024-A for GEX data and to vdj_GRCh38_alts_ensembl-7.1.0 for the B cell receptor data. Final realized coverage per dataset was ~30,000 reads/cell (GEX) and ~7,000 reads/cell (V(D)J).

Processing of scRNA-seq data

Gene count matrices were loaded into R (v4.3.2) using the Read10X function from the Seurat package (v5.0.1).[92] All TCR and BCR related genes were removed from the original count matrix to prevent clustering biased toward the TCR or BCR repertoire in further

downstream analyses. Seurat objects were created for every dataset including cells with >500 genes expressed and excluding genes expressed in fewer than 3 cells. The PercentageFeatureSet function was used to calculate the percentage of mitochondrial gene expression per cell.

For every individual dataset, the Vireo method was applied based on genotyping to identify the donors *in silico* for every cell and used the genotyping profiles to detect doublets. In addition to doublets detected by genotype, scDblFinder (https://github.com/plger/scDblFinder)[93] was used to detect additional doublets in our data. The identified doublets were used for model training for scDblFinder by providing the barcodes of genotyped detected doublet cells using the knownDoublets and knownUse arguments. The expected doublet rate, i.e., the proportion of the cells expected to be doublets, was set to 0.008 (0.8%) based on the doublet rates 10x Genomics expects for 5′ sequencing libraries. Detected doublets were subsequently removed from data together with low-quality cells (cells with <1000 UMI counts and >10% mitochondrial gene expression) and cells without a detected genotype.

The three individually processed and integrated Seurat objects from day 21–35 ABOs were merged into a single dataset. Prior to integration, the merged Seurat object was prepared for batch correction using the Seurat v5 integration workflow. The data was split into four layers based on the four identified genotypes. The data layers subsequently were normalized, variable features were identified, and the data was scaled while regressing out cell cycle effects. Principal Component Analysis (PCA) was run subsequently.

The Harmony integration algorithm[94] was applied to correct for donor effects within the three experimental runs, while preserving biological variation. Data layers for every genotype were processed using the IntegrateLayers function with the HarmonyIntegration method in Seurat (v5.0.1). Harmony iteratively adjusts the PCA embedding to ensure that cells from different batches are merged in local neighborhoods, resulting in a single, harmonized low-dimensional space containing 109,227 cells. This corrected embedding (integrated.harmony) was then used for downstream clustering and UMAP visualization. After integration analysis, clustering analysis was performed with the FindNeighbours (dims 1:40) and FindClusters function (resolution = 1) which identified 32 clusters. Cells were visualized using a two-dimensional UMAP plot generated using the RunUMAP function in Seurat package with 50 principal components. After scaling the RNA data, the FindAllMarkers function in Seurat was used to identify differentially expressed genes between the 32 clusters which were used for cell type annotation.

Finally, all non-B cell clusters from the dataset based on cell type annotation, as well as clusters representing low-quality cells were removed from the data analysis. The criteria for low-quality clusters were determined based on their distinct transcriptional profiles and/or their metadata attributes, such as high percentages of mitochondrial gene expression and low percentages of ribosomal gene expression. Based on these criteria, 15 clusters were removed from the dataset. The remaining cells were retained for downstream analyses, which resulted in a dataset consisting of 89,815 cells. Upon reclustering of the data, the counts for TCR and BCR genes were included in the Seurat object.

scRNA-seq data analysis

Cell type annotation – Cell type annotation was performed using a manual and automated approach. Manual annotation[4,34] was done using the FindAllMarkers function in Seurat to identify differentially expressed genes between the 32 clusters. Automated cell cluster annotation was performed using Seurat’s Azimuth tool in Seurat version 5. Processed single-cell RNA sequencing data were separately integrated with a reference atlas of human bone marrow cells[34] using the Azimuth pipeline. This integration involved the transfer of cell type labels from the reference dataset to the query dataset through canonical correlation analysis (CCA) and *k*-nearest neighbor (kNN) mapping. To refine these annotations, manual curation was performed using the cluster marker genes identified by Seurat to align with biologically relevant markers reported in published studies4. The final cell type annotations represent a combination of automated labeling from Azimuth and manual verification using validated marker genes.

Data demultiplexing – The Bayesian demultiplexing tool Vireo (v 0.4.2, R version)[95] was used to determine the identity of the individual donor for every cell. In brief, a list of single nucleotide polymorphism (SNP) positions was first generated by aligning all expressed reads from each cell and selecting the positions with a minimum allele frequency of 0.1 and minimum total coverage of 20. Next, overlapping SNPs were identified in each cell and at each position, which were counted in two disjoint groups corresponding to the reference and non-reference alleles. The allelic count matrices were then used to fit a Vireo model that either identified the most likely donor for each cell or classified the cell as a doublet.

Data visualization – The Loupe Browser (version 8.1.2) was used to visualize the Seurat objects and create UMAP plots, gene expression and gene signature plots. Dotplots and violinplots were created using the Dotplot() and VlnPlot() functions in Seurat.

scBCR repertoire analysis

Clonality analysis – The all_contig_annotations.csv files from the ABO and healthy BM data were used as input for BCR clonality analysis by scRepertoire2.[96] The median number of reads per contig was 851 for *IGH* and 1,578 for *IGK*/*IGL*, with corresponding median UMI counts of 44 and 87, respectively. The slightly higher values for light chains are expected given their shorter and less complex loci. Clonotypes were defined based on the complementarity-determining region 3 (CDR3) sequences. Clones were identified using the strict criterion of shared CDR3 nucleotide sequences and V-gene usage, with a normalized Levenshtein edit distance threshold of 0.85. The combineBCR() function was used to pair single-cell barcodes with corresponding sequences. The percentage of unique clones per cluster was calculated using the clonalQuant() function. The clonalAbundance() function was used to calculate the abundance of clones per cluster to investigate clonal enrichment per cluster. Different diversity metrices were calculated using clonalDiversity(): (1) Shannon entropy to evaluate the richness and evenness of the clonotype in one score, (2) Inverse Simpson’s diversity

index (>20 = high clonal diversity), (3) Normalized Entropy (0–1 with 1 = high entropy), (4) Gini-Simpson index (0–1 with 1 = high diversity). Length distributions for CDR3 sequences were calculated using the clonalLength() function for both light and heavy chains using the amino acid length of the CDR3 region.

To investigate overall clonal similarity between donors, the Morisita index was computed using clonalOverlap(). Clonotype changes between donors were visualized by the clonalCompare() function with the clones called by amino acid sequence of the CDR3 region.

Ig heavy and light chain rearrangements – Raw reads from the gene expression sequencing runs were processed using cellranger_vdj in Cell Ranger (v.9.0.1) with a custom reference provided by the manufacturer (version 2.0.0 GRCh38 VDJ-alts-ensembl). We separately ran cellranger_vdj for BCR reconstruction using the '–chain = IG' argument. Next, BCR contigs contained in all_contigs.fasta and all_contig_annotations.csv were processed further using dandelion singularity container (v.0.2.4) (https://www.github.com/zktuong/dandelion). BCRs were subsequently matched to cell barcodes with dandelion.[97] Dandelion identified BCR rearrangements in 49,621 cells, with combined heavy and light chain rearrangements in 12,596 cells (7,540 cells with rearranged *IGH* and *IGK* loci, 5,056 cells with rearranged *IGH* and *IGL* loci).

Data visualization – To visualize the clonal dominance within different B-cell populations, clonotype frequencies were calculated and plotted as pie charts. A separate healthy BM control cohort was used as reference (BM mononuclear cells human (5′ HT, v2.0), Universal 5′ Gene Expression dataset analyzed using Cell Ranger 6.1.0, 10x Genomics; 2021, August 23). For each dataset, clonotype frequencies were determined at three levels: IGH Clonotypes; Based on unique heavy chain CDR3 amino acid sequences (cdr3_aa1). Light Chain Clonotypes; Based on unique light chain (*IGK*/*IGL*) CDR3 amino acid sequences (cdr3_aa2). Paired Strict Clonotypes; Based on unique, paired heavy and light chain V-gene combinations and identical CDR3 lengths (CTstrict). For the strict clonotype analysis, only clonotypes with a successfully paired heavy and light chain were included. For each of the three levels, the occurrences of every unique clonotype were counted across all cells in the respective dataset. To highlight the most expanded clones, the top 200 most frequent clonotypes were selected for visualization. Clonotype analysis visualizations were created using the plot functions within scRepertoire2. Additional boxplots, proportion plots and density plots and pie charts were generated using ggplot2 in R.

Heatmaps illustrating V- and J gene segment use for *IGH*, *IGK* and *IGL* loci were generated by calculating *z*-scores based on the percentage of gene segment usage. Heatmaps were generated in GraphPad Prism (version 9.3.1).

LV transduction of HSPCs derived from patients with RAG1-SCID

$CD34^+$ HSPCs derived from mPB of patients with RAG1-SCID (Table S1) ($1.0x10^6$ cell/mL) were cultured for 24 h in SCGM (CellGenix) supplemented with antibiotics (penicillin 100 IU/mL and streptomycin 100 μg/mL), FLT3L, SCF (both 300 ng/mL), TPO (100 ng/mL) and IL-3 (10 ng/mL) (Miltenyi) prior to LV transduction. HSPCs were transduced by spinoculation for 60 min at 800x*g* and 32°C with the pCCL.MND.coRAG1 LV vector (1000 viral particles/cell; Batavia Biosciences) in the presence of 1 mg/mL LentiBOOST P Pharma grade Sirion (Bio-Connect). After overnight culture, culture medium was replaced with ABO medium supplemented with FLT3L, SCF and IL-7 (each 25 ng/mL) and maintained for 24 h prior to ABO assembly. ABOs were cultured for 2 weeks in ABO medium containing FLT3L, SCF and IL-7 (each 25 ng/mL) followed by cytokine-free medium from day 14 onward.

Quantitative PCR

Vector copy number (VCN) analysis – VCN was determined by quantitative (q)PCR analysis targeting human immunodeficiency virus-1 packaging signal (HIV-1 ψ) and human albumin (*ALB*). Genomic DNA was extracted using the QIAamp DNA Micro Kit (Qiagen) from single-cell suspensions cultured for 9 days in SCGM supplemented with antibiotics (penicillin 100 IU/mL and streptomycin 100 μg/mL), FLT3L, SCF (both 300 ng/mL), TPO (100 ng/mL) and IL-3 (10 ng/mL). VCN was calculated as the ratio of HIV-1 ψ to *ALB* copy numbers. qPCR was performed with TaqMan universal master mix II (Thermo Fisher Scientific) and gene-specific primers and probes (see key resources table) on the QuantStudio 3 (Thermo Fisher Scientific). All reactions were run in triplicate. Thermal cycling conditions consisted of an initial incubation at 50°C for 2 min, followed by 20 s at 95°C, and 40 amplification cycles of 1 s at 95°C and 20 s at 60°C.

B-cell maturation – To assess *AICDA* expression and plasmablast-to-plasma cell differentiation, qPCR was performed to assess gene expression kinetics in ABO-$CD19^+$ cells upon CD40-mediated activation in ABOs using CD40L-expressing MS-5 cells. Cells were harvested on days 0, 2, 4, 6, and 11 of culture. qPCR was conducted using gene-specific primers and probes for *GAPDH*, *AICDA*, *PRDM1*, and *PAX5* (see key resources table) under the same cycling conditions as described for VCN analysis.

PrimeFlow analysis

The PrimeFlow RNA assay (Thermo Fisher Scientific) was performed according to manufacturer's instructions on transduced and untransduced HSPCs derived from patients with RAG1-SCID 9 days post-transduction to assess coRAG1 transduction efficiency.[98] All buffers are included in the PrimeFlow RNA assay kit and specific target probe sets for huRPL13A and coRAG1 were designed and obtained from Thermo Fisher. Cells were fixed for 30 min at 4°C with Fixation buffer 1 and subsequently with Fixation buffer 2 for 1 h at RT. Samples were protected from light during fixation. A hybridization step was performed by incubating the cells with the appropriate target probe sets for 2 h at 40°C. Samples were stored overnight at 4°C protected from light. The next day, pre-amplification and amplification of the hybridized probes were performed by two consecutive incubations of 90 min at 40°C with the

pre-Amplification mix and Amplification mix. Finally, cells were incubated with the label probe sets for 1 h at 40°C. Samples were acquired on a BD FACSCanto II flow cytometer, and data were analyzed using FlowJo software.

GeneScan analysis

Genomic DNA was extracted using the QIAamp DNA Micro Kit from single-cell suspensions of digested ABOs (day 35 for UCB and day 42 for mPB). Per reaction, 50 ng genomic DNA was added to the primer mixes – which include Ampli*Taq* Gold polymerase (Invivoscribe) – for *IGH* (containing V_H FR1-J_H, IGH V_H FR2-J_H, IGH V_H FR3-J_H primers) and *IGK* (containing V_K-J_K, and V_K/intron-Kde primers) loci.[85] Thermal cycling conditions consisted of an initial incubation at 95°C for 10 min, followed by 35 amplification cycles of 30 s at 94°C, 30 s at 60°C, and 1 min at 72°C, ending with 10 min at 72°C. PCR products were denatured using Hi-Di formamide together with GeneScan 500 ROX size standard (Thermo Fisher Scientific). Samples were denatured in a thermal cycler for 2 to 5 min at 95°C followed by 5 min at 4°C. All reactions were run in duplicate. GeneScan results were analyzed using Genemarker software (Thermo Fisher Scientific, v3.0.0).

QUANTIFICATION AND STATISTICAL ANALYSIS

Statistical analyses (except for scRNA-seq analysis) were performed using GraphPad Prism (version 9.3.1) as indicated in the figure legends. Statistical analyses and data representation are described in the figure legends. In case data did not follow a normal distribution, a logarithmic transformation of the data was performed. A two-sided $p < 0.05$ was considered statistically significant. A one-sided $p < 0.05$ was used to test significant enrichment of the module scores within clusters, using the average module score of all cells in the dataset as reference value.

Cell Reports Medicine, Volume *7*

Supplemental information

ABO: A 3D stroma-supported culture platform enabling full human B-lymphopoiesis for disease modeling and gene therapy development

Merijn Braams, Martijn Cordes, Sandra A. Vloemans, Bas de Mooij, Sandra de Bruin-Versteeg, Ashley Wachtmeester, Anton W. Langerak, Karin Pike-Overzet, Frank J.T. Staal, Kirsten Canté-Barrett, and Sander de Kivit

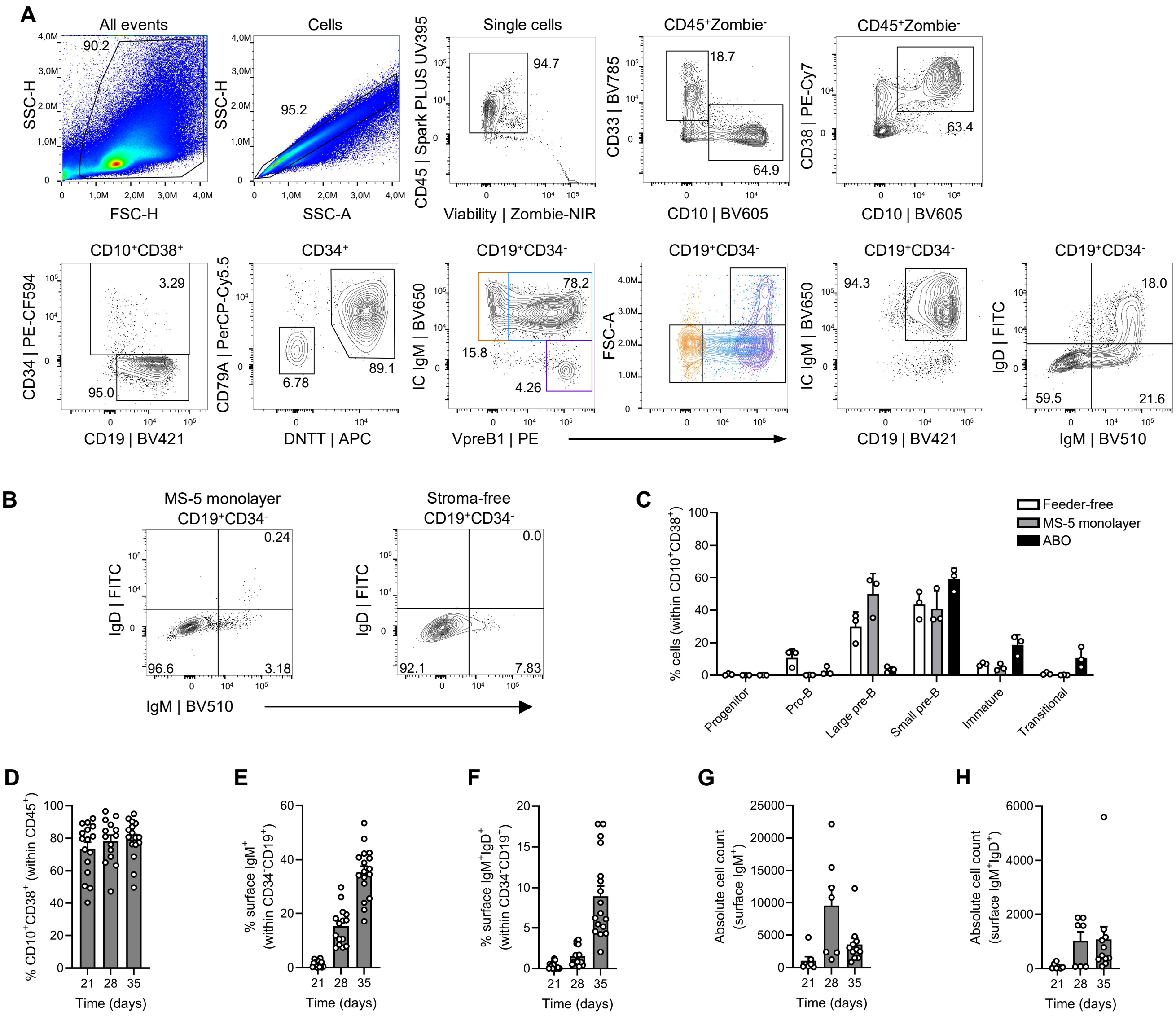

Figure S1. Characterization and reproducibility of B-cell development in ABOs. Related to Figure 1.

(**A**) Gating strategy used to identify the B-cell developmental stages.

(**B**) Representative flow cytometric plots showing the frequencies of IgM^+IgD^- immature and IgM^+IgD^+ transitional B cells in day 35 cultures.

(**C**) Distribution of HSPCs developing along the B-cell developmental trajectory at day 35 of culture.

(**B, C**) Data represent n=3 individual donors in independent cultures.

(**D-F**) Quantification of the frequencies of lymphoid committed $CD10^+CD38^+$ (**D**), IgM^+ (**E**) and IgM^+IgD^+ cells in ABOs at indicated time points (**F**). Data represent n=17 individual donors in independent cultures.

(**G, H**) Quantification of absolute counts of IgM^+ (**G**) and IgM^+IgD^+ (**H**) cells harvested from 2 ABOs at indicated time points. Data represent n=11 individual donors in independent cultures.

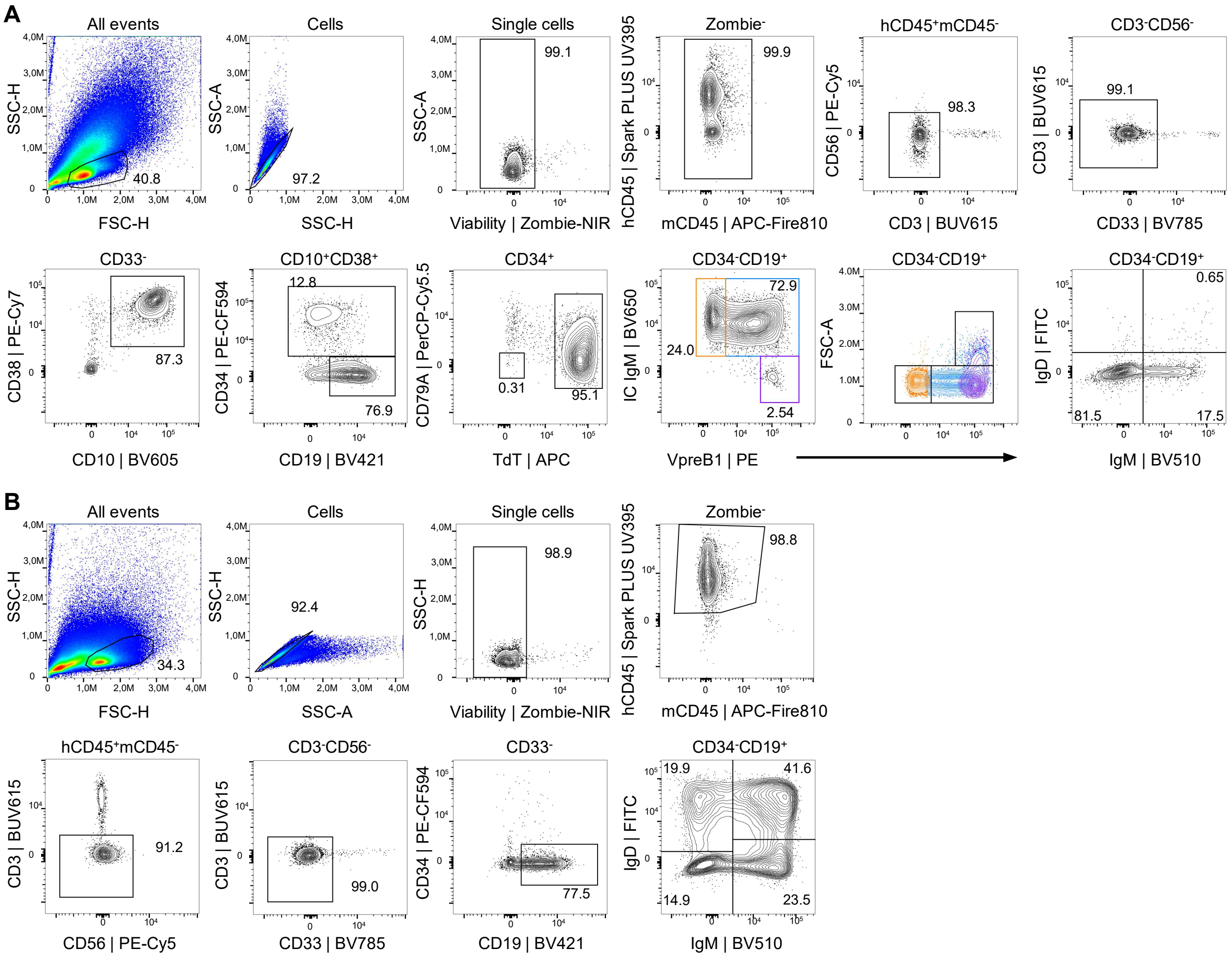

Figure S2. Flow cytometric analysis of the B-cell developmental stages in humanized NSG mice material. Related to Figure 2.

(**A, B**) Gating strategy used for the analysis of BM (**A**) and spleen (**B**). (mCD45, murine CD45; hCD45, human CD45)

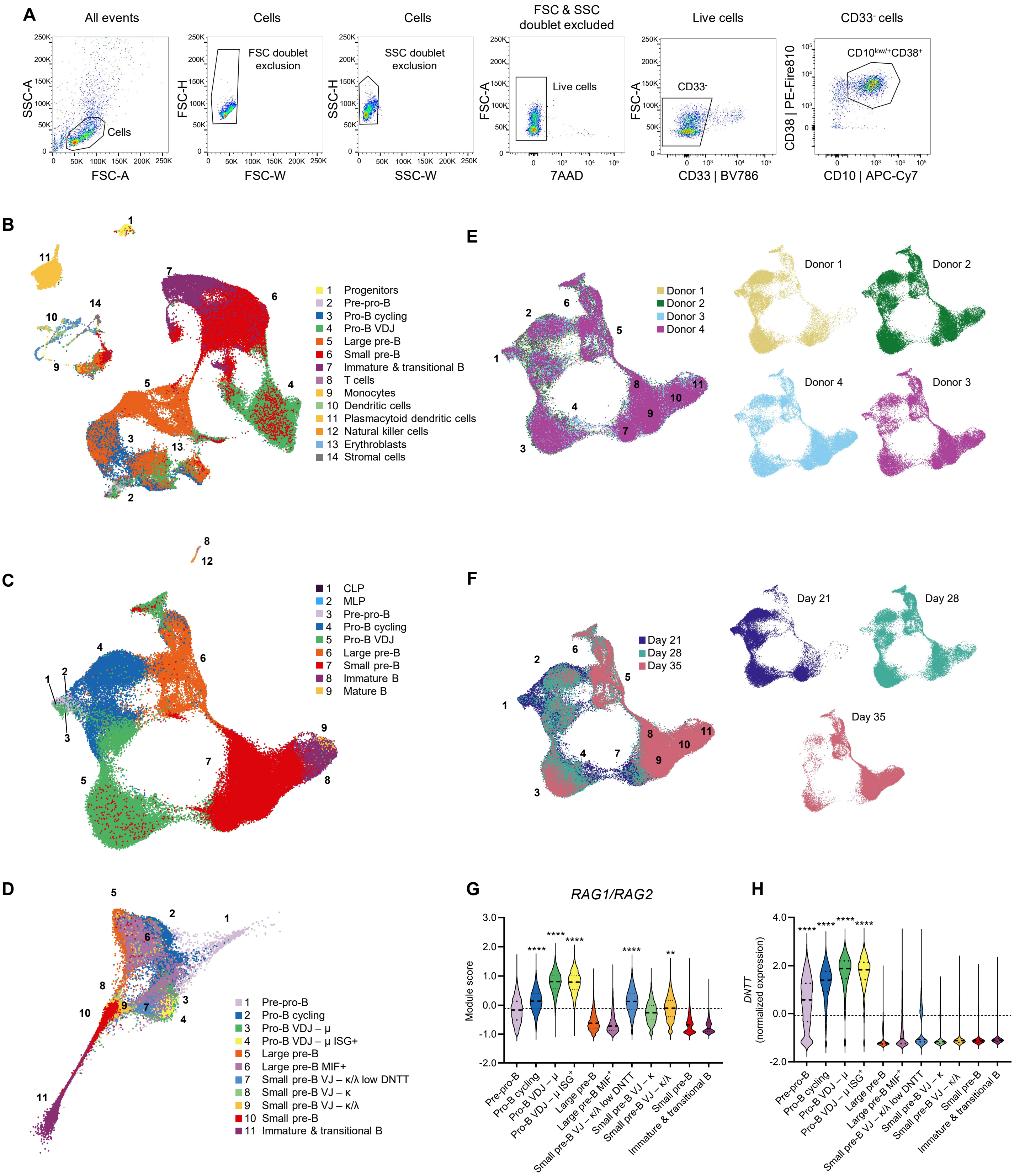

Figure S3. ScRNA-seq analysis of B-cell development in ABOs. Related to Figure 3.
(**A**) Gating strategy used for sorting of CD10⁺CD38⁺ cells from day 21-35 ABOs.
(**B, C**) Projection of clusters annotated as described by Zeng *et al.* [S1] onto the UMAP derived from scRNA-seq data of day 21-35 ABOs before subtracting non-B cells (**B**), and after filtering for B cells (**C**).
(**D**) Projection of scRNA-seq data of day 21-35 ABOs onto the UMAP derived of healthy human BM [S1].
(**E, F**) Projection of clusters annotated based on (**E**) individual donors and (**F**) time of ABO culture onto the UMAP of day 21-35 ABOs. Numbers represent clusters as identified in **Figure 3B**.
(**G, H**) Violin plots illustrating recombination activity (**G**) based on *RAG1/2* expression and relative expression of *DNTT* (**H**) within each cluster annotated. Statistical analysis was performed using a one-sample *t*-test (**** $p<0.0001$, **$p<0.01$, dashed line: average module score/expression level of the data set).
(**B-H**) Data represent n=4 individual donors in independent cultures.

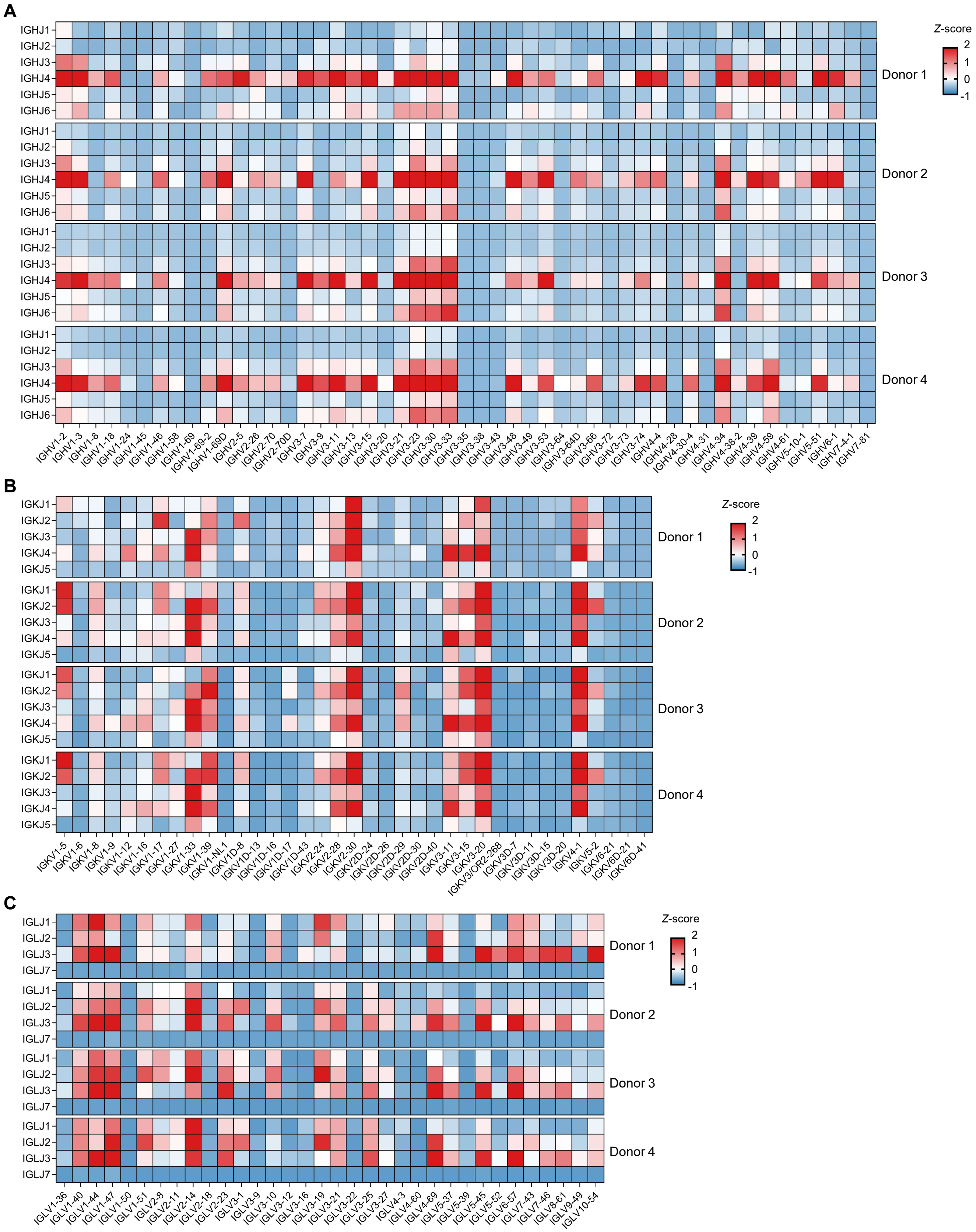

Figure S4. Analysis of V- and J-gene segments usage among individual donors. Related to Figure 4.
(**A-C**) Heatmaps showing V- and J-gene segment usage during Ig heavy *(IGH*) chain (**A**), Igκ (*IGK*) (**B**) and Igλ (*IGL*) light chain rearrangements (**C**), separated by each individual donor within immature and transitional B cells in day 35 ABOs. Z-scores calculated from V-J gene pair frequencies are color coded.

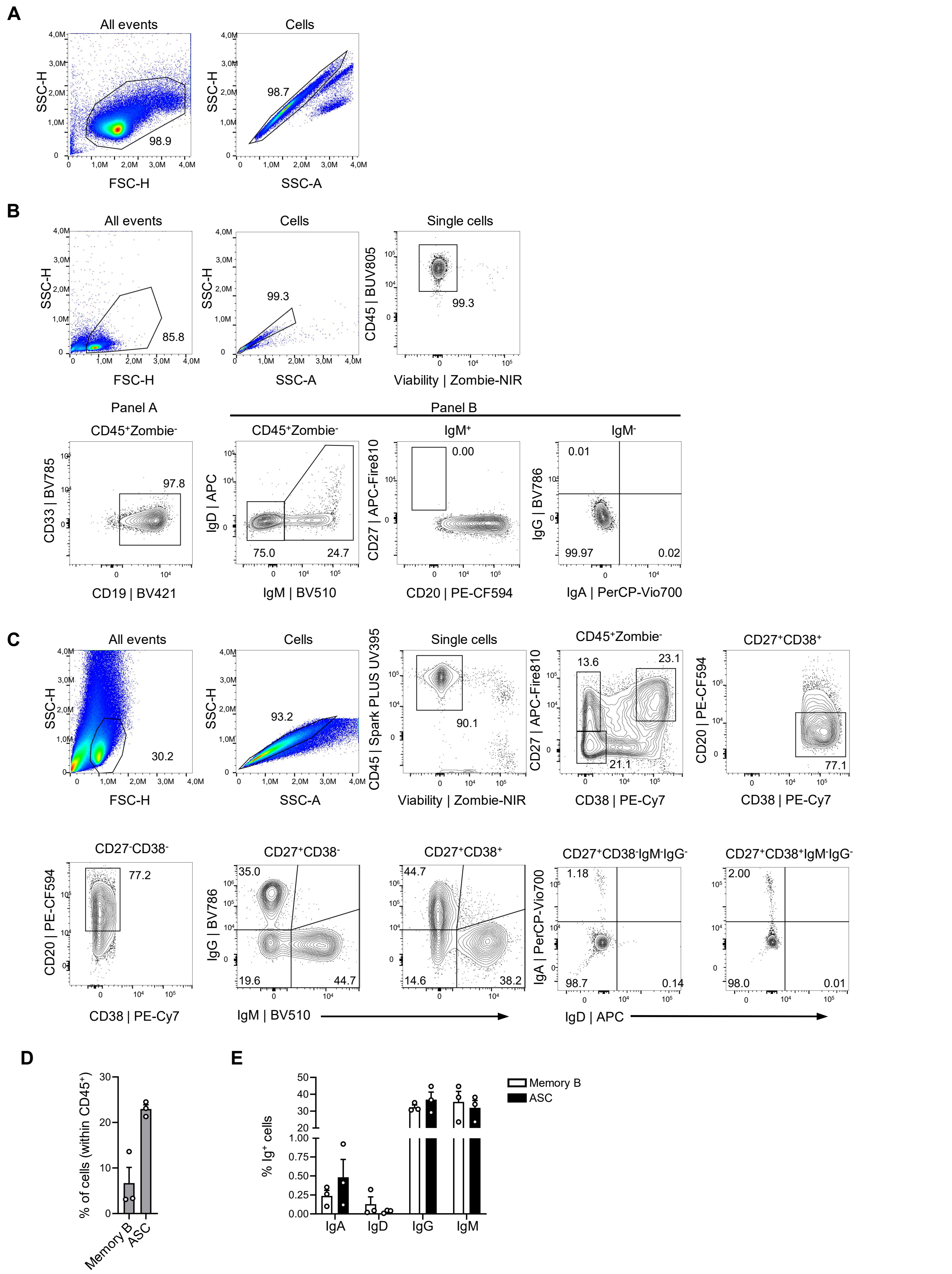

Figure S5. Functional assessment and gating strategies of activated ABO-CD19^{+} B cells. Related to Figure 5.
(**A, B**) Gating strategies prior to determining Ca^{2+} flux (**A**) in day 35 ABO-CD19^{+} cells. (**B**) Gating strategy used for assessing purity (panel A) and maturation state (panel B) of CD19^{+} cells isolated from day 35 ABOs.
(**C**) Gating strategy used for the flow cytometric analysis of antibody-secreting cells (ASCs) at day 11 post-stimulation of ABO-CD19^{+} cells using UCB-derived HSPCs.
(**D**) Quantification of the frequencies of CD27^{+}CD38^{-} memory B cells and CD27^{+}CD38^{hi}
(**E**) Distribution of IgM^{+} and Ig class-switched memory B cells and ASCs at day 11 post-stimulation of ABO-CD19^{+} cells using UCB-derived HSPCs.
(**D, E**) Data are presented as mean ± SEM and represent n=3 individual donors in independent cultures.

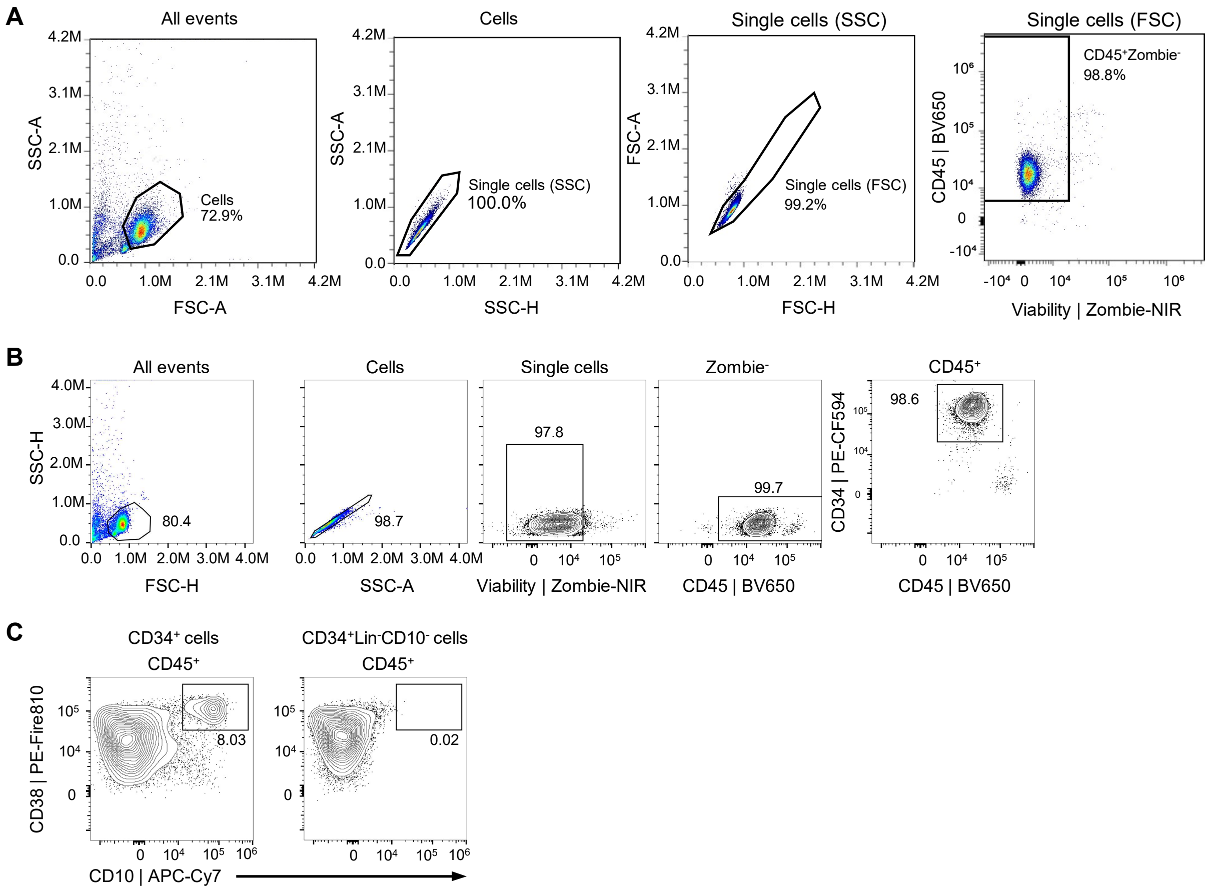

Figure S6. Depletion of B-cell progenitors does not influence B-cell development in ABOs. Related to Figure 6.

(**A**) Gating strategy used prior to opt-SNE analysis.

(**B**) Gating strategy used for quantification of $CD10^{+}$ cells depletion.

(**C**) Representative flow cytometric analysis of CD10 expression on HSPCs before (left) and after depletion of $Lin^{+}CD10^{+}$ cells (right) by MACS purification. Data represents n=3 individual donors in independent cultures.

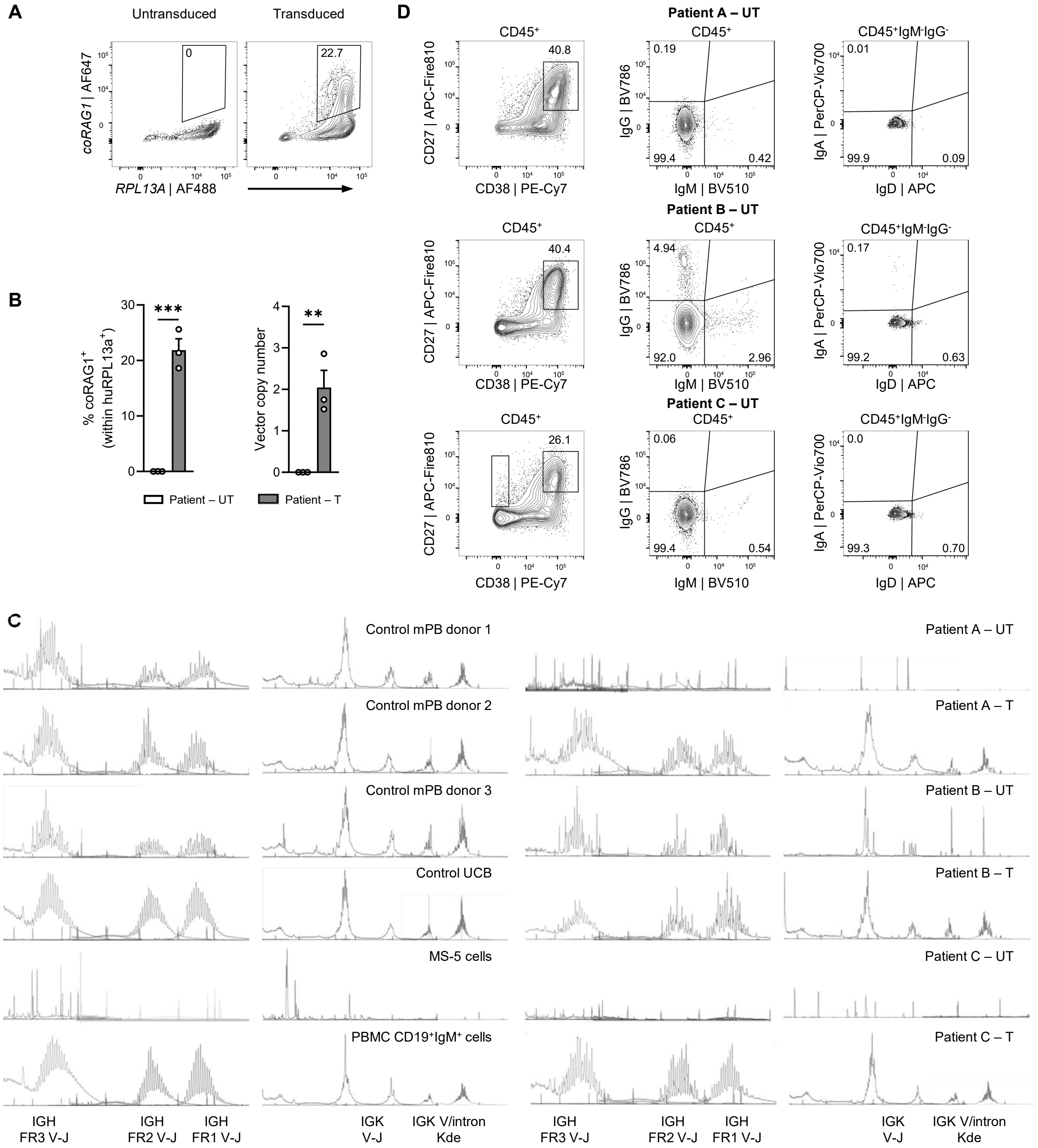

Figure S7. B-cell development from healthy control or RAG1-deficient mPB-derived HSPCs ABOs. Related to Figure 7.
(**A**) Representative flow cytometric analysis showing coRAG1 transduction efficiency (RPL13A; housekeeping gene) assessed by the PrimeFlow assay in RAG1-deficient mPB-derived HSPCs.
(**B**) Quantification of the frequency of coRAG1$^+$ (left) and corresponding vector copy numbers (right) in 9-day cultured RAG1-deficient mPB-derived HSPCs.
(**A**, **B**) Data are presented as mean ± SEM and represent n=3 from individual donors in independent cultures. Statistical analysis was performed using an unpaired Students *t*-test (**p<0.01, ***p<0.001).
(**C**) Visualization of V_H-J_H rearrangements across the conserved framework regions (FR1-3) of the *IGHV* locus and V_K-J_K rearrangements in the *IGKV* locus including V/intron Kappa deletion elements (Kde) by GeneScan analysis from healthy control, untransduced (UT), and coRAG1 transduced (T) RAG1-deficient mPB HSPCs-derived ABOs harvested on day 42, UCB HSPCs-derived ABOs (representative of n=4) harvested on day 35. (Negative control: MS-5 cells; positive control: PBMC-derived CD19$^+$IgM$^+$ cells.
(**D**) Flow cytometric analysis showing the presence CD27$^+$CD38$^+$ ASCs upon CD40 stimulation of ABO-CD19$^+$ cells for 11 days.

Patient with RAG1-SCID	Age	Sex	Mutation coding DNA	Protein sequence change	Zygosity	Clinical phenotype	
						T cells	B cells
Pt. A	4 mo.	M	c.[519del];[519del]	p.[(Glu174fs)]; [(Glu174fs)]	Homozygous	severely decreased	severely decreased
Pt. B	5 mo.	M	c.256_257del	p.(Lys86Valfs*33)	Homozygous	severely decreased	decreased
Pt. C	8 mo.	F	c.2096G>A	p.Arg699Gln	Homozygous	undetectable	severely decreased

Table S1. RAG1 mutations in HSPCs from patients with RAG1-SCID used in the study. Related to Figure 7. Mo., months; M, male; F, female.

Supplemental references

S1. Zeng, A.G.X., Iacobucci, I., Shah, S., Mitchell, A., Wong, G., Bansal, S., Chen, D., Gao, Q., Kim, H., Kennedy, J.A., et al. (2025). Single-cell Transcriptional Atlas of Human Hematopoiesis Reveals Genetic and Hierarchy-Based Determinants of Aberrant AML Differentiation. Blood Cancer Discov 6, 307– 324. 10.1158/2643-3230.BCD-24-0342.
